# Preparation and catalytic application of two different nanocatalysts based on hexagonal mesoporous silica (HMS) in synthesis of tetrahydrobenzo[b]pyran and 1,4-dihydropyrano[2,3-c]pyrazole derivatives

**DOI:** 10.1038/s41598-022-26605-0

**Published:** 2022-12-21

**Authors:** Sahar Abdolahi, Fatemeh Gholamian, Maryam Hajjami

**Affiliations:** 1 Department of Chemistry, Faculty of Science, Ilam University, P.O. Box 69315516, Ilam, Iran; 2 Department of Organic Chemistry, Faculty of Chemistry, Bu-Ali Sina University, Hamedan, 6517838683 Iran

**Keywords:** Organic chemistry, Catalysis, Catalyst synthesis, Heterogeneous catalysis

## Abstract

The present study describes the synthesis, characterization, and investigation of catalytic activity of xanthine-Ni complex (Xa-Ni) and 4-phenylthiosemicarbazide-Cu complex **(**PTSC-Cu) incorporated into functionalized hexagonal mesoporous silica (HMS/Pr-Xa-Ni and HMS/Pr-PTSC-Cu). These useful mesoporous catalysts had been synthesized and identified using various techniques such as FT-IR, XRD, adsorption–desorption of nitrogen, SEM, TEM, EDX-Map, TGA, AAS and ICP. These spectral techniques successfully confirmed the synthesis of the mesoporous catalysts. The catalytic activity of HMS/Pr-a-Ni (Catalyst A) and HMS/Pr-PTSC-Cu (Catalyst B) were evaluated for synthesis of tetrahydrobenzo[b]pyran and 1,4-dihydropyrano[2,3-c]pyrazole derivatives. HMS/Pr-PTSC-Cu exhibited higher efficiency in green media under milder reaction condition at room temperature. Furthermore, the synthesized nanocatalysts, exhibited appropriate recoverability that can be able to reuse for several times without significant loss of catalytic activity.

## Introduction

In recent years, with the development of nanoscience and nanotechnology, attractive possibilities emerged for synthesis of various new silica catalysts. Mesoporous silica (2 nm < pore diameters > 50 nm), is the most popular mesoporous molecular material^1^ that due to their structural characteristics, such as high surface areas and pore volumes, have found variety applications, such as adsorbents, catalyst supports, drug delivery systems and biosensors^2^. Among those mesoporous materials, hexagonal mesoporous silica (HMS) with wormlike mesoporosity, uniform and narrow pore size distribution, high surface area and pore volume, short channel, thermal stability, easily synthesized and functionalized has found promising applications as a support for synthesis of heterogeneous catalysts^3,4^. In addition, HMS easily synthesis using a cheaper primary alkylamine^5^ at room temperature and pH-independent condition, which makes the method robust, reproducible and industrially possible^6^

Also, in opposite of MCM and SBA mesoporous silica, HMS has indicated commendable outputs in the catalysis research area^6^. Design and development of multicomponent synthetic strategies which are in accordance with the principle of green chemistry and using novel and heterogenous catalyst led to the integration of a variety of novelty in their line of investigation^7^.

Recently, the researches regarding synthesis and characterization of metal modified HMS mesoporous materials published in the literature such as: NiMoW/HMS and NiMoW/Al-HMS^8^, Cu-Ag/HMS^9^, FeC_4_Pc-HMS^10^, HMS-CPTMS-Cy-Pd^4^ and HMS/Pr-Rh-Zr^11^. In these articles, HMS was applied as an efficient support for synthesis of novel catalysts, that promoted the rate of reaction.

One-pot multi-component reactions (MCR) are processes “in which more than two organic moieties are joined in one step to obtain carbon–carbon and carbon-heteroatom bond”. This synthetic strategy does not need to separation and purification of the intermediates. In comparison to traditional multistep protocols, one-pot multi-component reactions have both economic and environmental benefits such as decreasing time, saving costs, energy^12^, increasing atom economy^7^, reducing waste production, high efficiency and experimental simplicity^13^. Therefore, they are economically and environmentally suitable methodology^12^ and often proceed with excellent chemoselectivities^13^.

Pyranopyrazole compounds, oxygen- and nitrogen-ring fused heterocycles, have become important due to their pharmacological and biological properties^14^. Additionally, pyrazole and its derivatives find applications as biodegradable agrochemicals^13^. Among the medicinal properties of these compounds can be mentioned to anti-inflammatory^15^, antioxidant^16^, anti-bacterial^17^ and anti-tubercular agents^18^.

Also, benzopyran and its derivatives as one of the important groups of compounds, have appealed to researchers. Their biological and medicinal properties led to attracting much attention. These heterocyclic frameworks existence in structure of some of the natural products and applied in treatment of disease such as Schizophrenia, Myoclonus, Alzheimer, Huntington, Parkinson, AIDS-associated dementia and Down syndrome^19–22^. Additionally, another application of benzopyran derivatives consists of using in perfumes, cosmetics, agrochemicals and in food as additives^23^, photoactive materials^19^ and pigments^24^.

Thus, in continuation of our ongoing research^4,25–27^, considering the importance of these heterocyclic compounds, we sought to find the effect of new synthesized catalysts for the preparation of these biologically valuable heterocycles.

## Results and discussion

### Preparation and characterisation of catalysts A and B

**Figure 1:**
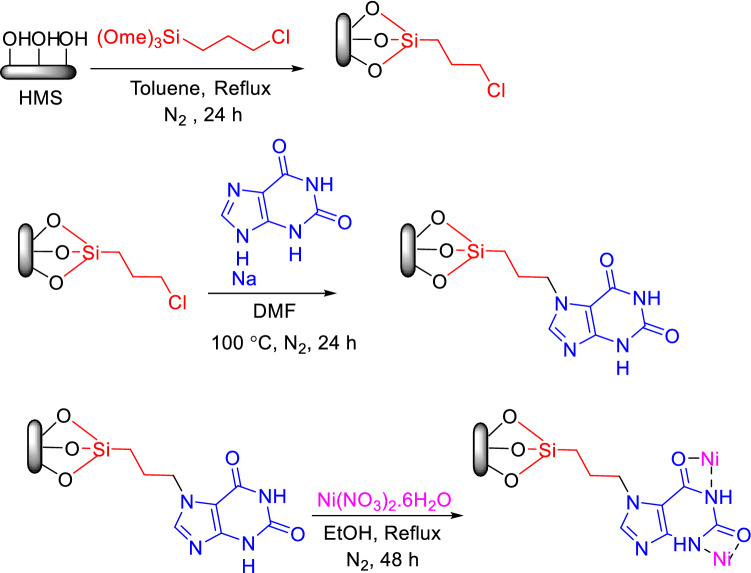
General procedure for the synthesis of HMS/Pr-Xa-Ni.

For both catalysts, at first, HMS was synthesized similar to a previously reported research^4^. Then, HMS was functionalized by 3‐chloropropyltrimethoxysilane and HMS/Pr was obtained. In the next step, (for Catalyst A) xanthine sodium was reacted with HMS/Pr. Ultimately, for synthesis of final catalyst, Ni(NO_3_)_2_.6H_2_O was applied to afford HMS/Pr-Xa-Ni. All steps for the synthesis of HMS/Pr-Xa-Ni are presented in Fig. 1.

**Figure 2:**
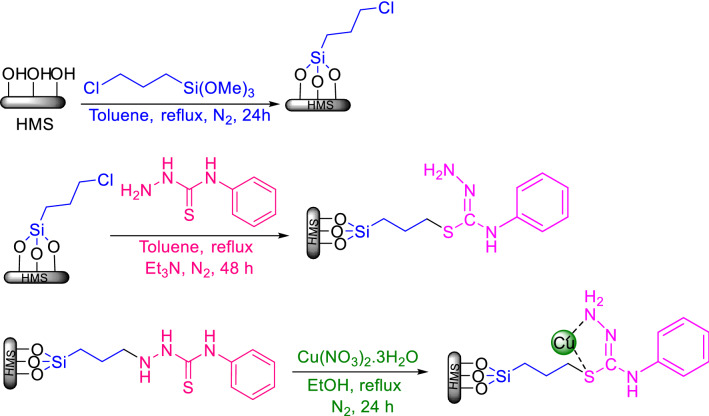
General procedure for synthesis of HMS/Pr-PTSC-Cu.

The method for synthesis of HMS/Pr-PTSC-Cu (Catalyst B) is shown in Fig. 2. After preparation of HMS/Pr, 4-phenylthiosemicarbazide and Et_3_N was added to HMS/Pr and HMS/Pr-PTSC was obtained. Finally reaction of Cu(NO_3_)_2_.3H_2_O with HMS/Pr-PTSC lead to synthesis of catalyst B.

After fabrication of catalysts, for their confirmation, we were applied different techniques such as FT-IR, XRD, TGA, SEM, TEM, EDX-Map, adsorption-desorption of nitrogen, AAS and ICP. All these analyses interpreted for catalyst A and B respectively in next section.

**Figure 3:**
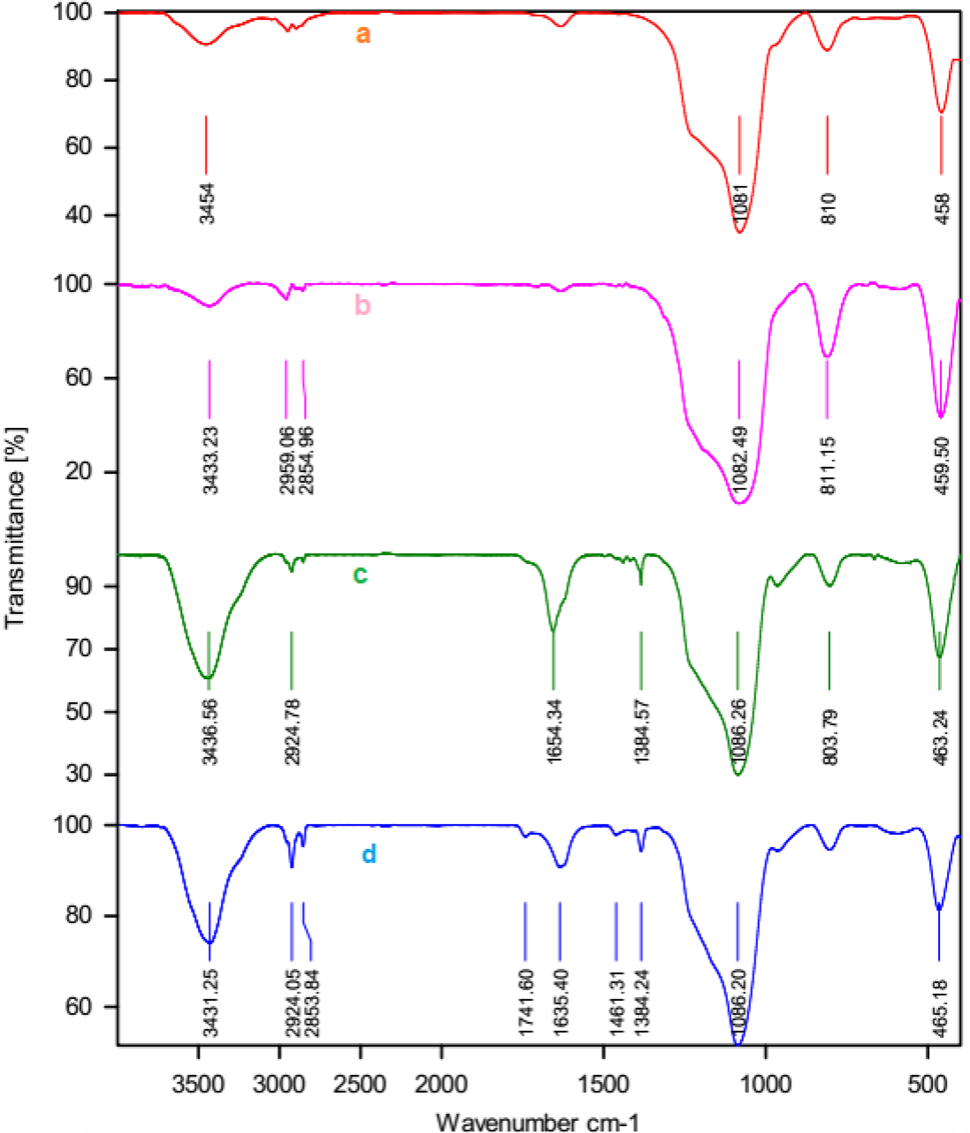
FT-IR spectra of HMS (a) and HMS/Pr (b), HMS/Pr-Xa (c) and HMS/Pr-Xa-Ni (d).

The FT-IR spectroscopy (Fig. 3) was applied for confirmation of functional groups of the synthesized compounds such as HMS (a) and HMS/Pr (b), HMS/Pr-Xa (c) and HMS/Pr-Xa-Ni (d). FT-IR spectra of HMS (a) indicated the peaks at 3454 cm^-1^ contributed to silanol group and the peaks at 810 cm^−1^ and 1081 cm^−1^ are attributed to Si–O–Si symmetric and asymmetric stretching vibration respectively^6^. The C–H stretching vibrations, in spectrum of HMS/Pr (b), are appearing in the range of 2854–2959 cm^−1^. As shown in spectrum of HMS/Pr-Xa (c), the peak at 3436 cm^−1^ could be ascribed to the N–H stretching vibration also carbonyl of amid group contributed to xanthine, located at 1654 cm^−1^. In the spectrum of HMS/Pr-Xa-Ni (d) this peak shifts to 1635 cm^−1^ due to the coordination with the metal of Ni.

**Figure 4:**
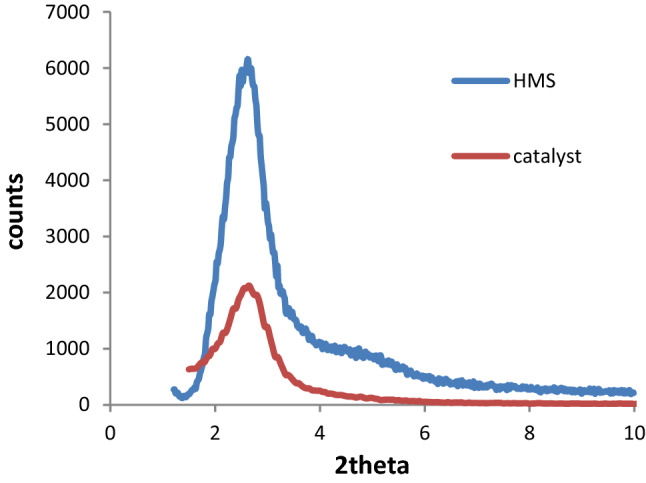
XRD patterns of HMS and HMS/Pr-Xa-Ni.

Low angle X-ray diffraction (XRD) patterns of HMS and HMS/Pr-Xa-Ni are indicated in Fig. 4. In these patterns, there was distinct peak at 2θ angles about of 2.6^4^. After functionalization, the hexagonal structure of HMS was preserved also the location of peak was consistent with the standard diffraction pattern of HMS. As shown in Fig. 4, the decrease in intensity of the characteristic diffraction peak belongs to catalyst, rather than HMS, was confirmed that organic moieties on the pore wall of HMS was successfully immobilized. The reason can also be known as the decrease in the mesoscopic order of the materials.

**Figure 5:**
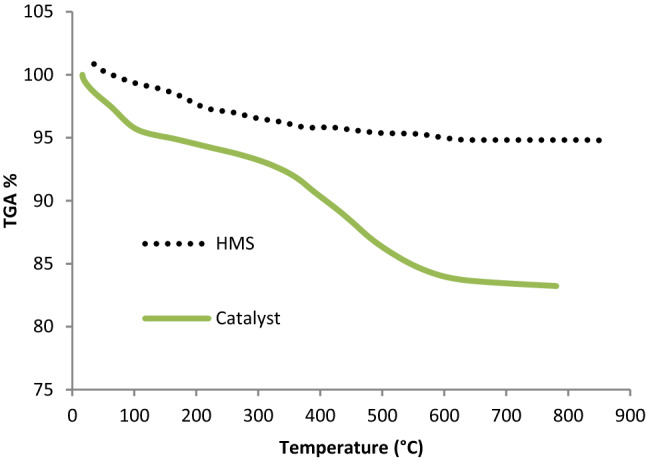
Thermogravimetric curves of HMS and HMS/Pr-Xa-Ni.

The thermo-gravimetric analysis (TGA) was applied for investigation of the thermal stability of HMS/Pr-Xa-Ni and determination of the amount of organic groups incorporated into HMS. As shown in Fig. 5, the weight loss below 150 °C contributed to the removal of physically adsorbed water and organic solvent. Immobilization of Pr-Xa-Ni in the surface of pores was revealed with their decomposition, that weight loss about 12% was indicated at temperatures 150–600 °C. Also, Ni content loaded in modified HMS in the HMS/Pr-Xa-Ni was 0.09 mmol g^−1^ that defined by ICP-AES analysis.

**Figure 6:**
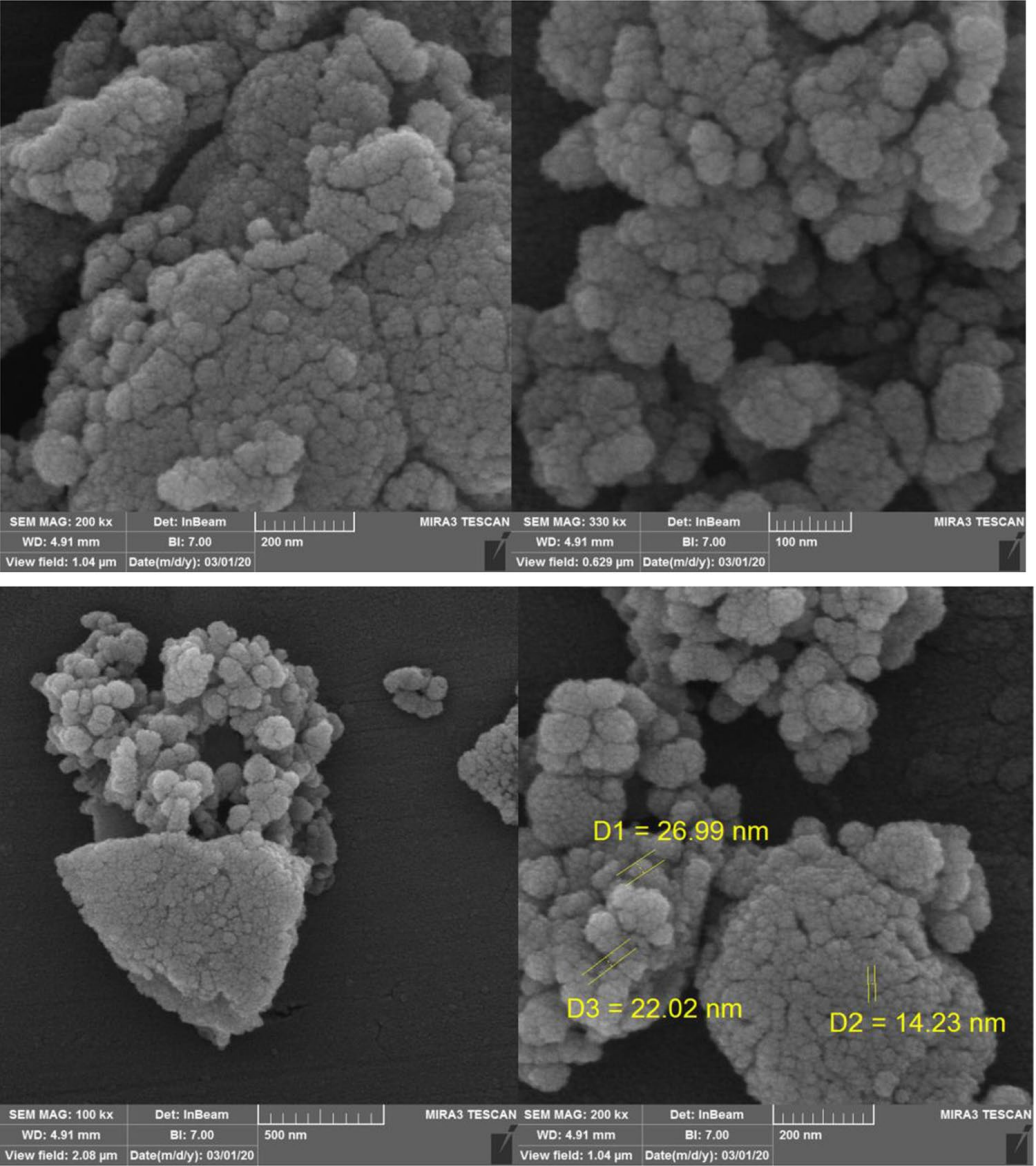
SEM images of HMS/Pr-Xa-Ni.

**Figure 7:**
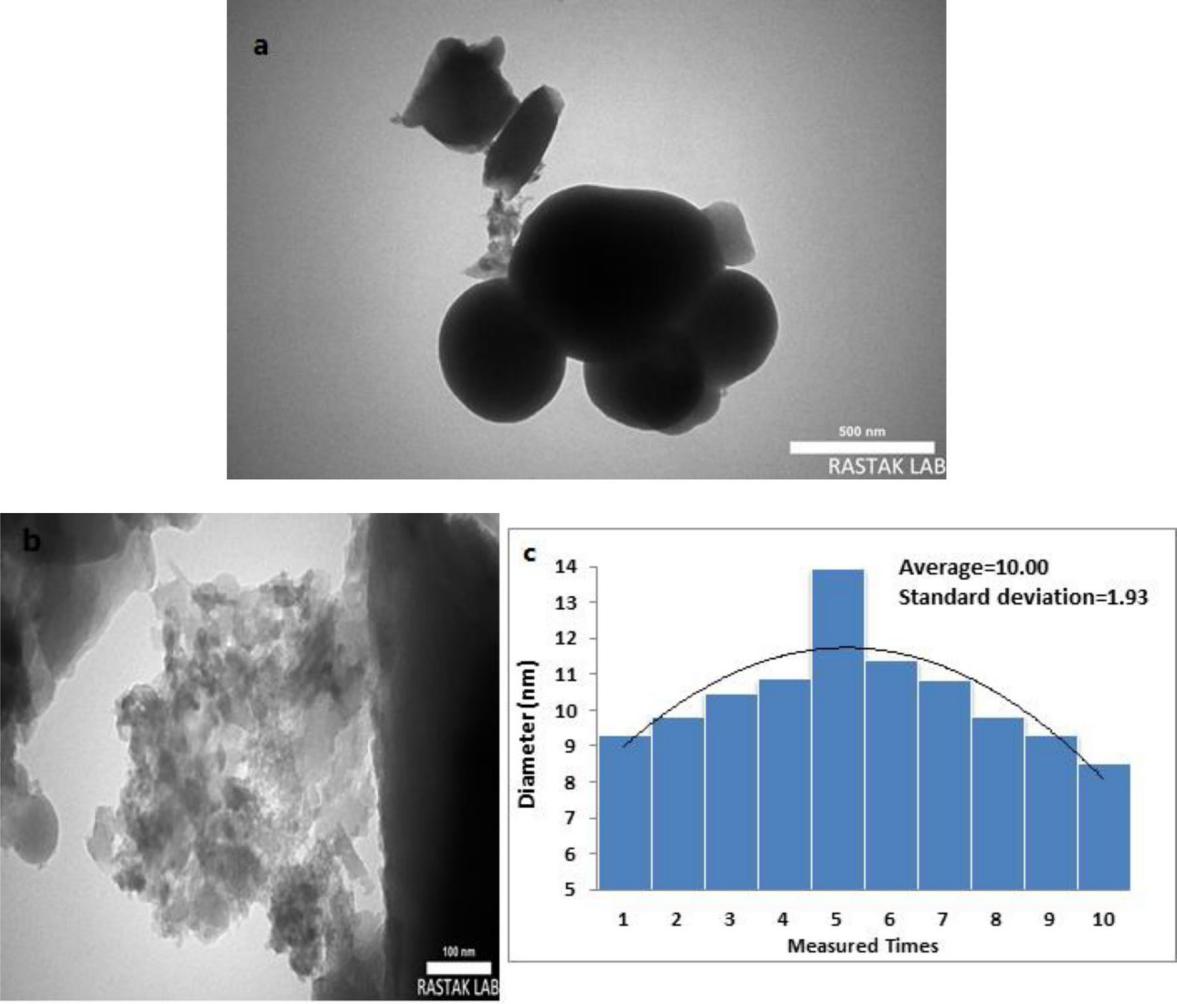
TEM images of HMS/Pr-Xa-Ni (**a,b**), particle size distribution histogram of HMS/Pr-Xa-Ni (**c**).

The morphology and particle size of the synthesized catalyst was defined by a scanning electron microscopy (SEM) (Fig. 6) and TEM analysis (Fig. 7). Figure 6 illustrates the SEM images of HMS/Pr-Xa-Ni. This analysis has shown that the prepared catalyst has regular and ordered structure. Also, the SEM images illustrated that synthesized nanocatalyst has nanometer-sized particles with an average diameter less than 27 nm. The nanocatalyst A sample is further studied by transmission electron microscopy (TEM) to obtain insight into its structural and morphological features. The nanocatalyst A TEM images display the distribution of catalyst nanoparticles of size below 50 nm.

The particle size distribution histogram of HMS/Pr-Xa-Ni was indicated in Fig. 7c. The average size of the particle is 10.00 nm with 1.93 nm standard deviation.

**Figure 8:**
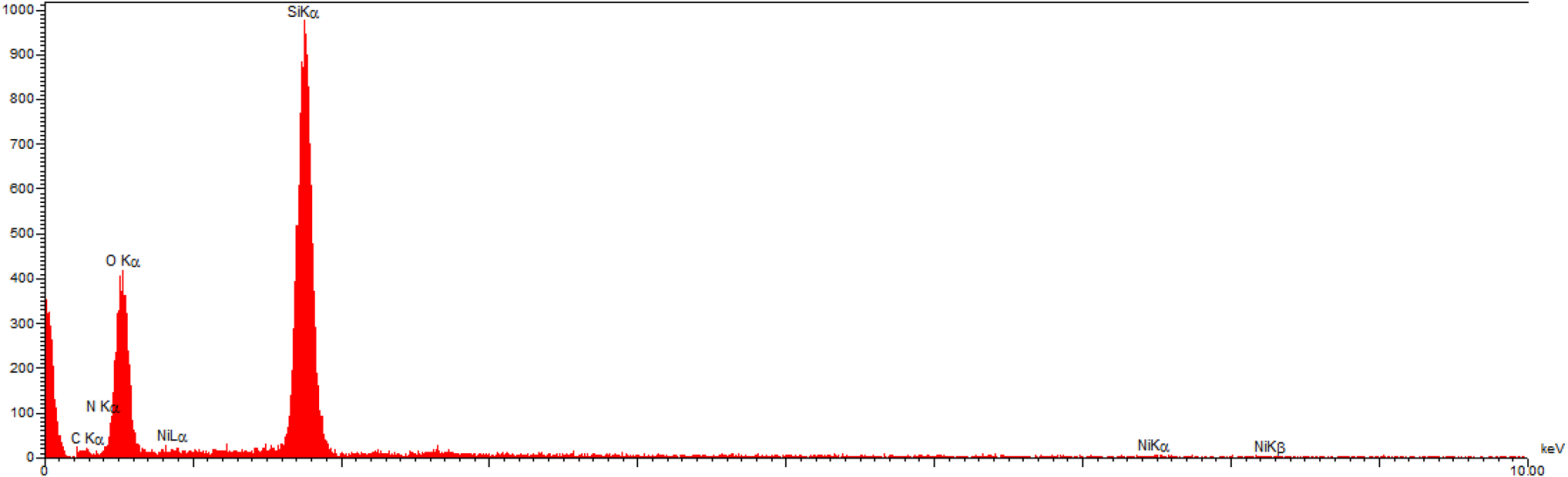
EDX spectrum of HMS/Pr-Xa-Ni.

**Figure 9:**
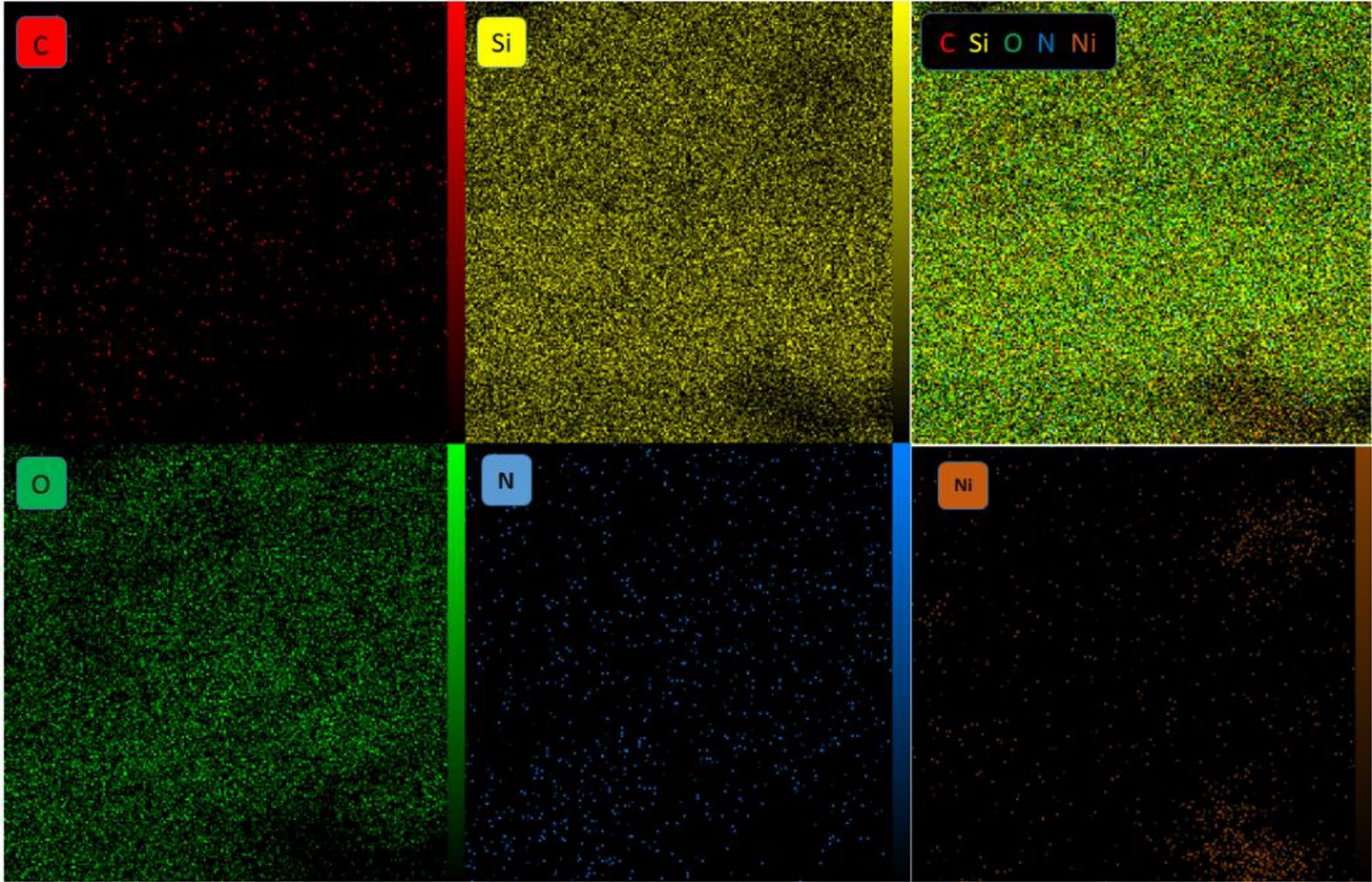
The map analysis of HMS/Pr-Xa-Ni.

EDX spectrum of synthesized catalyst characterized elemental composition in the catalyst. As shown in Fig. 8, the presence of elements of Si, O, N, C and Ni were confirmed in the EDX analysis of HMS/Pr-Xa-Ni. Also the map image revealed dispersion of all elements (Si, O, N, C and Ni) of the catalyst and confirmed the good dispersion of Ni on the surface of the HMS/Pr-Xa-Ni (Fig. 9).

**Figure 10:**
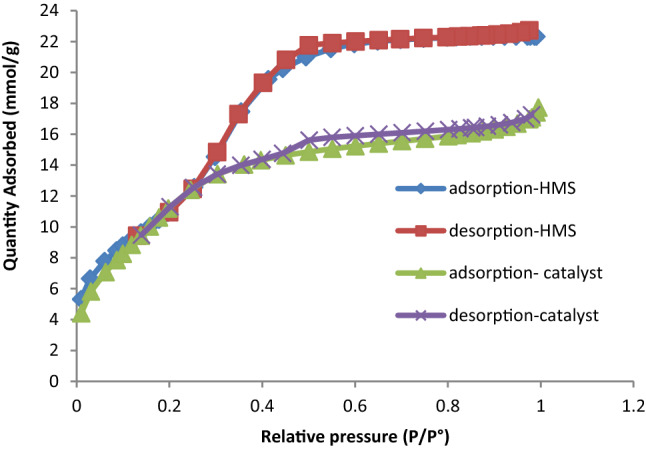
The N_2_ adsorption–desorption isotherm for HMS and HMS/Pr-Xa-Ni.

Figure 10 shown the nitrogen adsorption–desorption isotherms of HMS and HMS/Pr-Xa-Ni. According to the IUPAC classification, N_2_ adsorption/desorption isotherms of HMS and HMS/Pr-Xa-Ni showed typically reversible type IV isotherms^11^. By N_2_ isotherms, physicochemical and structural parameters of the samples were obtained containing BET surface area (S_BET_), total pore volumes (V_total_) and pore diameters (D_BJH_) (Table 1). More importantly, the decrease in S_BET_, D_BJH_ and V_total_ of HMS/Pr-Xa-Ni rather than HMS, are due to successfully immobilization of Pr-Xa-Ni in pore of the HMS.

**Table 1:** Textural properties of HMS and catalyst obtained by nitrogen adsorption/desorption analysis.

Sample name	S_BET_ (m^2^ g^−1^)	D_BJH_ (nm)	V_Total_ (cm^3^ g^−1^)
HMS	918	3.4	0.78
HMS/Pr-Xa-Ni	876	2.3	0.5

**Figure 11:**
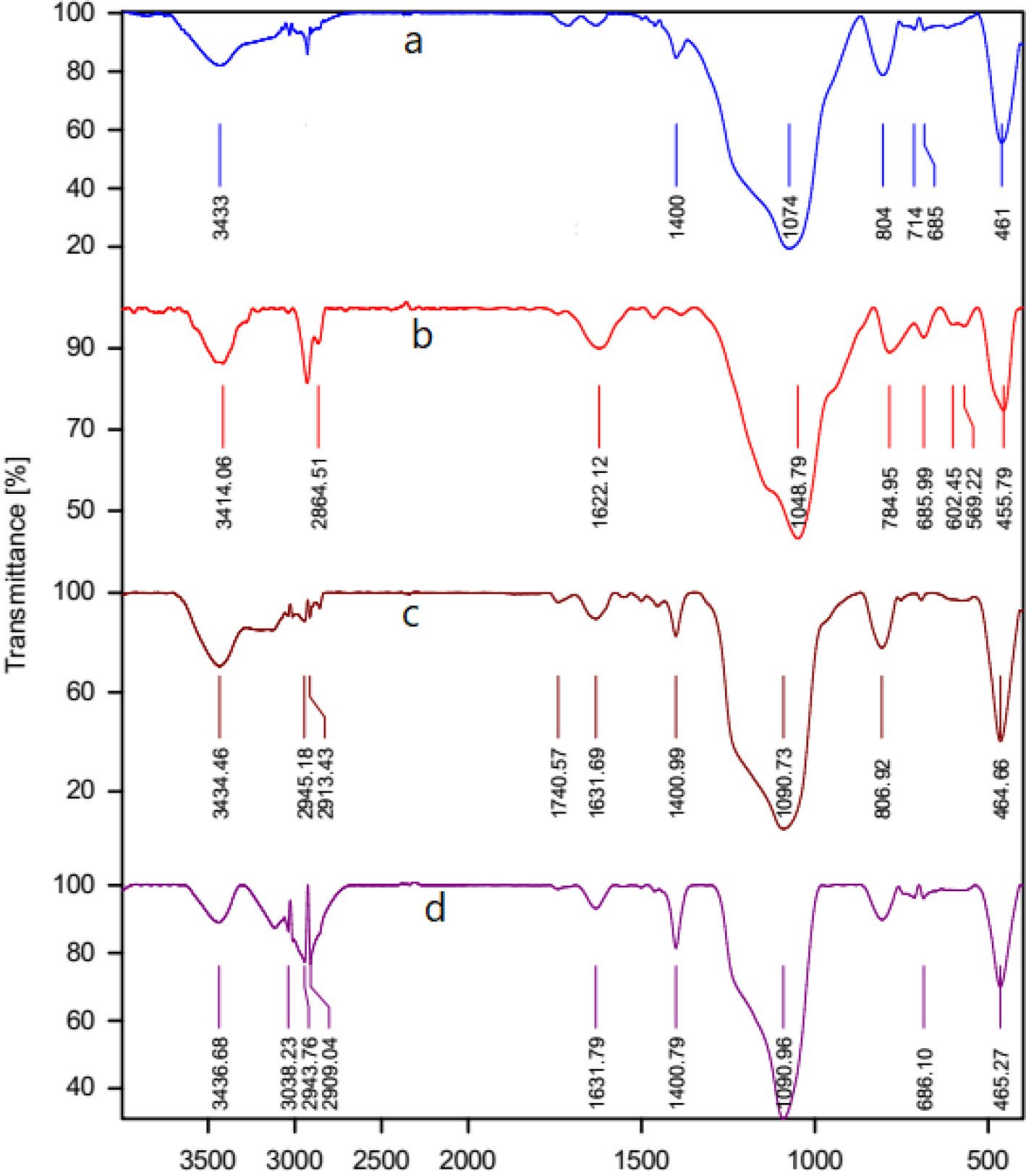
The FT-IR spectrum of HMS (a), HMS/Pr (b), HMS/Pr-PTSC (c) and HMS/Pr-PTSC-Cu (d).

For confirmation of functional groups, in catalyst B, in HMS (a), HMS/Pr (b), HMS/Pr-PTSC (c) and HMS/Pr-PTSC-Cu (d) the FT-IR spectroscopy was investigated that indicated in Fig. 11.

In the FT-IR spectra of HMS (a), the peak appeared at 3433 cm^−1^ contributed to silanol group. Also, the peaks at 804 cm^−1^ and 1074 cm^−1^ demonstrated that ascribed to Si–O–Si symmetric and asymmetric stretching vibration respectively^4^. The FT‐IR spectrum of HMS/Pr which is illustrated in Fig. 11b shows peaks at 784 cm^−1^ and 1048 cm^−1^ relating to the Si–O–Si symmetric and asymmetric stretching vibration respectively. Another peak at 2864 cm^−1^ is contributed to the C–H stretching vibrations. In the spectrum of HMS/Pr-PTSC (C) C–H stretching of the alkyl group are detected by the peaks at 2913–2945 cm^−1^. As shown in spectrum of HMS/Pr-PTSC (C) and HMS/Pr-PTSC-Cu (d), symmetric and asymmetric stretching vibration of Si–O–Si are appearing in 806 cm^−1^ and 1090 cm^−1^. Also, the peaks of 1400 cm^−1^ and 1631 cm^−1^ are contributed to (C=C) aromatic ring. In the spectrum of catalyst the existence of NH is confirmed by peak that appears in 3436 cm^−1^.

**Figure 12:**
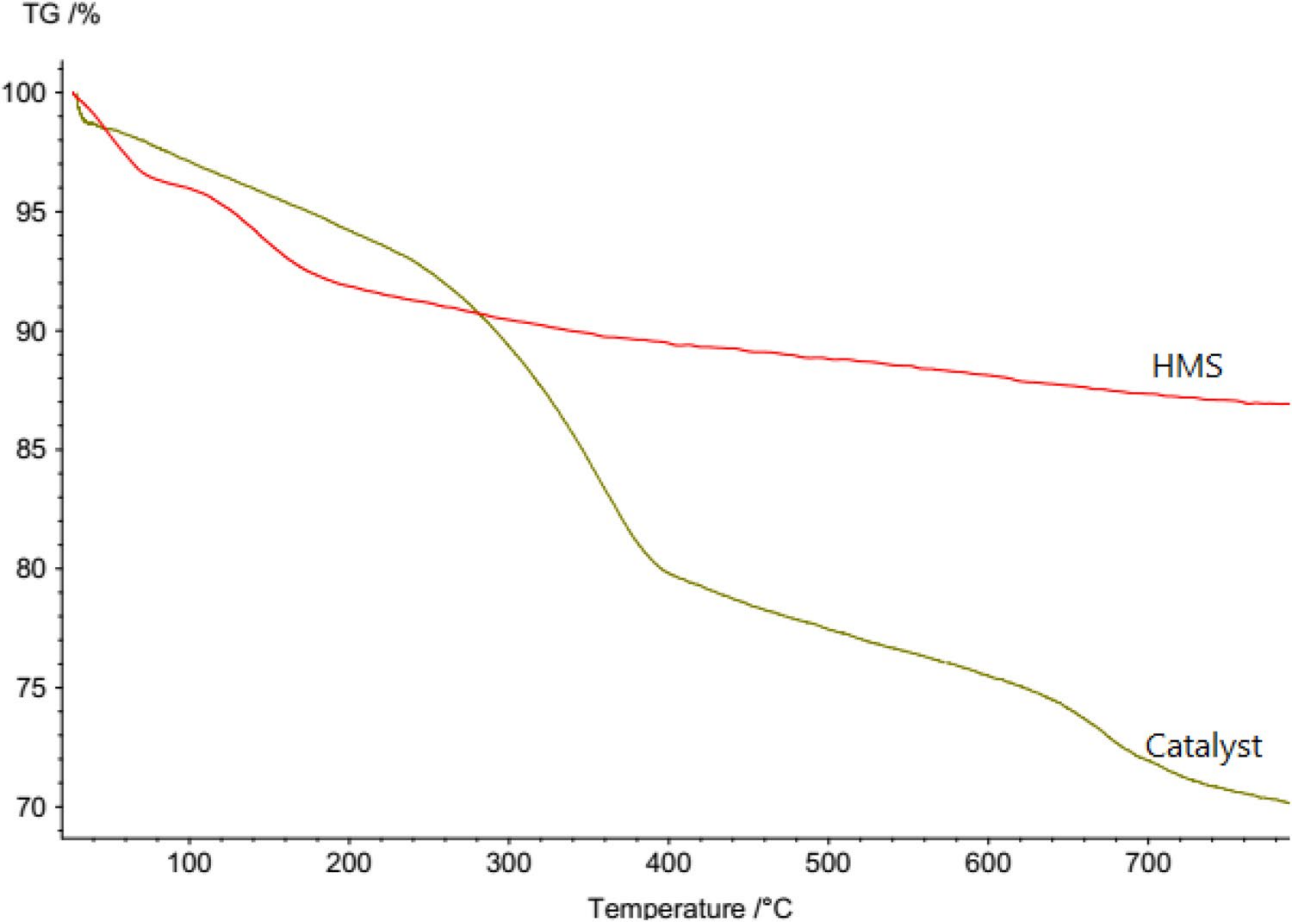
Thermogravimetric curves of HMS and HMS/Pr-PTSC-Cu.

Thermogravimetric curves of HMS and HMS/Pr-PTSC-Cu at the temperature range of 25 °C to 800 °C shown in Fig. 12. The thermal behaviour of catalyst shows three weight losses. The first weight loss (mass change: 5.7%) below 220 °C contributed to volatilization of the physically adsorbed water and organic solvent. The second step contains 17% weight loss between 220-600 °C and for the third step 5.6% weight loss between 590–800 °C observed. These results indicated that HMS/Pr-PTSC-Cu is stable to 220 °C (only 5.7% weight loss in this temperature range). In addition, about 17% weight loss observed between 220-600 °C attributed to decomposition of groups that attached in the surface of pores of support that confirmed successful synthesis of the catalyst.

In order to define the exact amount of Cu loaded on functionalized HMS in synthesized catalyst, atomic adsorption spectroscopy (AAS) was performed whereupon, the exact loading of Cu in the HMS/Pr-PTSC-Cu was obtained 0.63 mmol/g.

**Figure 13:**
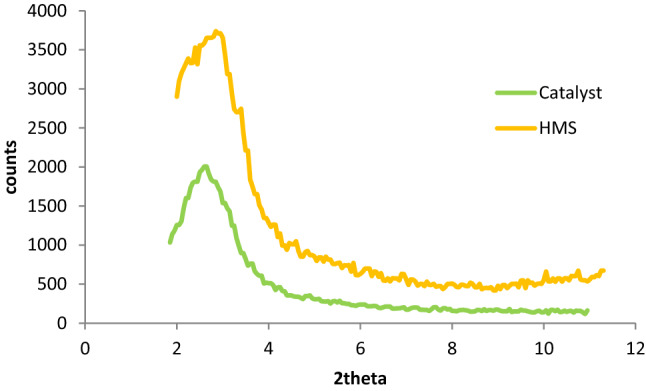
XRD pattern of HMS and HMS/Pr-PTSC-Cu.

The crystallinity of HMS and synthesized mesoporous catalyst (HMS/Pr-PTSC-Cu) was observed by low angle XRD pattern (Fig. 13). Patterns display one sharp reflection. This comparison explains and confirms this fact that, there is no changes in the phase of HMS during functionalization process^4^.

**Figure 14:**
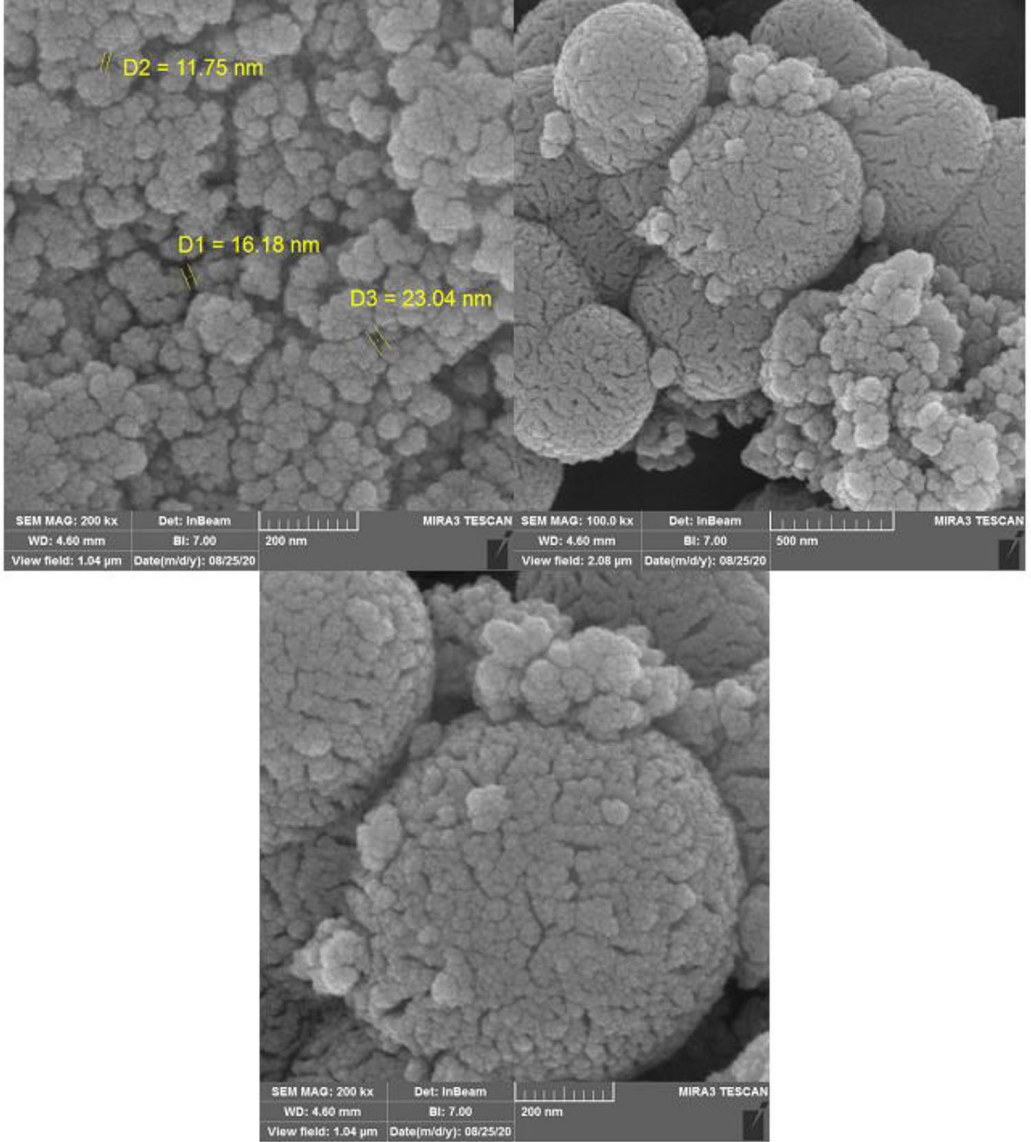
SEM images of HMS/Pr-PTSC-Cu.

**Figure 15:**
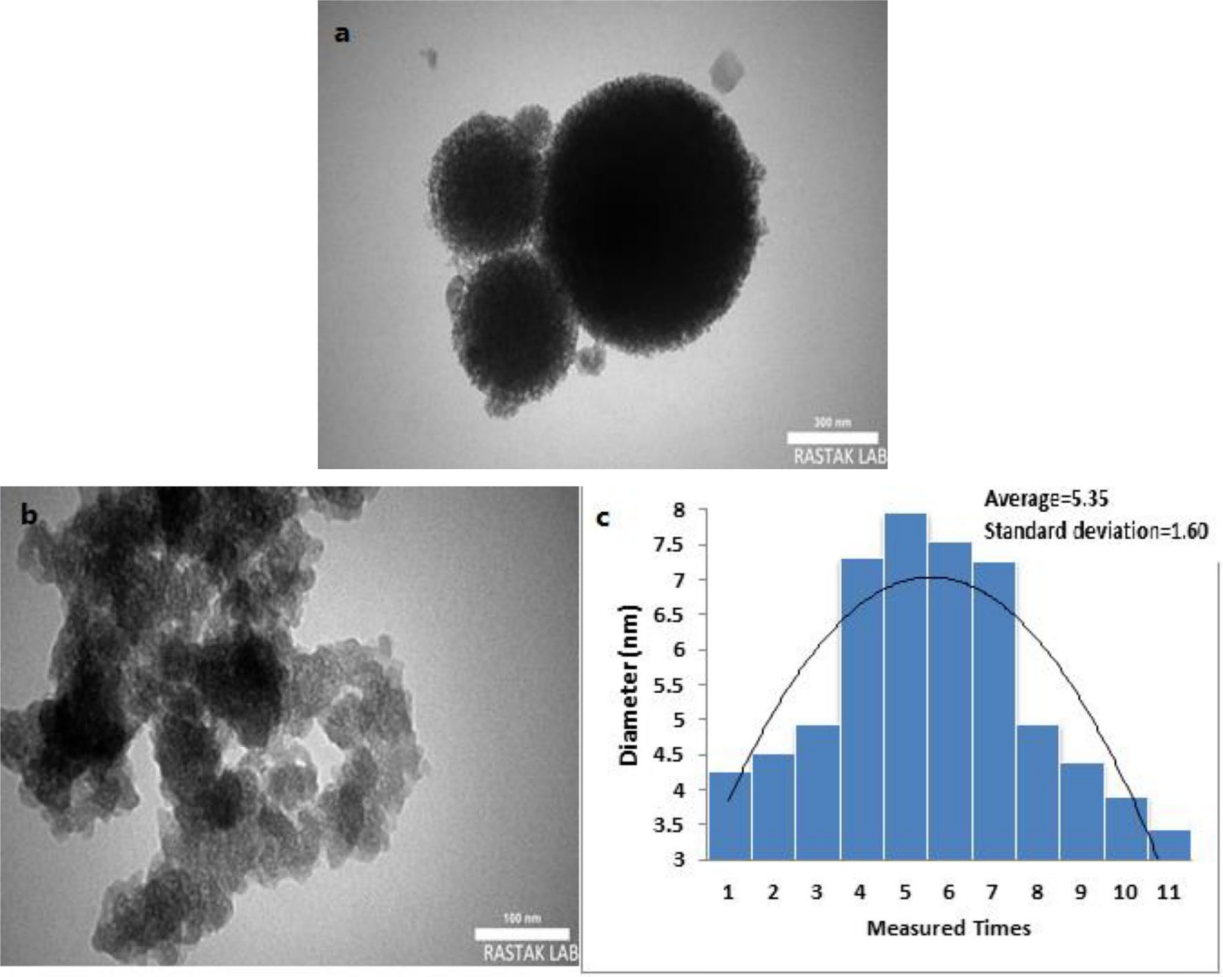
TEM images of HMS/Pr-PTSC-Cu (**a,b**), particle size distribution histogram of HMS/Pr-PTSC-Cu (**c**).

Figure 14 presents the SEM images of HMS/Pr-PTSC-Cu. SEM analysis was applied to determine its morphology and size distribution. It can be seen from SEM images that HMS/Pr-PTSC-Cu possessed regular and ordered structure with particle sizes of less than 23 nm. TEM images of HMS/Pr-PTSC-Cu (a, b) and particle size distribution histogram of HMS/Pr-PTSC-Cu (c) indicated in Fig. 15. The obtained result from this analysis indicated that the average size of the particle of catalyst B is 5.35 nm with 1.60 nm standard deviation.

**Figure 16:**
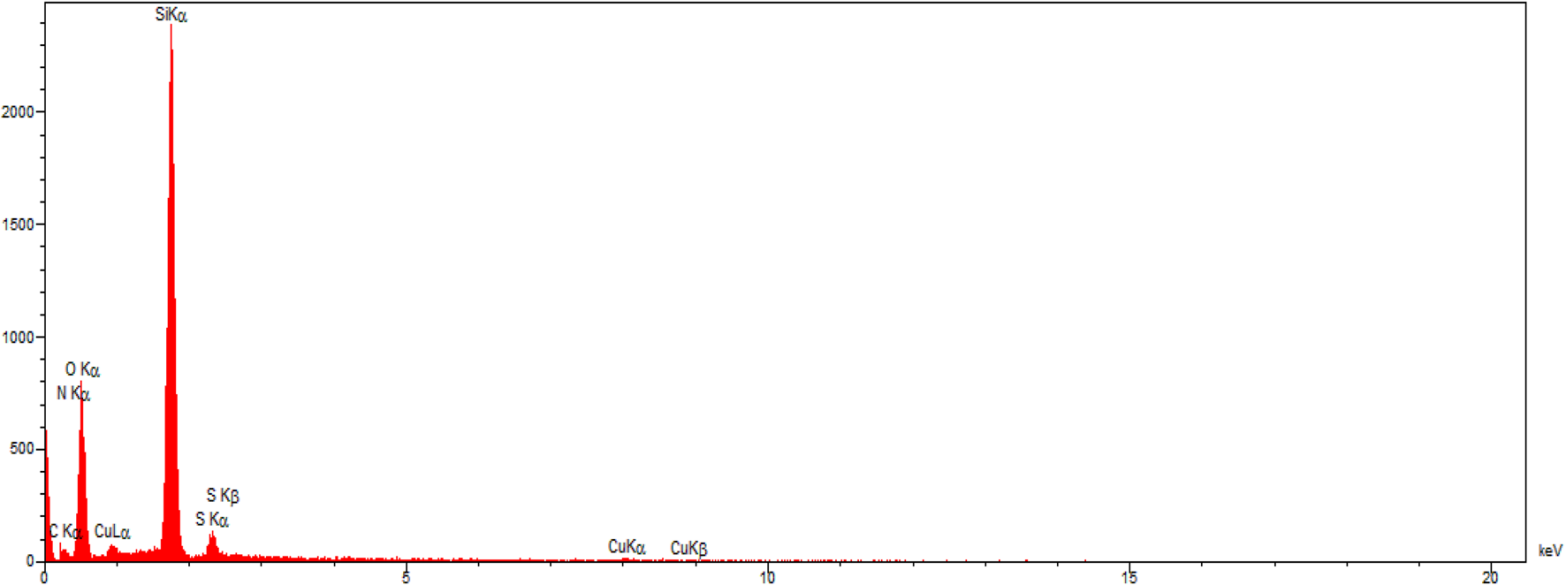
EDX spectrum of HMS/Pr-PTSC-Cu.

The EDX spectrum of HMS/Pr-PTSC-Cu illustrated in Fig. 16. As indicated by the EDX result, the expected elemental constituent was obtained for synthesised catalyst (C, N, O, Si, S, Cu). The EDX spectrum provides further evidence of successful immobilization of Pr-PTSC-Cu in pore of HMS.

**Figure 17:**
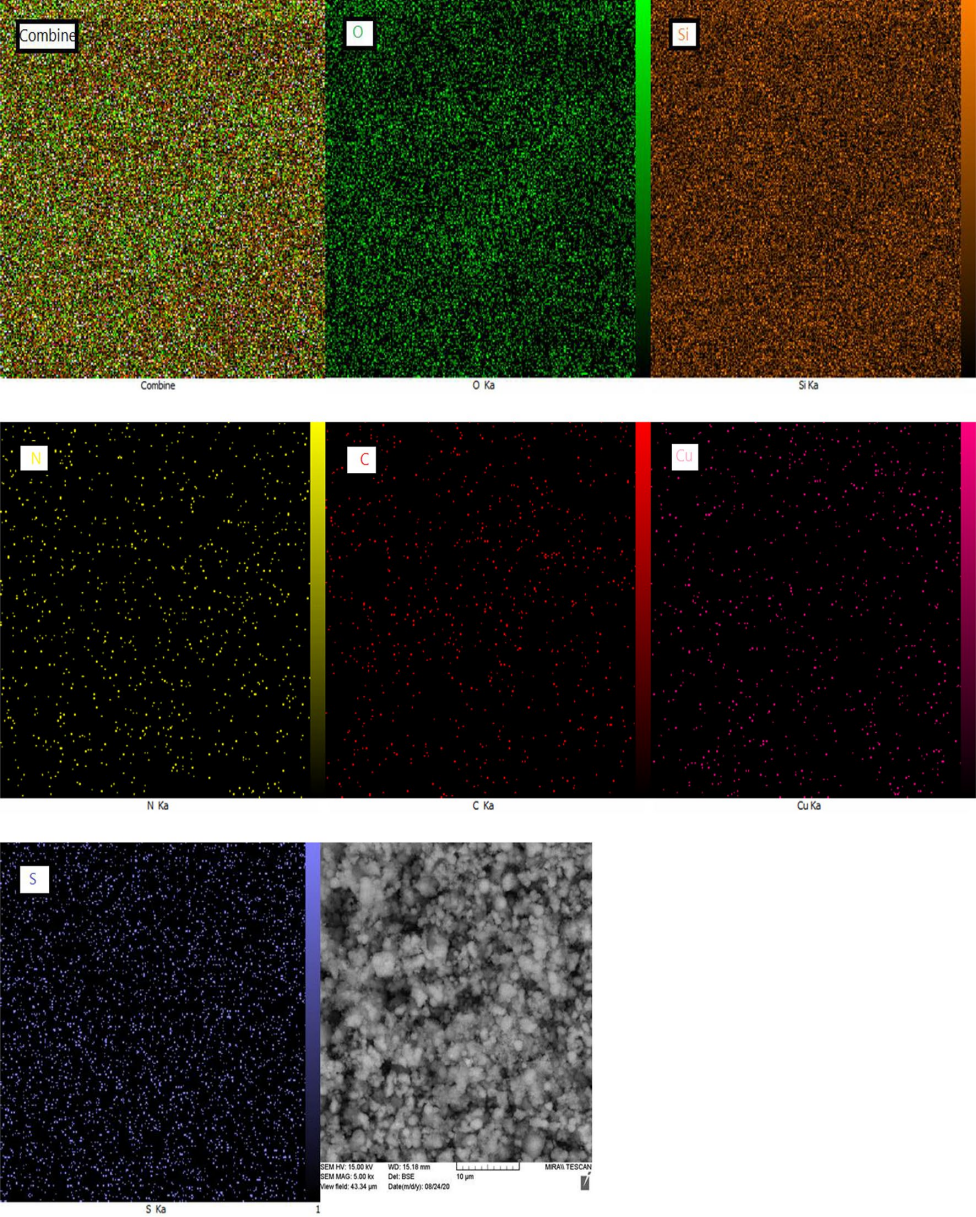
The elements mapping analysis of HMS/Pr-PTSC-Cu.

Also, for specification of dispersion of all elements of the synthesised catalyst, the map analysis was applied that as illustrated in Fig. 17, the elemental map images confirmed the good dispersion of C, N, O, Si, S and Cu in HMS/Pr-PTSC-Cu.

**Figure 18:**
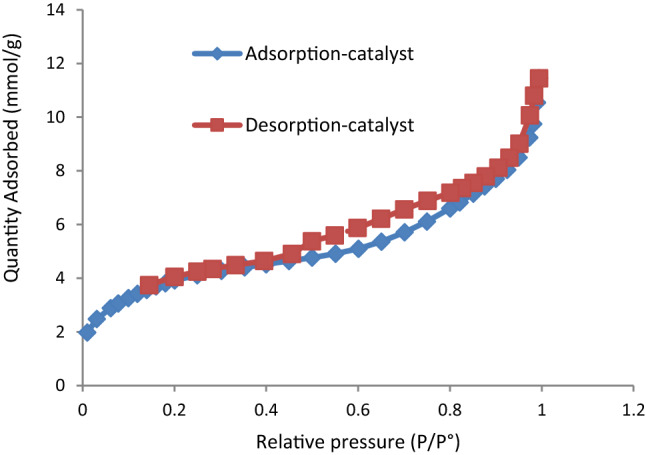
Nitrogen adsorption–desorption isotherm of HMS/Pr-PTSC-Cu.

A textural property of HMS/Pr-PTSC-Cu was obtained by nitrogen adsorption/desorption analysis and indicated in Table 2. Based on this analysis, BET surface area (S_BET_), total pore volumes (V_total_) and pore diameters (D_BJH_) of prepared catalyst were determined. Also, N_2_ adsorption-desorption isotherm showed in Fig. 18.

**Table 2:** Textural properties of HMS/Pr-PTSC-Cu obtained by nitrogen adsorption/desorption analysis.

Sample name	S_BET_ (m^2^ g^−1^)	D_BJH_ (nm)	V_Total_ (cm^3^ g^−1^)
HMS/Pr-PTSC-Cu	325	3.9	**0.32**

### Investigation of the catalytic activity of HMS/Pr-Xa-Ni for synthesis of tetrahydrobenzo[b]pyran and 1,4-dihydropyrano[2,3-c]pyrazole

After the identification and confirmation of catalysts, we decided to investigation the catalytic activity of HMS/Pr-Xa-Ni for synthesis of tetrahydrobenzo[b]pyran and 1,4-dihydropyrano[2,3-c]pyrazole.

In order to optimize the reaction conditions, many reactions were undertaken wherein the amount of catalyst, various solvents and different temperature were checked (Table 3). In this light the reaction of 4-chlorobenzaldehyde (1 mmol), malononitrile (1 mmol), dimedone (1 mmol) and HMS/Pr-Xa-Ni as catalyst was selected as model reaction. After evaluation of effect of mentioned factors on the model reaction, the results were revealed that 0.04 g of HMS/Pr-Xa-Ni in H_2_O: EtOH (3:1 mL) at 80 °C was found to be ideal reaction condition for the synthesis of tetrahydrobenzo[b]pyran. For investigation of necessity of presence of nickel in promote of the reaction, the reaction was under taken in presence of HMS/Pr-Xa. The yield of this reaction was 30% (Table 3, entry 12).

**Table 3:** Optimization for the synthesis of tetrahydrobenzo[b]pyran with 4-chlorobenzaldehyde, malononitrile, dimedone and HMS/Pr-Xa-Ni as catalyst^a^.

Entry	Catalyst (g)	Solvent	Temperature (°C)	Yield %^b^
1	0	H_2_O: EtOH^c^	80	33
2	0.03	H_2_O: EtOH^c^	80	48
**3**	**0.04**	**H**_**2**_**O: EtOH**^**c**^	**80**	**91**
4	0.05	H_2_O: EtOH^c^	80	90
5	0.06	H_2_O: EtOH^c^	80	91
6	0.04	EtOH	reflux	48
7	0.04	H_2_O	80	37
8	0.04	PEG	80	67
9	0.04	Solvent Free	80	43
10	0.04	H_2_O: EtOH ^c^	60	38
11	0.04	H_2_O: EtOH ^c^	100	91^d^
12	0.04^e^	H_2_O: EtOH ^c^	80	30

^a^4-Chlorobenzaldehyde (1 mmol), malononitrile (1 mmol), dimedone (1 mmol), 45 min.

^b^Pure yield.

^c^Ratio: 3:1 mL.

^d^40 min.

^e^The reaction catalyzed by HMS/Pr-Xa.

Significant values are in bold.

**Table 4:** One-pot synthesis of tetrahydrobenzo[b]pyran with aldehyde, malononitrile and dimedone catalyzed by HMS/Pr-Xa-Ni^a^.

Entry	Product	Time (min)	Yield (%)^b^	TOF (h^−1^)	M.p (°C)	Ref
1		40	91	377	210	^28^
2		50	87	291	198–201	^29^
3		35	97	465	177–179	^30^
4		40	98	406	208–210	^31^
5		35	75	359	225–228	^32^
6		240	73	51	196–198	^24^
7		180	72	67	210–212	^29^
8		30	91	506	121–123	^33^
9		45	83	307	227–228	^28^
10		60	69	192	205–207	^34^
11		45	80	296	218–220	^25^

^a^Aldehyde (1 mmol), malononitrile (1 mmol), dimedone (1 mmol), HMS/Pr-Xa-Ni (0.04 g) in H_2_O: EtOH (3:1 mL) at 80 °C.

^b^Pure yield.

**Figure 19:**
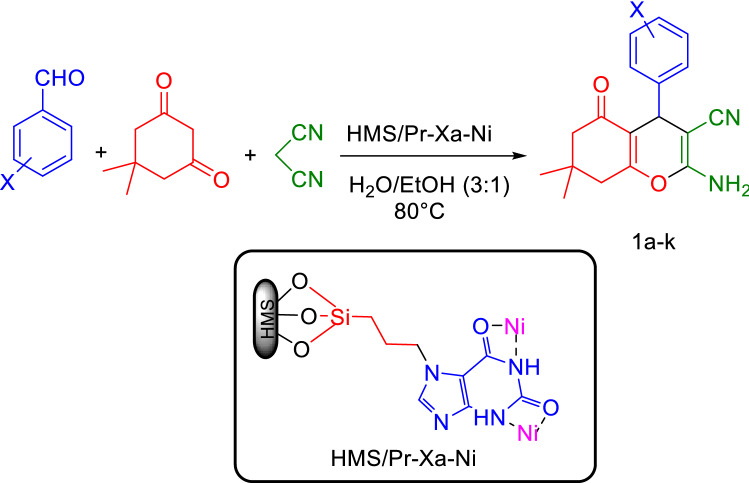
General procedure for the synthesis of tetrahydrobenzo[b]pyran.

In order to explore the scope and the limitations of this novel and heterogenous catalytic system, for the synthesis of tetrahydrobenzo[b]pyran (1a-k), we evaluated the reaction using wide range of electron-withdrawing and electron-donating substituted aldehyde (Fig. 19). The results are summarized in Table 4.

**Figure 20:**
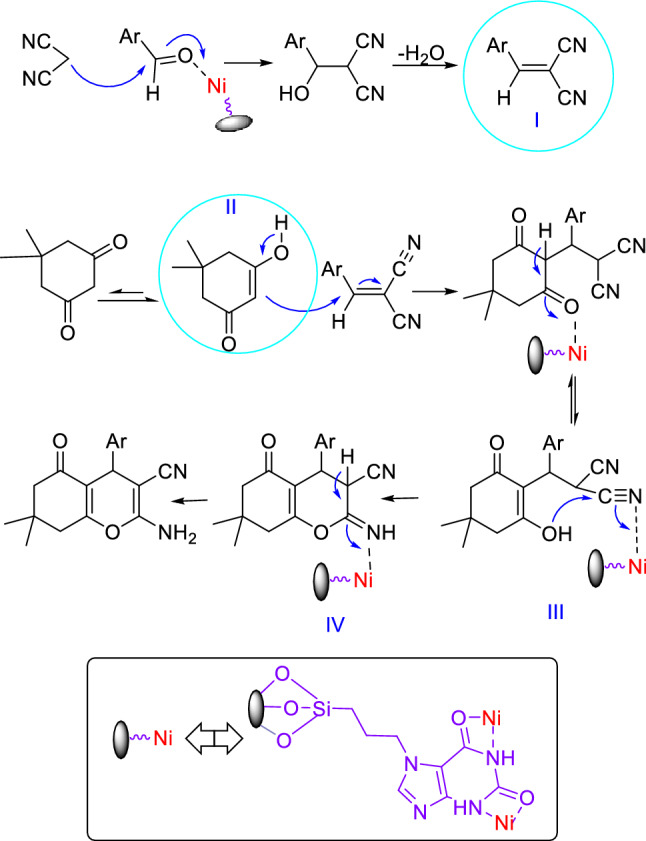
The possible mechanism for the synthesis of tetrahydrobenzo[b]pyran.

The suggested mechanism for the synthesis of tetrahydrobenzo[b]pyran in the presence of HMS/Pr-Xa-Ni is indicated in Fig. 20. The condensations of malononitrile with activated aldehyde afford arylidenemalononitrile intermediate (I). Then the enolized dimedone reacts with the arylidenemalononitrile intermediate (I) by the nucleophilic addition. Finally, intramolecular cyclization occurs and ultimately the expected tetrahydrobenzo[b]pyran produces with rearrangement^11^.

In the second part for determination of the optimal reaction conditions, for synthesis of 1,4-dihydropyrano[2,3-c]pyrazole, the reaction of 4-chlorobenzaldehyde (1 mmol), malononitrile (1 mmol), ethyl acetoacetate (1 mmol) and hydrazine hydrate (1 mmol) in the presence of HMS/Pr-Xa-Ni as catalyst was undertaken. For this purpose, various effective factors on the model reaction such as: amount of catalyst (catalyst free, 0.008, 0.01 and 0.02 g), solvents (H_2_O, EtOH, PEG, H_2_O: EtOH and solvent free) and also the effect of temperature (35, 80 and 100 °C) were checked. As screened in Table 5, the obtained results were demonstrated that 0.01 g of catalyst, mixture of H_2_O: EtOH at 35 °C were the most effective condition. For indicating necessity of presence of nickel in catalytic activity, the model reaction investigated in presence of HMS/Pr-Xa. That result indicated that yield of this reaction was obtained 28% (Table 5, entry 12).

**Table 5:** Optimization of reaction condition for synthesis of 1,4-dihydropyrano[2,3-c]pyrazole under different conditions^a^.

Entry	Catalyst (g)	Solvent	Temperature (°C)	Yield %^b^
1	0	H_2_O: EtOH^c^	35	25
2	0.008	H_2_O: EtOH^c^	35	78
**3**	**0.01**	**H**_**2**_**O: EtOH**^**c**^	**35**	**90**
4	0.02	H_2_O: EtOH^c^	35	91
5	0.01	H_2_O: EtOH^d^	35	84
6	0.01	H_2_O: EtOH^c^	80	92
7	0.01	H_2_O: EtOH^c^	100	92
8	0.01	H_2_O	35	58
9	0.01	PEG	35	41
10	0.01	Solvent Free	35	30
11	0.01	EtOH	35	60
12	0.01^e^	H_2_O: EtOH ^c^	35	28

^a^4-Chlorobenzaldehyde (1 mmol), malononitrile (1 mmol), ethyl acetoacetate (1 mmol), hydrazine hydrate (1 mmol), 20 min.

^b^Pure yield.

^c^Ratio: 2:1 mL.

^d^Ratio: 1:1 mL.

^e^The reaction catalyzed by HMS/Pr-Xa.

Significant values are in bold.

**Figure 21:**
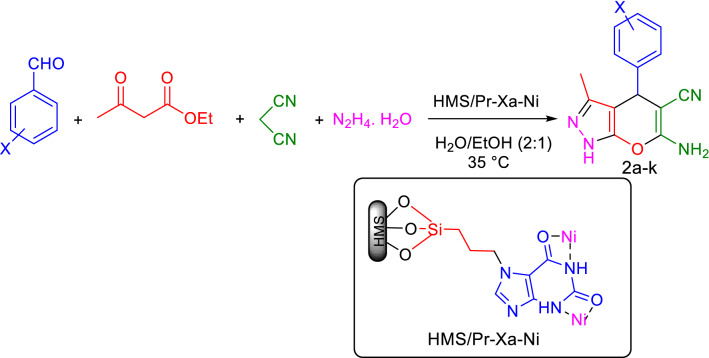
General procedure for the synthesis of 1,4-dihydropyrano[2,3-c]pyrazole.

After optimization of the reaction conditions, as shown in Table 6, 1,4-dihydropyrano[2,3-c]pyrazole derivatives (2a-k) were synthesized by using various electron-withdrawing and electron-donating substituted aldehydes (Fig. 21).

**Table 6:** One-pot synthesis of 1,4-dihydropyrano[2,3-c]pyrazole with aldehyde, malononitrile, ethyl acetoacetate and hydrazine hydrate catalyzed by HMS/Pr-Xa-Ni^a^.

Entry	Product	Time (min)	Yield (%)^b^	TOF (h^-1^)	M.p (°C)	Ref
1		20	90	3030	232–233	^35^
2		15	94	4177	224–226	^14^
3		20	85	2861	235–238	^36^
4		10	90	5882	237–244	^37^
5		15	88	3911	195–200	^38^
6		20	78	2626	202–203	^36^
7		25	91	2407	237–242	^39^
8		35	83	1590	209–211	^38^
9		90	80	592	218–222	^40^
10		60	93	1033	227–228	^37^
11		90	83	614	234	^41^

^a^Aldehyde (1 mmol), malononitrile (1 mmol), ethyl acetoacetate (1 mmol), hydrazine hydrate (1 mmol), HMS/Pr-Xa-Ni ( 0.01 g) in H_2_O: EtOH (2:1 mL) at 35 °C.

^b^Pure yield.

**Figure 22:**
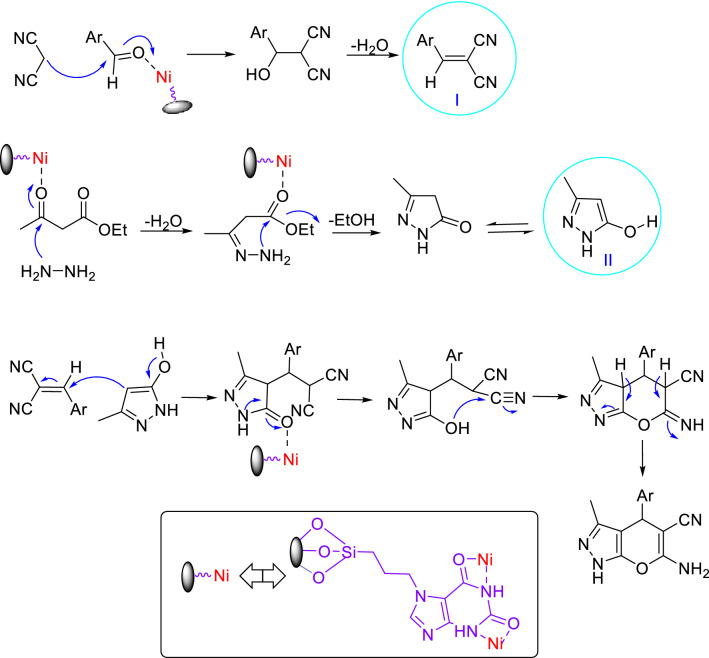
The possible mechanism for the synthesis of 1,4-dihydropyrano[2,3-c]pyrazole.

A possible mechanism for the synthesis of 1,4-dihydropyrano[2,3-c]pyrazole using HMS/Pr-Xa-Ni as catalyst is demonstrated in Fig. 22. The Knoevenagel condensation of malononitrile and aldehyde (activated by Ni of the catalyst), leads to obtain the intermediate of arylidenemalononitrile (intermediate I). Also, pyrazolone (intermediate II) is produced by the condensation of hydrazine and ethyl acetoacetate (activated by Ni of the catalyst). In the final step, the Michael addition of the enolized pyrazolone to the arylidenmalononitrile and subsequently tautomerization of the intermediate leads to form corresponding product^11^.

### Investigation of the catalytic activity of HMS/Pr-PTSC-Cu for synthesis of tetrahydrobenzo[b]pyran and 1,4-dihydropyrano[2,3-c]pyrazole

After successful characterization, the catalytic application of HMS/Pr-PTSC-Cu was evaluated for synthesis of tetrahydrobenzo[b]pyran and 1,4 dihydropyrano[2,3-c]pyrazole.

In the first, to study of the catalytic activity of HMS/Pr-PTSC-Cu for synthesis of tetrahydrobenzo[b]pyran, the reaction of 4-chlorobenzaldehyde (1 mmol), malononitrile (1 mmol), dimedone (1 mmol) and HMS/Pr-PTSC-Cu as catalyst was choosed as model reaction.

To define the optimal condition of reaction, various amount of catalyst (0.02 g, 0.015 g, 0.01 g, 0.008 g, 0.006 g, 0.004 g, 0.002 g and catalyst free condition), different solvent (EtOH, H_2_O, PEG, H_2_O:EtOH and solvent free condition) and temperatures of 45 °C, 60 °C and room temperature were examined. Comparison of obtained results revealed that the best efficiencies were obtained in the presence of 0.004 g of catalyst in ethanol at room temperature (Table 7, entry 7).

**Table 7:** Optimization of reaction condition for the synthesis of tetrahydrobenzo[b]pyran^a^.

Entry	Catalyst (g)	Solvent	Temperature (°C)	Yield%^b^
1	0	EtOH	r.t	41
2	0.02	EtOH	r.t	88
3	0.015	EtOH	r.t	89
4	0.01	EtOH	r.t	91
5	0.008	EtOH	r.t	93
6	0.006	EtOH	r.t	95
**7**	**0.004**	**EtOH**	**r.t**	**97**
8	0.002	EtOH	r.t	90
9	0.004	EtOH	45	93
10	0.004	EtOH	60	87
11	0.004	Solvent-free	r.t	78
12	0.004	H_2_O	r.t	83
13	0.004	H_2_O:EtOH^c^	r.t	85
14	0.004	PEG	r.t	80
15	0.004^d^	EtOH	r.t	3

^a^4-Chlorobenzaldehyde (1 mmol), malononitrile (1 mmol), dimedone (1 mmol), 30 min.

^b^Pure yield.

^c^Ratio: 2:1 mL.

^d^The reaction catalyzed by HMS/Pr-PTSC.

Significant values are in bold.

To investigate the effectiveness of the presented catalytic system, the model reaction was carried out in the absence of catalyst (Table 7, entry 1) and in the presence of HMS/Pr-PTSC (lack of Cu) (Table 7, entry 15). The yields for these reactions were obtained 41% and 38% respectively.

**Table 8:** One-pot synthesis of tetrahydrobenzo[b]pyran with aldehyde, malononitrile and dimedone catalyzed by HMS/Pr-PTSC-Cu^a^.

Entry	Product	Time (min)	Yield (%)^b^	TOF (h^-1^)	M.p (°C)	Ref
1		30	97	7698	209–211	^25^
2		35	96	6568	198–200	^42^
3		70	81	2747	176–178	^43^
4		40	93	5508	210–212	^44^
5		30	96	7619	228–231	^19^
6		80	80	2386	194–196	^45^
7		65	83	3049	210–212	^45^
8		40	95	5626	226–230	^46^
9		50	88	4207	205–207	^47^
10		60	85	3373	230–234	^43^
11^c^		15	99	15,714	256–260	^48^

^a^Reaction conditions: aldehyde (1 mmol), malononitrile (1 mmol), dimedone (1 mmol), HMS/Pr-PTSC-Cu (0.004 g) in EtOH at room temperature.

^b^Pure yield.

^c^Reaction conditions: aldehyde (1 mmol), malononitrile (2 mmol), dimedone (2 mmol), HMS/Pr-PTSC-Cu (0.008 g) in EtOH at room temperature.

**Figure 23:**
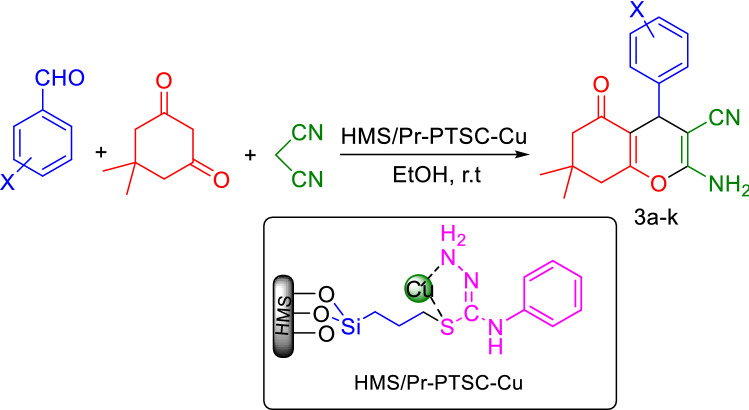
General procedure for the synthesis of tetrahydrobenzo[b]pyran.

To specify the extent of the reaction we reacted various of substituted of aldehydes under optimized conditions to produce tetrahydrobenzo[b] pyran derivatives (3a-k) (Fig. 23, Table 8).

According to the results it was found that electron-withdrawing groups (such as nitro and halides) rather than electron-donating groups (such as methoxy, methyl and hydroxy) on aldehyde provide better results from the viewpoint of time and yield.

In this part of work, for determination of the utility scope of our catalyst in organic reactions, the reaction of aldehyde (1 mmol) with malononitrile (1 mmol), ethyl acetoacetate (1 mmol), hydrazine hydrate (1 mmol) in presence of HMS/Pr-PTSC-Cu as catalyst was designed for synthesis of 1,4-dihydropyrano[2,3-c]pyrazole derivatives.

For optimization of the conditions, catalyst dosing (0.02 g, 0.015 g, 0.01 g, 0.008 g, 0.006 g, 0.004 g and catalyst free condition), type of solvent (H_2_O:EtOH, H_2_O, PEG, EtOH and solvent free condition) and temperature (room temperature, 45 °C and 60 °C) were studied. With evaluation of these parameters, we found that in the presence of 0.006 g of HMS/Pr-PTSC-Cu as catalyst in H_2_O:EtOH at room temperature the best conversion is achieved (Table 9, entry 6).

**Table 9:** Optimization of reaction condition for synthesis of 1,4-dihydropyrano[2,3-c]pyrazole^a^.

Entry	Catalyst (g)	Solvent	Temperature (°C)	Yield%^b^
1	0	H_2_O: EtOH^c^	r.t	45
2	0.02	H_2_O: EtOH^c^	r.t	85
3	0.015	H_2_O: EtOH^c^	r.t	88
4	0.01	H_2_O: EtOH^c^	r.t	90
5	0.008	H_2_O: EtOH^c^	r.t	94
**6**	**0.006**	**H**_**2**_**O: EtOH**^**c**^	**r.t**	**98**
7	0.004	H_2_O: EtOH^c^	r.t	92
8	0.006	H_2_O: EtOH^c^	45	91
9	0.006	H_2_O: EtOH^c^	60	85
10	0.006	H_2_O: EtOH^d^	r.t	88
11	0.006	H_2_O	r.t	87
12	0.006	PEG	r.t	78
13	0.006	Solvent Free	r.t	72
14	0.006	EtOH	r.t	86
15	0.006^e^	H_2_O: EtOH^c^	r.t	40

^a^4-Chlorobenzaldehyde (1 mmol), malononitrile (1 mmol), ethyl acetoacetate (1 mmol), hydrazine hydrate (1 mmol), 20 min.

^b^Pure yield.

^c^Ratio: 2:1 mL.

^d^Ratio: 1:1 mL.

^e^Reaction catalysed by HMS/Pr-PTSC.

Significant values are in bold.

In another study, the reaction was performed in the presence of HMS/Pr-PTSC to investigate the effect of copper in the catalyse of reaction (Table 9, entry 15). The obtained yield (40%) showed that the presence of Cu was necessaries for progress of reaction.

**Figure 24:**
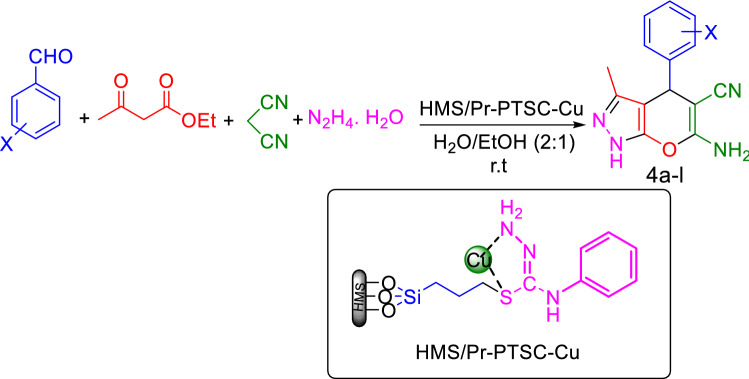
General procedure for the synthesis of 1,4-dihydropyrano[2,3-c]pyrazole.

After optimization of the reaction conditions, benzaldehyde, electron-donating containing aldehydes as well as electron-withdrawing bearing aldehydes were applied as substrate and 1,4-dihydropyrano[2,3-c]pyrazole derivatives (4a-l) were obtained in high to excellent yield as summarized in Table 10 (Fig. 24). Assessment of results indicated that electron-withdrawing bearing aldehydes have higher yield and shorter reaction time.

**Table 10:** One-pot synthesis of 1,4-dihydropyrano[2,3-c]pyrazole with aldehyde, malononitrile, ethyl acetoacetate and hydrazine hydrate catalyzed by HMS/Pr-PTSC-Cu^a^.

Entry	Product	Time (min)	Yield (%)^b^	TOF (h^-1^)	M.p (°C)	Ref
1		20	98	7856	230–232	^35^
2		20	98	7856	235–237	^36^
3		20	97	7776	234–236	^41^
4		25	95	5983	219–221	^49^
5		25	92	5794	243–245	^50^
6		40	90	3553	207–209	^51^
7		50	84	2677	224–226	^52^
8		65	80	1959	200–204	^53^
9		55	82	2357	232–234	^41^
10		65	83	2033	162–165	^54^
11		90	85	1499	205–208	^50^
12^c^		15	92	9735	> 300	–

^a^Reaction conditions: aldehyde (1 mmol), malononitrile (1 mmol), ethyl acetoacetate (1 mmol), hydrazine hydrate (1 mmol), HMS/Pr-PTSC-Cu (0.006 g) in H_2_O: EtOH (2:1 mL) at room temperature.

^b^Pure yield.

^c^Reaction conditions: aldehyde (1 mmol), malononitrile (2 mmol), ethyl acetoacetate (2 mmol), hydrazine hydrate (2 mmol), HMS/Pr-PTSC-Cu ( 0.012 g) in H_2_O: EtOH (2:1 mL) at room temperature.

### Comparison of efficiency of catalysts

In this work, two heterogeneous HMS supported metal catalysts were screened in the synthesis of tetrahydrobenzo[b]pyran and 1,4-dihydropyrano[2,3-c]pyrazole derivatives. Turnover frequency (TOF) value is an important parameter to evaluate the efficiency of the catalyst, which quantifies how many catalytic reaction cycles proceed per site and per unit of time^4^. TOF was measured for all products and is given in the Tables 4,6,8 and 10. Based on the results achieved from ICP and AAS, Ni and Cu histograms at TEM and turnover frequency (TOF) obtained from yield, time and amount of catalyst (mol% of Cu and Ni), the investigated metals can be ranked as copper metal has performed better. Judging by the contrast of TEM images, the size distribution of copper particles is better visible.

### Recyclability of the catalysts

#### Reusability and recycling of the HMS/Pr-Xa-Ni

**Figure 25:**
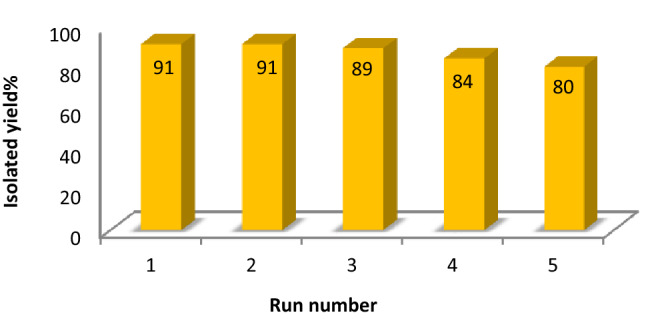
Recyclability of HMS/Pr-Xa-Ni in the synthesis of 2-amino–4-(4-chlorophenyl)-3–cyano-7,7–dimethyl-5-oxo-4H-5,6,7,8-tetrahydro-benzo[b]pyran.

Reusability of the catalysts is one of the most crucial aspects of organic synthesis. In this respect, reusability of HMS/Pr-Xa-Ni was investigated for synthesis of 2-amino–4-(4-chlorophenyl)-3–cyano-7,7–dimethyl-5-oxo-4H-5,6,7,8-tetrahydro benzo[b]pyran. After completion of the reaction, hot EtOH was added to the crude mixture and centrifuged several times. The separated catalyst was washed, dried for overnight and applied for the next run. The HMS/Pr-Xa-Ni was found to be reusable for five successive runs with a negligible decrease in its activity (Fig. 25).

#### Reusability and recycling of the HMS/Pr-PTSC-Cu

**Figure 26:**
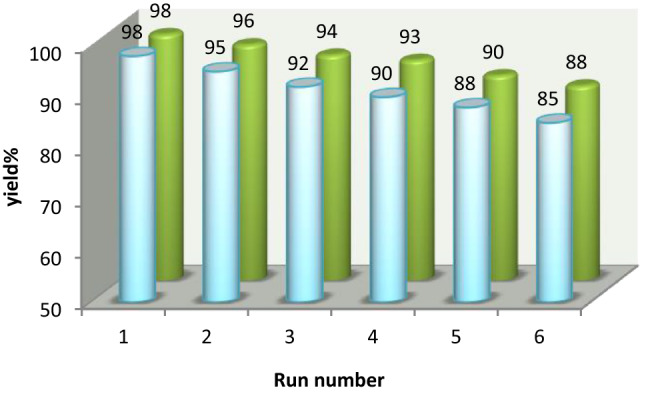
Recyclability of HMS/Pr-PTSC-Cu in the synthesis of tetrahydrobenzo[b]pyran (blue) and 1,4-dihydropyrano[2,3-c]pyrazole (green).

Since the recycling of catalyst is of great importance in the industry, the recyclability of HMS/Pr-PTSC-Cu in the synthesis of 2–amino–3–cyano–7,7–dimethyl–4-(4-chlorophenyl)–5–oxo-4H–5,6,7,8-tetrahydro benzopyran and 6-amino-4-(4-chlorophenyl)-3-methyl-1,4-dihydropyrano[2,3-c]pyrazole-5-carbonitrile was studied. After completion of the reaction, the catalyst was separated by centrifuge instrument and dried. Then, dried separated catalyst was reused in the same reaction for six successive runs with minimal decrease in the yield of product (Fig. 26).

### Characterization of recycled catalyst of HMS/Pr-PTSC-Cu

In order to examine the stability of HMS/Pr-PTSC-Cu after recovering and reusing, recovered catalyst was characterized by FT-IR and AAS techniques.

**Figure 27:**
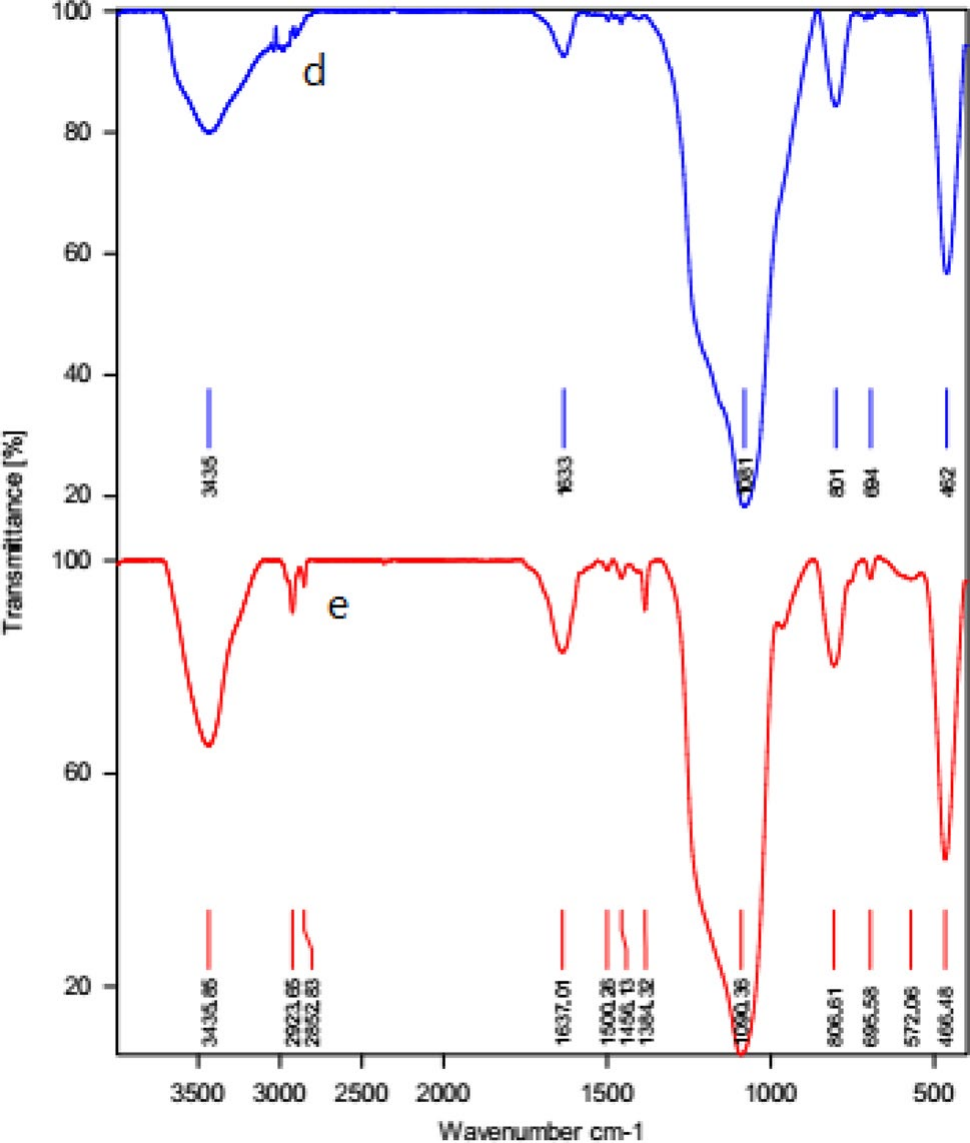
The FT-IR spectrum of HMS/Pr-PTSC-Cu (d) and recovered HMS/Pr-PTSC-Cu (e).

The FT-IR spectrum of the fresh catalyst and recovered catalyst are shown in Fig. 27. As shown, the symmetric and asymmetric stretching vibrations of Si–O–Si are appearing in 807 cm^−1^ and 1090 cm^−1^. Also, the peaks of 1384 cm^−1^ and 1637 cm^−1^ are contributed to (C=C) aromatic ring. Also, the vibration of NH observed in 3436 cm^−1^. It can be seen that the structure of catalyst was preserved after recovery.

Also AAS analysis contributed to recovered HMS/Pr-PTSC-Cu was done that base on this analysis copper concentration in recovered catalyst was obtained 0.50 mmol.g^−1^. This analysis confirmed that copper leaching was low.

The hot filtration experiment was investigated to determine leaching of copper in the reaction mixture and to show that HMS/Pr-PTSC-Cu is a heterogeneous catalyst. For this regard the reaction between 4-chlorobenzaldehyde, malononitrile, ethyl acetoacetate, hydrazine hydrate, HMS/Pr-PTSC-Cu in H_2_O: EtOH (2:1 mL) at room temperature was choose. In this experiment the product was obtained in the half time of the reaction (10 min) in 66% yield. Then, the same reaction was repeated but in this reaction in the half time of the reaction (after 10 min), the catalyst was filtered from the reaction mixture and the reaction mixture was allowed to react for another 10 min. The yield of product in this experiment was 69%. The results from hot filtration test confirmed that leaching of copper during the reaction hasn’t been significant.

### Comparison results of synthesised catalysts with other catalysts

Comparison of the catalytic activity of our catalyst with those reported in the literature is useful in order to extend the scope of merit of synthesised catalyst. In this light, comparison of our studies with reported works was under taken for synthesis of 2-amino–4-(4-chlorophenyl)-3–cyano-7,7–dimethyl-5-oxo-4H-5,6,7,8-tetrahydro-benzo[b]pyran (Table 11, entry 1-7) and 6-Amino-4-(4-chlorophenyl)-3-methyl-2,4-dihydropyrano[2,3-c]pyrazole-5-carbonitrile (Table 11, entry 8-15). We find that our catalytic systems benefit mild reaction conditions, good to high yields and short reaction times.

**Table 11:** Comparison results of HMS/Pr-Xa-Ni and HMS/Pr-PTSC-Cu with other catalysts.

Entry	Conditions	Time (min)	Yield %	Ref
1	[Ch][OH], H_2_O, 80°C^a^	60	86	^2,4^
2	CaHPO_4_ (10wt%), H_2_O/EtOH (4:1), 80°C^a^	120	92	^32^
3	Fe_3_-xTixO_4_@SO_3_HNPs (0.03 g), EtOH (3 mL) /H_2_O (3 mL), reflux^a^	60	95	^55^
4	SiO_2_-Pr-SO_3_H, H_2_O, reflux^a^	20	90	^34^
5	Fe_3_O_4_@SiO_2_/DABCO (0.05 g), H_2_O , 80 °C ^a^	25	90	^56^
6	HMS/Pr-Xa-Ni ( 0.04 g), H_2_O: EtOH (3:1 mL), 80°C^a^	40	91	This work
7	HMS/Pr-PTSC-Cu (0.004 g), EtOH, r.t^a^	30	97	This work
8	β-cyclodextrin (10 mol %), H_2_O-EtOH (9:1), 80°C^b^	15	92	^38^
9	BSA (30 mg), H_2_O: EtOH (7:3 mL), r.t^b^	120	88	^40^
10	ɣ-alumina (30 mol%), H_2_O, reflux^b^	35	90	^57^
11	Ag/TiO_2_ nano-thin films, H_2_O: EtOH (1:2 mL), 70°C^b^	25	93	^36^
12	Sodium gluconate (10 mol%), H_2_O, reflux^b^	20	91	^7^
13	Urea (10 mol %) H_2_O: EtOH (1:1 v/v), r. t^b^	12 h	84	^58^
14	HMS/Pr-Xa-Ni ( 0.01 g), H_2_O: EtOH (2:1 mL), 35°C^b^	20	90	This work
15	HMS/Pr-PTSC-Cu ( 0.006 g), H_2_O: EtOH (2:1 mL), r. t^b^	20	98	This work

^a^Reaction of 4-chlorobenzaldehyde, dimedone and malononitrile for the synthesis of 2–amino–3–cyano–7,7–dimethyl–4-(4-chlorophenyl)–5–oxo-4H–5,6,7,8-tetrahydro benzopyran.

^b^Reaction of 4-chlorobenzaldehyde, malononitrile, ethyl acetoacetate and hydrazine hydrate for the synthesis of 6-amino-4-(4-chlorophenyl)-3-methyl-1,4-dihydropyrano[2,3-c]pyrazole-5-carbonitril.

## Experimental

### Materials and physical measurements

All reagents and solvents were provided from Aldrich and Merck chemical companies. ^1^H-NMR and ^13^C-NMR spectra of the DMSO-d_6_ solutions were determined at 300 MHz. The FT-IR spectra were recorded as KBr pellets by FT-IR, VERTEX 70, Bruker, Germany spectroscopy. The analysis of X-ray diffraction was conducted using a XRD, X’Pert PRO MPD, PANalytical, Netherland. Thermogravimetric analysis (TGA) was performed from room temperature to 800 °C by TGA, PerkinElmer Pyris Diamond, U.K. Scanning electron microscopy (SEM) images were carried out on a FE-SEM, TESCAN MIRA III, Czech. Transmission electron microscopy (TEM) were carried out on TEM Philips EM 208S. Elemental analysis was recorded with instruments EDX-MAP, FE-SEM, TESCAN MIRA, SAMX, Czech. The content of Ni was investigated using inductively coupled plasma optical emission spectrometry (ICP-OES, Arcos EOP, company of Spectro, Germany). The content of Cu was measured by atomic adsorption spectroscopy (AAS, Analytikjena- Nov AA 400/ Germany). The instruments of adsorption-desorption of nitrogen that was applied have this character: BET, Micromeritics, Asap2020, USA.

### Synthesis of catalysts (HMS/Pr-Xa-Ni and HMS/Pr-PTSC-Cu)

#### Synthesis of HMS

HMS was synthesized similar to a previously reported research. In this light, 5 gr of dodecylamine was dissolved in 70% w/w ethanol aqueous solution. Then 20.8 gof tetraethyl orthosilicate (TEOS) was added dropwise, stirred for 5 h at room temperature under vigorous stirring and aged for 18 h at room temperature. The resultant precipitate was filtered and dried at room temperature. Finally, the dried powder was soxhelt extraction at 80 °Cfor 24 h. For the removing the template, synthesized support was calcined at 500 °C in air for 5 h^4^.

#### Synthesis of HMS/Pr

For functionalization of HMS, to a mixture of 0.5 g HMS in toluene, 1.5 mL 3‐chloropropyltrimethoxysilane was added dropwise and stirred under nitrogen atmosphere at the reflux condition for 24 h. Then the reaction mixture was filtered, washed with toluene and dried at room temperature to obtain HMS/Pr.

#### Synthesis of HMS/Pr-Xa

In this step, xanthine sodium (7 mmol) was reacted with HMS/Pr (3 g) in DMF under nitrogen atmosphere at 100 °C for 24 h. The resultant solid was washed with H_2_O and EtOH and dried at room temperature.

#### Synthesis of HMS/Pr-Xa-Ni

Finally, for metalation of HMS/Pr-Xa and synthesis of sightly catalyst, 1 g of HMS/Pr-Xa was dispersed in absolute ethanol and 2.5 mmol Ni(NO_3_)_2_.6H_2_O was added to the mixture under nitrogen atmosphere at the reflux condition for 48 h. The resultant solid was filtered, washed several times with EtOH and dried at room temperature to overnight to afford HMS/Pr-Xa-Ni.

#### Synthesis of HMS/Pr-PTSC

For the synthesis of HMS/Pr-PTSC, initially 1 g 4-phenylthiosemicarbazide and 2 mL Et_3_N was stirred in toluene for 1 h in room temperature. Then 1g HMS/Pr was added to reaction mixture and stirred under nitrogen atmosphere and reflux condition for 48 h. The resulting solid was separated by filtration and washed a number of times with CH_2_Cl_2_. Finally, the product (HMS/Pr-PTSC) was dried.

#### Synthesis of HMS/Pr-PTSC-Cu

In the final step of synthesis of catalyst, 1 g HMS/Pr-PTSC was dispersed in EtOH for 30 min. Then 2.5 mmol Cu(NO_3_)_2_.3H_2_O was added and stirred under nitrogen atmosphere and reflux condition for 24 h. After filtration, the separated solid washed with EtOH and dried at room temperature to overnight. Finally desired catalyst (HMS/Pr-PTSC-Cu) was prepared.

### Reaction of 3‐chloropropyltrimethoxysilane with 4-phenylthiosemicarbazide

In order to prove the reaction between linkage and ligand, a mixture of 4-phenylthiosemicarbazide (1 mmol), 3‐chloropropyltrimethoxysilane (1 mmol) and K_2_CO_3_ (2 mmol) in EtOH were stirred with a magnetic stirrer at reflux condition for 24 h. Then the obtained precipitation separated by filtration and washed with EtOH. Purification of product was done through recrystallization in EtOH. According to the results obtained from FT-IR, ^1^H and ^13^C NMR, the structure of product determined and spectra have entered in supporting information.

### Characterization data of 3-(trimethoxysilyl)propyl (Z)-phenylcarbamohydrazonothioate

IR (KBr, ν) 3416, 2924, 2857, 1627, 1506, 1417, 1130, 1033, 760, 690, 474 cm^−1^.

^1^H NMR (300 MHz, DMSO-d6): 0.68 (s, 2H), 1.71 (s, 2H), 3.11 (s, 2H), 3.33–3.61 (m, 9H), 7.43–7.56 (m, 5H), 8.31 (s, 1H), 8.84 (s, 1H) ppm.

^13^C NMR (300 MHz, DMSO-d6): 20.9, 25.7, 47.3, 67.9, 126.0, 129.0, 132.5, 143.8, 147.8 ppm.

### General procedure for the synthesis of tetrahydrobenzo[b]pyran by HMS/Pr-Xa-Ni

A test tube including aldehyde (1 mmol), dimedone (1 mmol), malononitrile (1 mmol) and HMS/Pr-Xa-Ni (0.04 g) were mixed in H_2_O: EtOH (3:1 mL) at 80 °C. Completion of the reaction was monitored by TLC. Then the hot EtOH was added to reaction mixture and the catalyst was separated by filtration. Purification of products was done with recrystallization in EtOH.

### General procedure for the synthesis of 1,4-dihydropyrano[2,3-c]pyrazole by HMS/Pr-Xa-Ni

0.01 g of HMS/Pr-Xa-Ni was added to a mixture of aldehyde (1 mmol), hydrazine hydrate (1 mmol), ethyl acetoacetate (1 mmol) and malononitrile (1 mmol) in H_2_O: EtOH (2:1 mL) at 35 °C. Completion of the reaction was checked by TLC, then the catalyst was separated with filtration and washed with hot EtOH. Recrystallization with EtOH was applied to afford the pure products.

### General procedure for the synthesis of tetrahydrobenzo[b]pyran by HMS/Pr-PTSC-Cu

A mixture of aldehyde (1 mmol), dimedone (1 mmol), malononitrile (1 mmol) and 0.004 g of catalyst (HMS/Pr-PTSC-Cu) in EtOH were added to a test tube and stirred with a magnetic stirrer at room temperature. Progress of reaction was monitored by TLC. After the completion of reaction the hot EtOH was added and catalyst was separated by filtration. Purification of products was done through recrystallization in EtOH.

### General procedure for the synthesis of 1,4-dihydropyrano[2,3-c]pyrazole by HMS/Pr-PTSC-Cu

0.006 g of HMS/Pr-PTSC-Cu was added to a mixture of aldehyde (1 mmol), hydrazine hydrate (1 mmol), ethyl acetoacetate (1 mmol) and malononitrile (1 mmol) in H_2_O: EtOH (2:1 mL) at room temperature. Completion of the reaction was continuously checked by TLC. After the consumption of the starting material and separation of catalyst, ethyl acetate and H_2_O were added and extracted. The organic layer was dried over Na_2_SO_4._ At the end, the ethyl acetate was evaporated to afford the corresponding product. Finally, purification of products undertake through recrystallization with EtOH.

### Characterization data of selected compounds

#### ^1^H NMR spectra

2–Amino–3–cyano–7,7–dimethyl–4-(3-nitrophenyl)–5–oxo-4H–5,6,7,8-tetrahydro benzopyran (Table 4, entry 4): ^1^H NMR (300 MHz, DMSO-d6): δ = 0.95 (s, 3H), 1.03 (s, 3H), 2.10 (d, J = 15 Hz, 1H), 2.26 (d, J = 15 Hz, 1H), 2.54 (s, 2H), 4.41 (s, 1H), 7.16 (s, 2H), 7.58–7.68 (m, 2H), 7.97 (s, 1H), 8.05–8.08 (m, 1H) ppm.

2–Amino–3–cyano–7,7–dimethyl–4-(2,4-dichlorophenyl)–5–oxo-4H–5,6,7,8-tetrahydro benzopyran (Table 4, entry 8): ^1^H NMR (300 MHz, DMSO-d6): δ = 0.97 (s, 3H), 1.03 (s, 3H), 2.07 (d, J = 15 Hz, 1H), 2.23 (d, J = 15 Hz, 1H), 3.16–3.45 (m, 2H), 4.68 (s, 1H), 7.07 (s, 2H), 7.21 (d, J = 9 Hz, 1H), 7.34 (dd, J = 9 Hz, 1H), 7.50 (d, J = 3 Hz, 1H) ppm.

2–Amino–3–cyano–7,7–dimethyl–4-(3-hydroxyphenyl)–5–oxo-4H–5,6,7,8-tetrahydro benzopyran (Table 8, entry 10): ^1^H NMR (300 MHz, DMSO-d6): δ = 0.96 (s, 3H), 1.03 (s, 3H), 2.09 (d, J = 15 Hz, 1H), 2.24 (d, J = 15 Hz, 1H), 2.42–2.56 (m, 2H), 4.05 (s, 1H), 6.53 (s, 2H), 6.54–6.57 (m, 1H), 6.97–7.07 (m, 3H), 9.31 (s, 1H) ppm.

6-Amino-4-(2,4-dichlorophenyl)-3-methyl-2,4-dihydropyrano[2,3-c]pyrazole-5-carbonitrile (Table 6 entry 2): ^1^H NMR (300 MHz, DMSO-d6): δ = 1.77 (s, 3H), 5.05 (s, 1H), 6.98 (s, 2H), 7.21 (d, J = 9 Hz, 1H), 7.39 (dd, J = 3 Hz, 1H), 7.56 (d, J = 3 Hz, 1H) 12.15 (s, 1H) ppm.

6-Amino-3-methyl-4-(thiophen-2-yl)-2,4-dihydropyrano[2,3-c]pyrazole-5-carbonitrile (Table 6, entry 10): ^1^H NMR (300 MHz, DMSO-d6): δ = 1.91 (s, 3H), 4.98 (s, 1H), 6.91–6.94 (m, 3H), 6.99–7.37 (m, 2H), 12.15 (s, 1H) ppm.

6-Amino-4-(3-hydroxyphenyl)-3-methyl-2,4-dihydropyrano[2,3-c]pyrazole-5-carbonitrile (Table 6, entry 11): ^1^H NMR (300 MHz, DMSO-d6): δ = 1.81 (s, 3H), 4.48 (s, 1H), 6.54–6.63 (m, 3H), 6.83 (s, 2H), 7.09 (t, J = 9 Hz, 1H), 9.29 (s,1H), 12.07 (s, 1H) ppm.

6-Amino-4-(4-bromophenyl)-3-methyl-1,4-dihydropyrano[2,3-c]pyrazole-5-carbonitrile (Table 10, entry 2): ^1^H NMR (300 MHz, DMSO-d6): δ = 1.78 (s, 3H), 4.61 (s, 1H), 6.92 (s, 2H), 7.12 (d, J = 6 Hz, 2H), 7.50 (d, J = 6 Hz, 2H), 12.13 (s, 1H) ppm.

#### FT-IR spectra

2–Amino–3–cyano–7,7–dimethyl–4-(3-nitrophenyl)–5–oxo-4H–5,6,7,8-tetrahydro benzopyran (Table 4, entry 4): IR (KBr, ν) 3433, 3334, 3202, 2958, 2878, 2189,1668, 1529, 1358, 1208, 1091,1033, 819 cm^−1^.

2–Amino–3–cyano–7,7–dimethyl–4-(4-methylphenyl)–5–oxo-4H–5,6,7,8-tetrahydro benzopyran (Table 8, entry 7): IR (KBr, ν) 3449, 3381, 3316, 2961, 2899, 2192, 1655, 1604, 1367, 1249, 1210, 1146, 1030, 765, 559 cm^−1^.

2–Amino–3–cyano–7,7–dimethyl–4-(phenyl)–5–oxo-4H–5,6,7,8-tetrahydro benzopyran (Table 8, entry 8): IR (KBr, ν) 3394, 3324, 3251, 2966, 2881, 2197, 1670, 1603, 1370, 1250, 1212, 1148, 1031, 738, 494 cm^−1^.

4,4'-(1,4-phenylene)bis(2-amino-7,7-dimethyl-5-oxo-5,6,7,8-tetrahydro-4H-chromene-3-carbonitrile) (Table 8, entry 11): IR (KBr, ν) 3639, 3460, 3326, 3191, 2958, 2879, 2193, 1680, 1597, 1467,1365,1252, 1211, 1149, 1031, 824, 564 cm^−1^.

6-Amino-4-(2-hydroxyphenyl)-3-methyl-2,4-dihydropyrano[2,3-c]pyrazole-5-carbonitrile (Table 10, entry 8): IR (KBr, ν) 3613, 3446, 3351, 2187, 1660, 1612, 1531, 1401, 755, 497 cm^−1^.

6-Amino-4-(4-bromophenyl)-3-methyl-1,4-dihydropyrano[2,3-c]pyrazole-5-carbonitrile (Table 10, entry 2): IR (KBr, ν) 3481, 3395, 3182, 2189, 1643, 1600, 1488, 1401, 1046, 798, 537 cm^−1^.

## Conclusions

In conclusion a feasible method was proposed to prepare a highly active and novel mesoporous catalysts. Then, characterization of synthesized catalysts were carried out by several techniques such as FT-IR, TGA, XRD, SEM, TEM, EDS-MAP, adsorption desorption of nitrogen, AAS and ICP. Additionally, we described simple and efficient route for the synthesis of tetrahydrobenzo[b]pyran and 1,4-dihydropyrano[2,3-c]pyrazole derivatives via a one-pot reaction in water/ethanol and ethanol as green solvents, using environmentally benign HMS/Pr-Xa-Ni and HMS/Pr-PTSC-Cu as new mesoporous catalysts. In comparison of these two catalysts, HMS/Pr-PTSC-Cu exhibited higher efficiency in green media under milder reaction conditions at room temperature. In this light, in order to examine the stability of HMS/Pr-PTSC-Cu after recovering, IR and hot filtration test revealed that the structure of catalyst was preserved after recovery. Generally, the advantages of these process including mild reaction conditions, good to high yields, short reaction times, eco-friendly solvent, simple workup, lack of by-products, simple purification of products, economic availability of the materials, environmentally friendly nature and compliance with the green chemistry protocols, simple separation of catalyst, no extraction or separation by column chromatography. Also, easily recoverable of synthesized catalysts and medicinal applications of product are among the other advantages of this method.

## Supplementary Information

Supplementary Information 1.

Supplementary Information 2.

Supplementary Information 3.

Supplementary Information 4.

Supplementary Information 5.

Supplementary Information 6.

Supplementary Information 7.

## Data Availability

All data generated or analysed during this study are included in this published article [and its Supplementary Information files].
