# Supplementary material for: Preparation and catalytic application of two different nanocatalysts based on hexagonal mesoporous silica (HMS) in synthesis of tetrahydrobenzo[b]pyran and 1,4-dihydropyrano[2,3-c]pyrazole derivatives
Source: Sci Rep. 2022 Dec 21;12:22108. doi: 10.1038/s41598-022-26605-0 (PMC9772405; doi:10.1038/s41598-022-26605-0)
Supplement: Supplementary file 1 — Supplementary Information 1. [file 41598_2022_26605_MOESM1_ESM.zip › BET-HMS-Pr-PTSC-Cu (Raw data1)/BET-Abdolahi2/A-HMS.pdf]

Sample: A-HMSAL_1
Operator: dianat
File: C:\2020\DATA\A-HMS.SMP

Started:	2020/08/21 10:26:34?.?	Analysis Adsorptive:	N2
Completed:	2020/08/22 5:24:58?.?	Analysis Bath Temp.:	77.225 K
Report Time:	2020/08/22 11:42:49?.?	Thermal Correction:	No
Sample Mass:	0.0970 g	Warm Free Space:	26.0524 cm³ Measured
Cold Free Space:	79.4615 cm³	Equilibration Interval:	10 s
Low Pressure Dose:	None	Automatic Degas:	Yes

Summary Report

Surface Area

Single point surface area at p/p° = 0.200635326: 305 .4456 m²/g

BET Surface Area: 325.1223 m²/g

Langmuir Surface Area: 456.0524 m²/g

t-Plot Micropore Area: 30.1942 m²/g

t-Plot External Surface Area: 294.9281 m²/g

BJH Adsorption cumulative surface area of pores between 17.000 Å and 3000.000 Å diameter: 203.178 m²/g

BJH Desorption cumulative surface area of pores between 17.000 Å and 3000.000 Å diameter: 221.4694 m²/g

Pore Volume

Single point adsorption total pore volume of pores less than 737.922 Å diameter at p/p° = 0.973054930: 0.320296 cm³/g

t-Plot micropore volume: 0.005981 cm³/g

BJH Adsorption cumulative volume of pores between 17.000 Å and 3000.000 Å diameter: 0.356726 cm³/g

BJH Desorption cumulative volume of pores between 17.000 Å and 3000.000 Å diameter: 0.359611 cm³/g

Pore Size

Adsorption average pore width (4V/A by BET): 39.4062 Å

BJH Adsorption average pore diameter (4V/A): 70.229 Å

BJH Desorption average pore diameter (4V/A): 64.950 Å

Sample: A-HMSAL_1
Operator: dianat
File: C:\2020\DATA\A-HMS.SMP

Started: 2020/08/21 10:26:34?.?
Completed: 2020/08/22 5:24:58?.?
Report Time: 2020/08/22 11:42:49?.?
Sample Mass: 0.0970 g
Cold Free Space: 79.4615 cm³
Low Pressure Dose: None

Analysis Adsorptive: N2
Analysis Bath Temp.: 77.225 K
Thermal Correction: No
Warm Free Space: 26.0524 cm³ Measured
Equilibration Interval: 10 s
Automatic Degas: Yes

Isotherm Tabular Report

Relative Pressure (p/p°)	Absolute Pressure (kPa)	Quantity Adsorbed (cm³/g STP)	Elapsed Time (h:min)	Saturation Pressure (kPa)
			01:27	99.8905469
0.010408456	1.0394142	44.3155	02:05	
0.031062196	3.1016494	55.6019	02:18	
0.061299431	6.1205618	64.6620	02:26	
0.077856366	7.7734338	68.5548	02:31	
0.099771649	9.9610850	73.0240	02:37	
0.119838502	11.9641820	76.6394	02:41	
0.139922251	13.9687438	79.8933	02:46	
0.160116333	15.9842900	82.8124	02:50	
0.180335078	18.0023119	85.4425	02:53	
0.200635326	20.0282322	87.7769	02:57	
0.249752816	24.9306012	92.1435	03:01	
0.302561342	30.2013360	95.7716	03:04	
0.353182303	35.2534810	98.7561	03:07	
0.399904305	39.9162294	101.3195	03:10	
0.449938812	44.9093990	104.0153	03:13	
0.499992987	49.9043100	106.8500	03:16	
0.551070105	55.0006898	110.0833	03:20	
0.601026185	59.9853274	114.2585	03:23	
0.650932399	64.9638050	120.1998	03:28	
			03:30	99.7996771
0.701401494	69.9989715	128.0127	03:34	
0.750076069	74.8557347	136.9487	03:39	
0.800586250	79.8953768	147.9189	03:45	
0.821473942	81.9791007	152.9367	03:49	
0.851319274	84.9565014	160.2818	03:54	
0.875748451	87.3933377	166.2928	03:59	
0.900283448	89.8408909	172.6436	04:03	
0.924943774	92.3006827	180.1134	04:08	
0.949826740	94.7826325	190.4299	04:13	
0.973054930	97.0989301	207.0700	04:20	
0.981703208	97.9605131	218.4204	04:26	
0.989721540	98.7587387	236.3164	04:34	
0.994570029	99.2408769	256.6008	04:41	
0.983691111	98.1537020	242.0838	04:48	
0.974288680	97.2138865	225.6055	04:55	
0.951251097	94.9131637	201.9702	05:04	
0.929620359	92.7535752	190.3727	05:10	
0.905752149	90.3710232	181.9276	05:15	
0.878175886	87.6185634	174.6897	05:20	
0.851578650	84.9640529	169.2998	05:24	
0.826778730	82.4889141	164.9817	05:28	
0.801322341	79.9483265	161.0807	05:32	
			05:34	99.7700164
0.751284689	74.9556858	154.3368	05:38	
0.700716310	69.9104778	147.1876	05:43	
0.650162531	64.8667264	139.2771	05:48	
0.599046877	59.7669167	131.6720	05:53	
0.549390637	54.8127128	125.4154	05:57	
0.499624330	49.8475276	120.5541	06:01	
0.455919604	45.4871063	109.9483	06:10	

Sample: A-HMSAL_1
Operator: dianat
File: C:\2020\DATA\A-HMS.SMP

Started: 2020/08/21 10:26:34?.?
Completed: 2020/08/22 5:24:58?.?
Report Time: 2020/08/22 11:42:49?.?
Sample Mass: 0.0970 g
Cold Free Space: 79.4615 cm³
Low Pressure Dose: None

Analysis Adsorptive: N2
Analysis Bath Temp.: 77.225 K
Thermal Correction: No
Warm Free Space: 26.0524 cm³ Measured
Equilibration Interval: 10 s
Automatic Degas: Yes

Isotherm Tabular Report

Relative Pressure (p/p°)	Absolute Pressure (kPa)	Quantity Adsorbed (cm^3/g STP)	Elapsed Time (h:min)	Saturation Pressure (kPa)
0.395488406	39.4578848	104.0810	06:14	
0.333294490	33.2527967	100.5887	06:19	
0.282897858	28.2247240	97.4986	06:23	
0.250048121	24.9473051	95.1725	06:26	
0.200340558	19.9879808	90.8041	06:31	
0.145110289	14.4776559	83.9215	06:36	

micromeritics®

Full Report Set

Sample: A-HMSAL_1
Operator: dianat
File: C:\2020\DATA\A-HMS.SMP

Started:	2020/08/21 10:26:34?.?	Analysis Adsorptive:	N2
Completed:	2020/08/22 5:24:58?.?	Analysis Bath Temp.:	77.225 K
Report Time:	2020/08/22 11:42:49?.?	Thermal Correction:	No
Sample Mass:	0.0970 g	Warm Free Space:	26.0524 cm³ Measured
Cold Free Space:	79.4615 cm³	Equilibration Interval:	10 s
Low Pressure Dose:	None	Automatic Degas:	Yes

Isotherm Linear Plot

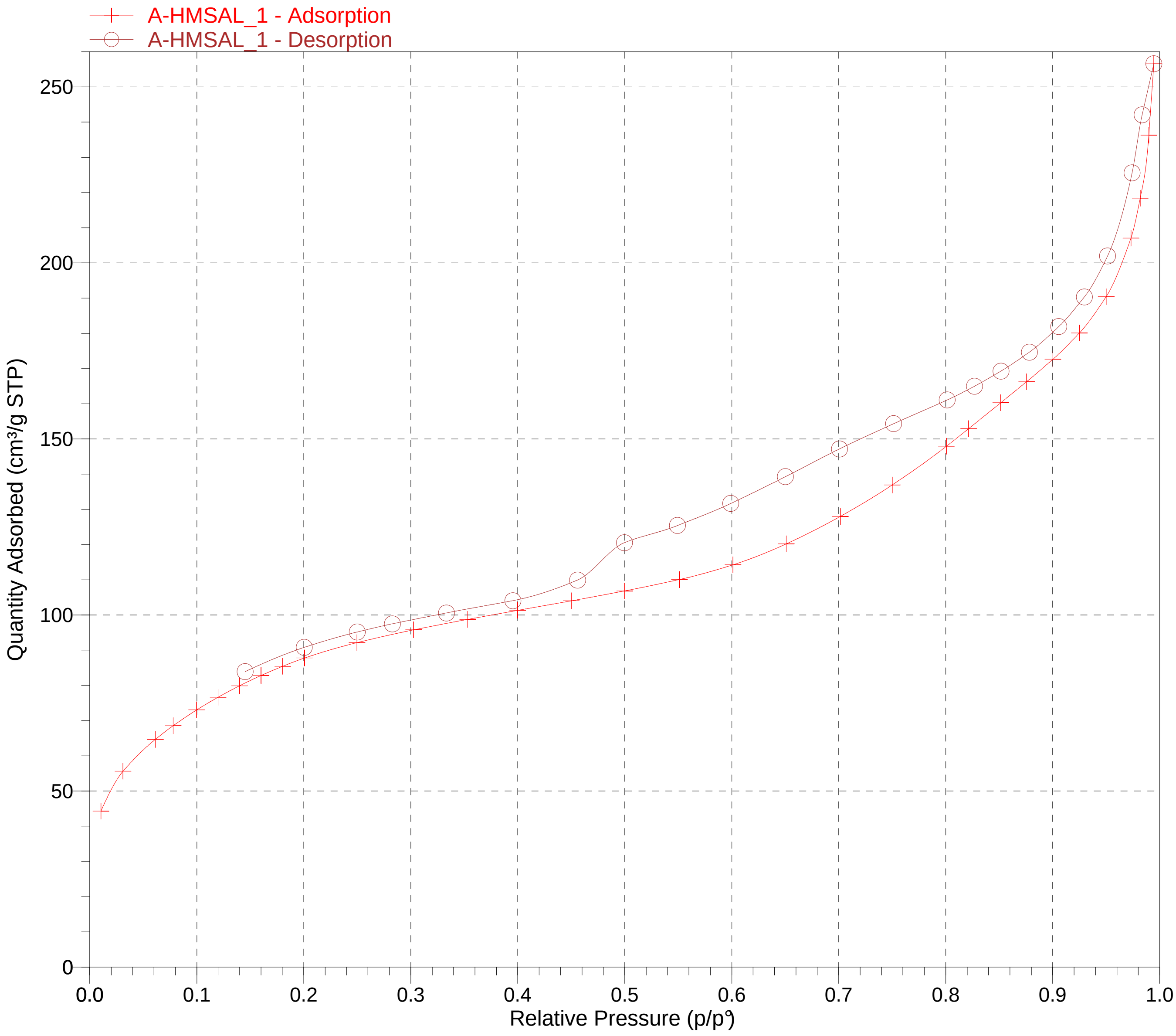

Sample: A-HMSAL_1
Operator: dianat
File: C:\2020\DATA\A-HMS.SMP

Started:	2020/08/21 10:26:34?.?	Analysis Adsorptive:	N2
Completed:	2020/08/22 5:24:58?.?	Analysis Bath Temp.:	77.225 K
Report Time:	2020/08/22 11:42:49?.?	Thermal Correction:	No
Sample Mass:	0.0970 g	Warm Free Space:	26.0524 cm³ Measured
Cold Free Space:	79.4615 cm³	Equilibration Interval:	10 s
Low Pressure Dose:	None	Automatic Degas:	Yes

Isotherm Log Plot

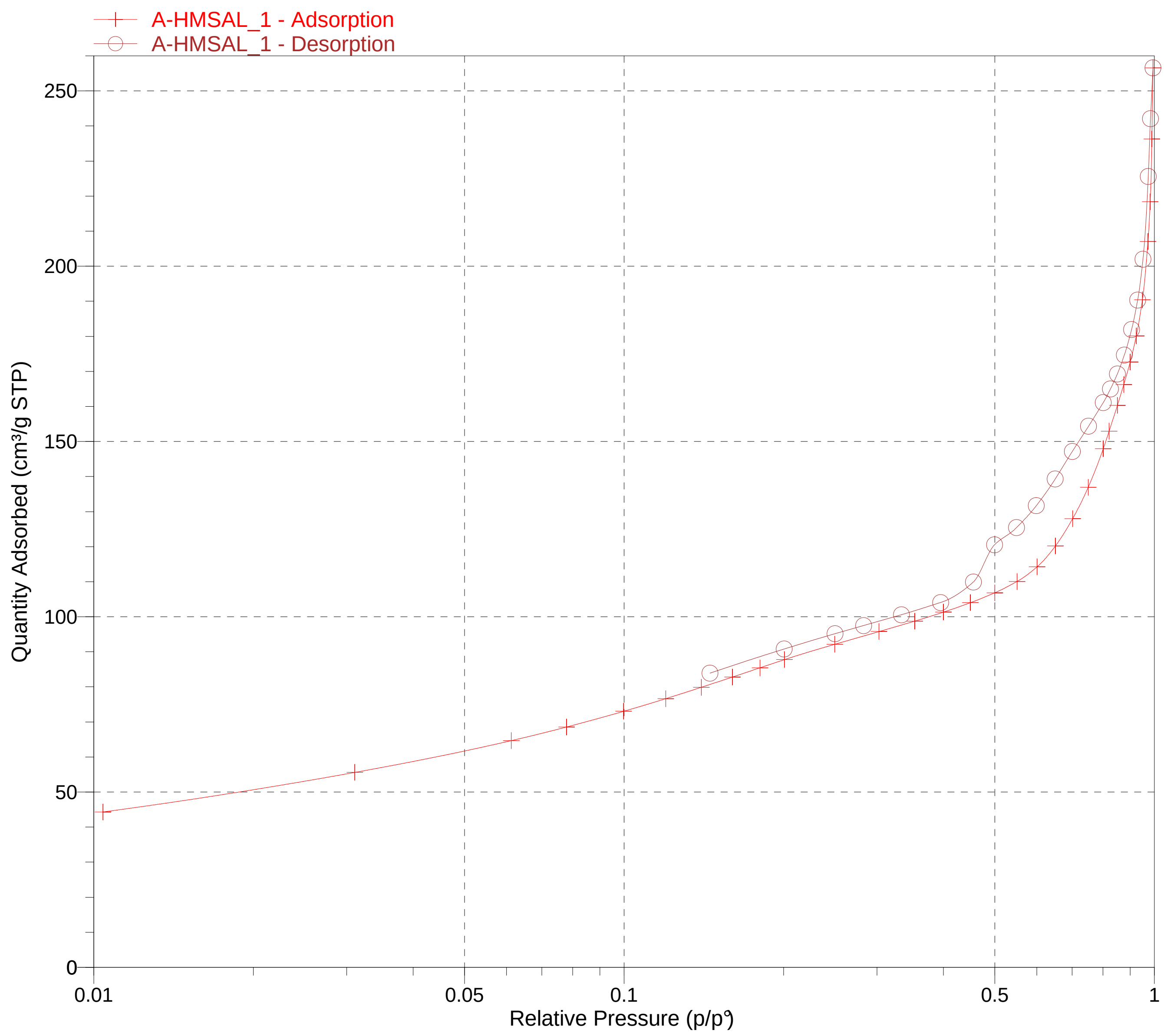

Sample: A-HMSAL_1
Operator: dianat
File: C:\2020\DATA\A-HMS.SMP

Started: 2020/08/21 10:26:34?.?
Completed: 2020/08/22 5:24:58?.?
Report Time: 2020/08/22 11:42:49?.?
Sample Mass: 0.0970 g
Cold Free Space: 79.4615 cm^3
Low Pressure Dose: None

Analysis Adsorptive: N_2
Analysis Bath Temp.: 77.225 K
Thermal Correction: No
Warm Free Space: 26.0524 cm^3 Measured
Equilibration Interval: 10 s
Automatic Degas: Yes

BET Surface Area Report

BET Surface Area: 325.1223 ± 1.5472 m^2/g
Slope: 0.013190 ± 0.000063 g/cm^3 STP
Y-Intercept: 0.000199 ± 0.000009 g/cm^3 STP
C: 67.212973
Qm: 74.6858 cm^3/g STP
Correlation Coefficient: 0.9999313
Molecular Cross-Sectional Area: 0.1620 nm^2

Relative Pressure (p/p°)	Quantity Adsorbed (cm^3/g STP)	1/[Q(p°/p - 1)]
0.061299431	64.6620	0.001010
0.077856366	68.5548	0.001232
0.099771649	73.0240	0.001518
0.119838502	76.6394	0.001777
0.139922251	79.8933	0.002036
0.160116333	82.8124	0.002302
0.180335078	85.4425	0.002575
0.200635326	87.7769	0.002859

Sample: A-HMSAL_1
Operator: dianat
File: C:\2020\DATA\A-HMS.SMP

Started: 2020/08/21 10:26:34?.?
Completed: 2020/08/22 5:24:58?.?
Report Time: 2020/08/22 11:42:49?.?
Sample Mass: 0.0970 g
Cold Free Space: 79.4615 cm³
Low Pressure Dose: None

Analysis Adsorptive: N2
Analysis Bath Temp.: 77.225 K
Thermal Correction: No
Warm Free Space: 26.0524 cm³ Measured
Equilibration Interval: 10 s
Automatic Degas: Yes

BET Surface Area Plot

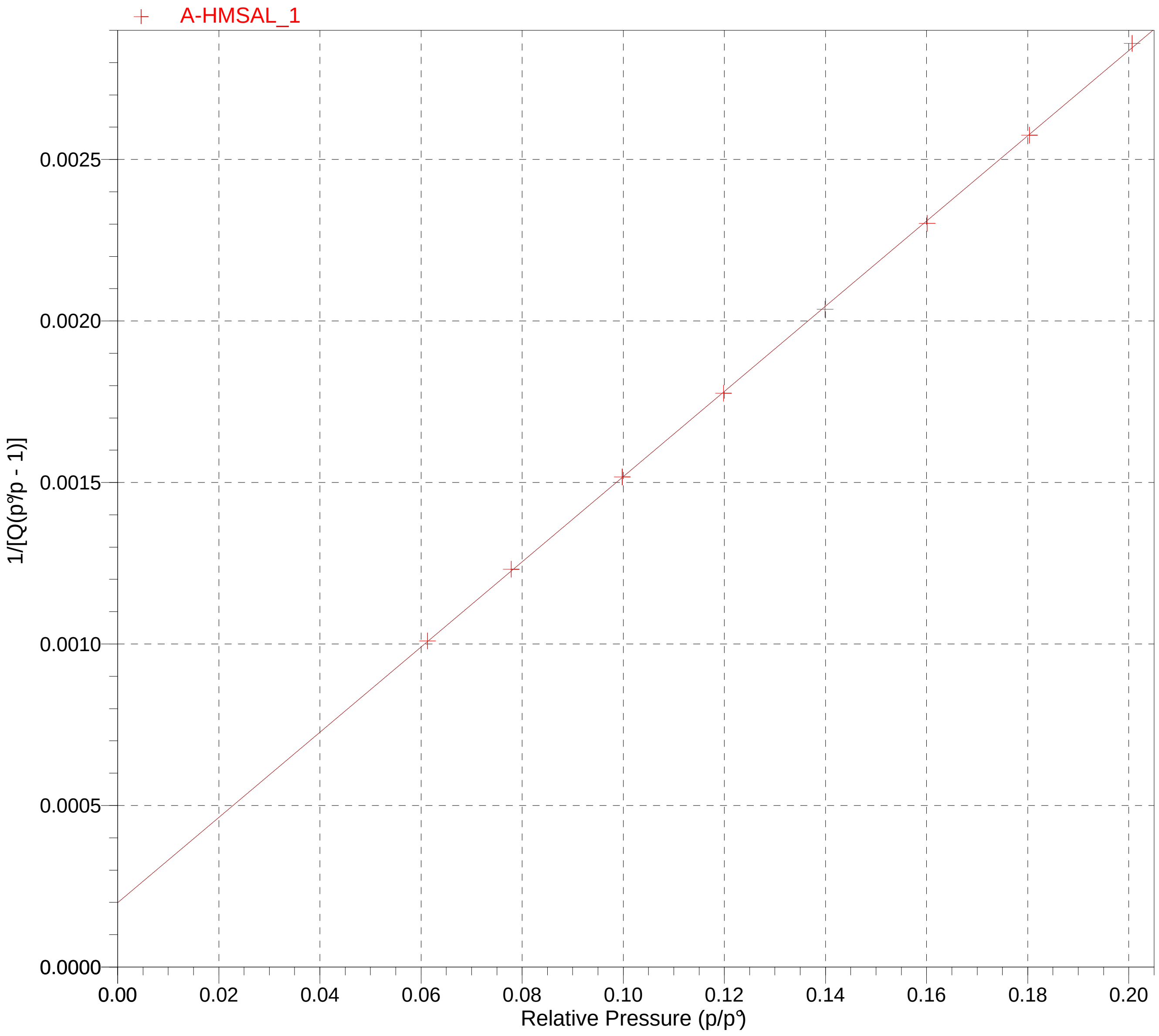

Sample: A-HMSAL_1
Operator: dianat
File: C:\2020\DATA\A-HMS.SMP

Started: 2020/08/21 10:26:34?.?
Completed: 2020/08/22 5:24:58?.?
Report Time: 2020/08/22 11:42:49?.?
Sample Mass: 0.0970 g
Cold Free Space: 79.4615 cm³
Low Pressure Dose: None

Analysis Adsorptive: N2
Analysis Bath Temp.: 77.225 K
Thermal Correction: No
Warm Free Space: 26.0524 cm³ Measured
Equilibration Interval: 10 s
Automatic Degas: Yes

Langmuir Surface Area Report

Langmuir Surface Area: 456.0524 ± 8.4133 m²/g
Slope: 0.009545 ± 0.000176 g/cm³ STP
Y-Intercept: 0.039556 ± 0.002424 kPa·g/cm³ STP
b: 0.241310 1/kPa
Qm: 104.7626 cm³/g STP
Correlation Coefficient: 0.998981
Molecular Cross-Sectional Area: 0.1620 nm²

Pressure (kPa)	Quantity Adsorbed (cm³/g STP)	p/Q (kPa·g/cm³ STP)
6.1205618	64.6620	0.0947
7.7734338	68.5548	0.1134
9.9610850	73.0240	0.1364
11.9641820	76.6394	0.1561
13.9687438	79.8933	0.1748
15.9842900	82.8124	0.1930
18.0023119	85.4425	0.2107
20.0282322	87.7769	0.2282

Sample: A-HMSAL_1
Operator: dianat
File: C:\2020\DATA\A-HMS.SMP

Started:	2020/08/21 10:26:34?.?	Analysis Adsorptive:	N2
Completed:	2020/08/22 5:24:58?.?	Analysis Bath Temp.:	77.225 K
Report Time:	2020/08/22 11:42:49?.?	Thermal Correction:	No
Sample Mass:	0.0970 g	Warm Free Space:	26.0524 cm³ Measured
Cold Free Space:	79.4615 cm³	Equilibration Interval:	10 s
Low Pressure Dose:	None	Automatic Degas:	Yes

Langmuir Surface Area Plot

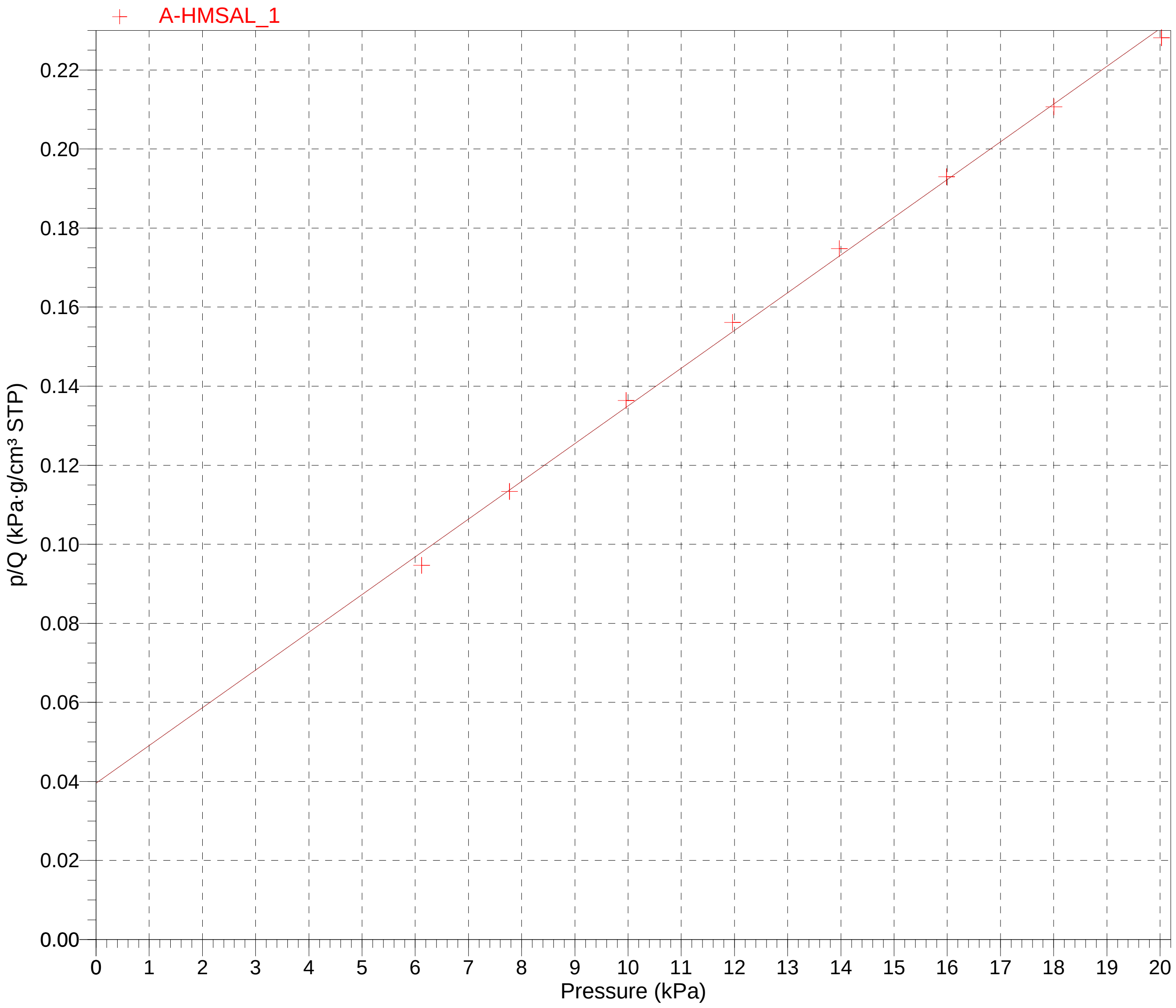

Sample: A-HMSAL_1
Operator: dianat
File: C:\2020\DATA\A-HMS.SMP

Started: 2020/08/21 10:26:34?.?
Completed: 2020/08/22 5:24:58?.?
Report Time: 2020/08/22 11:42:49?.?
Sample Mass: 0.0970 g
Cold Free Space: 79.4615 cm³
Low Pressure Dose: None

Analysis Adsorptive: N2
Analysis Bath Temp.: 77.225 K
Thermal Correction: No
Warm Free Space: 26.0524 cm³ Measured
Equilibration Interval: 10 s
Automatic Degas: Yes

t-Plot Report

Micropore Volume: 0.005981 cm³/g
Micropore Area: 30.1942 m²/g
External Surface Area: 294.9281 m²/g
Slope: 19.066983 ± 0.995664 cm³/g·Å STP
Y-Intercept: 3.866372 ± 4.120090 cm³/g STP
Correlation Coefficient: 0.993252
Surface Area Correction Factor: 1.000
Density Conversion Factor: 0.0015468
Total Surface Area (BET): 325.1223 m²/g
Thickness Range: 3.5000 Å to 5.0000 Å
Thickness Equation: Harkins and Jura

$t = [\ 13.99 / (\ 0.034 - \log(p/p^{\circ})\ )\ ]\ ^\wedge\ 0.5$

Relative Pressure (p/p°)	Statistical Thickness (Å)	Quantity Adsorbed (cm³/g STP)
0.010408456	2.6339	44.3155
0.031062196	3.0123	55.6019
0.061299431	3.3501	64.6620
0.077856366	3.4990	68.5548
0.099771649	3.6765	73.0240
0.119838502	3.8266	76.6394
0.139922251	3.9689	79.8933
0.160116333	4.1066	82.8124
0.180335078	4.2407	85.4425
0.200635326	4.3729	87.7769
0.249752816	4.6883	92.1435
0.302561342	5.0289	95.7716
0.353182303	5.3653	98.7561
0.399904305	5.6904	101.3195
0.449938812	6.0609	104.0153
0.499992987	6.4619	106.8500
0.551070105	6.9124	110.0833
0.601026185	7.4054	114.2585
0.650932399	7.9660	120.1998

Sample: A-HMSAL_1
Operator: dianat
File: C:\2020\DATA\A-HMS.SMP

Started:	2020/08/21 10:26:34?.?	Analysis Adsorptive:	N2
Completed:	2020/08/22 5:24:58?.?	Analysis Bath Temp.:	77.225 K
Report Time:	2020/08/22 11:42:49?.?	Thermal Correction:	No
Sample Mass:	0.0970 g	Warm Free Space:	26.0524 cm³ Measured
Cold Free Space:	79.4615 cm³	Equilibration Interval:	10 s
Low Pressure Dose:	None	Automatic Degas:	Yes

t-Plot

Harkins and Jura

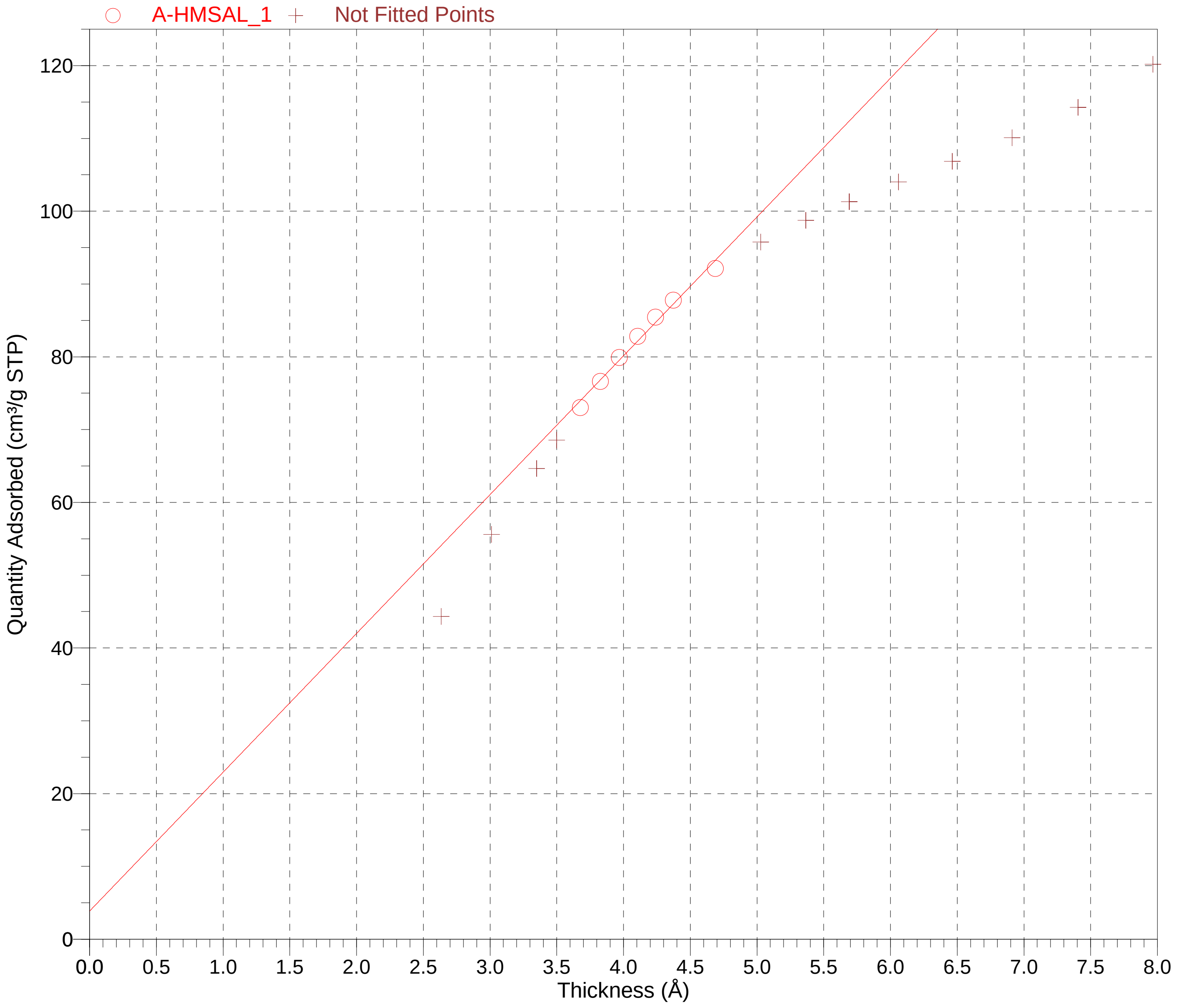

Sample: A-HMSAL_1
Operator: dianat
File: C:\2020\DATA\A-HMS.SMP

Started: 2020/08/21 10:26:34?.?
Completed: 2020/08/22 5:24:58?.?
Report Time: 2020/08/22 11:42:49?.?
Sample Mass: 0.0970 g
Cold Free Space: 79.4615 cm³
Low Pressure Dose: None

Analysis Adsorptive: N2
Analysis Bath Temp.: 77.225 K
Thermal Correction: No
Warm Free Space: 26.0524 cm³ Measured
Equilibration Interval: 10 s
Automatic Degas: Yes

BJH Adsorption Pore Distribution Report

Faas Correction
Halsey
t = 3.54 [-5 / ln(p/p°)] ^ 0.333

Diameter Range: 17.000 Å to 3000.000 Å
Adsorbate Property Factor: 9.53000 Å
Density Conversion Factor: 0.0015468
Fraction of Pores Open at Both Ends: 0.00

Pore Diameter Range (Å)	Average Diameter (Å)	Incremental Pore Volume (cm³/g)	Cumulative Pore Volume (cm³/g)	Incremental Pore Area (m²/g)	Cumulative Pore Area (m²/g)
3569.3 - 1900.3	2263.6	0.032972	0.032972	0.583	0.583
1900.3 - 1077.9	1274.3	0.029481	0.062453	0.925	1.508
1077.9 - 737.9	844.7	0.018900	0.081354	0.895	2.403
737.9 - 402.8	476.7	0.028619	0.109972	2.401	4.804
402.8 - 272.6	312.2	0.018130	0.128103	2.323	7.127
272.6 - 207.1	230.4	0.013489	0.141592	2.342	9.469
207.1 - 167.4	182.7	0.011850	0.153442	2.594	12.063
167.4 - 140.6	151.5	0.011638	0.165080	3.073	15.137
140.6 - 117.7	126.9	0.014828	0.179908	4.673	19.809
117.7 - 105.7	111.0	0.010432	0.190340	3.761	23.570
105.7 - 84.6	92.5	0.023702	0.214042	10.248	33.818
84.6 - 70.8	76.3	0.019917	0.233959	10.440	44.257
70.8 - 60.4	64.7	0.017829	0.251788	11.029	55.287
60.4 - 52.6	55.9	0.013411	0.265199	9.604	64.890
52.6 - 46.4	49.0	0.008732	0.273931	7.128	72.018
46.4 - 41.2	43.4	0.006156	0.280087	5.676	77.694
41.2 - 36.9	38.7	0.005355	0.285442	5.528	83.222
36.9 - 33.3	34.8	0.005240	0.290681	6.017	89.239
33.3 - 30.3	31.6	0.005366	0.296047	6.799	96.038
30.3 - 27.3	28.6	0.006941	0.302988	9.706	105.744
27.3 - 24.6	25.8	0.009755	0.312743	15.141	120.885
24.6 - 22.2	23.2	0.013961	0.326704	24.043	144.928
22.2 - 21.2	21.7	0.008479	0.335183	15.636	160.564
21.2 - 20.3	20.7	0.010030	0.345213	19.346	179.910
20.3 - 19.4	19.8	0.011513	0.356726	23.268	203.178

Sample: A-HMSAL_1
Operator: dianat
File: C:\2020\DATA\A-HMS.SMP

Started:	2020/08/21 10:26:34?.?	Analysis Adsorptive:	N2
Completed:	2020/08/22 5:24:58?.?	Analysis Bath Temp.:	77.225 K
Report Time:	2020/08/22 11:42:49?.?	Thermal Correction:	No
Sample Mass:	0.0970 g	Warm Free Space:	26.0524 cm³ Measured
Cold Free Space:	79.4615 cm³	Equilibration Interval:	10 s
Low Pressure Dose:	None	Automatic Degas:	Yes

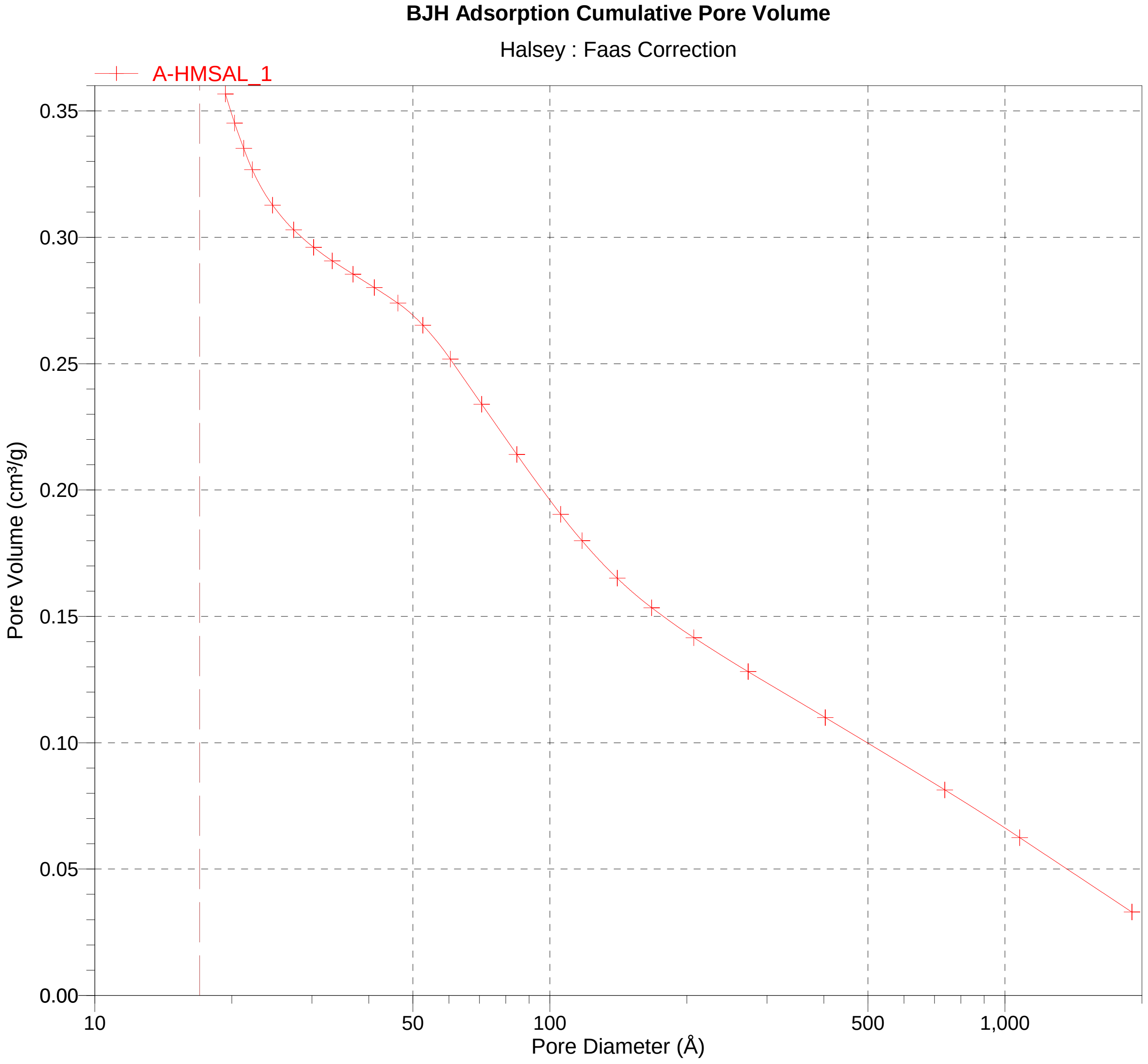

Sample: A-HMSAL_1
Operator: dianat
File: C:\2020\DATA\A-HMS.SMP

Started:	2020/08/21 10:26:34?.?	Analysis Adsorptive:	N2
Completed:	2020/08/22 5:24:58?.?	Analysis Bath Temp.:	77.225 K
Report Time:	2020/08/22 11:42:49?.?	Thermal Correction:	No
Sample Mass:	0.0970 g	Warm Free Space:	26.0524 cm³ Measured
Cold Free Space:	79.4615 cm³	Equilibration Interval:	10 s
Low Pressure Dose:	None	Automatic Degas:	Yes

BJH Adsorption dV/dD Pore Volume

Halsey : Faas Correction

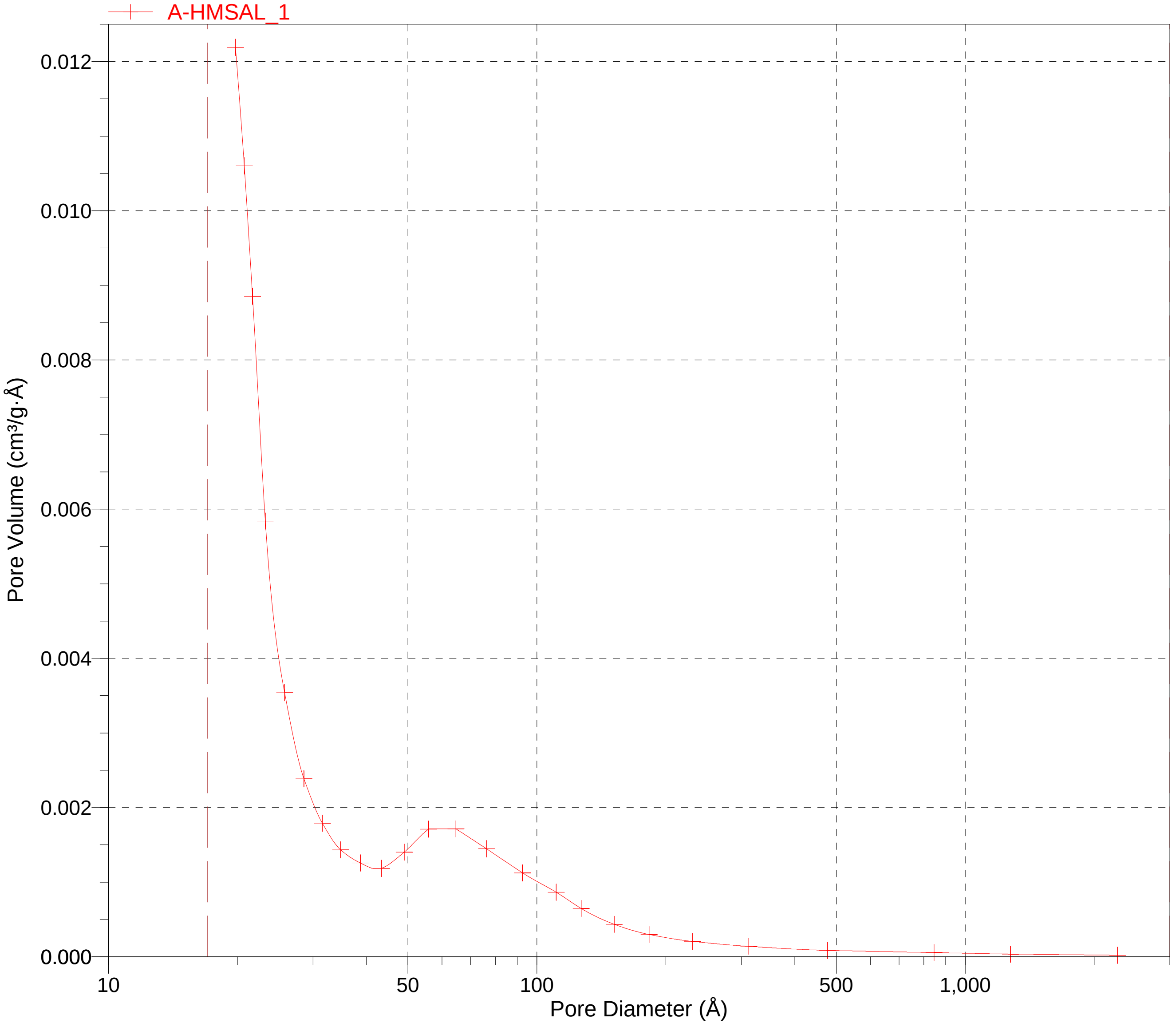

Sample: A-HMSAL_1
Operator: dianat
File: C:\2020\DATA\A-HMS.SMP

Started:	2020/08/21 10:26:34?.?	Analysis Adsorptive:	N2
Completed:	2020/08/22 5:24:58?.?	Analysis Bath Temp.:	77.225 K
Report Time:	2020/08/22 11:42:49?.?	Thermal Correction:	No
Sample Mass:	0.0970 g	Warm Free Space:	26.0524 cm³ Measured
Cold Free Space:	79.4615 cm³	Equilibration Interval:	10 s
Low Pressure Dose:	None	Automatic Degas:	Yes

BJH Adsorption dV/dlog(D) Pore Volume

Halsey : Faas Correction

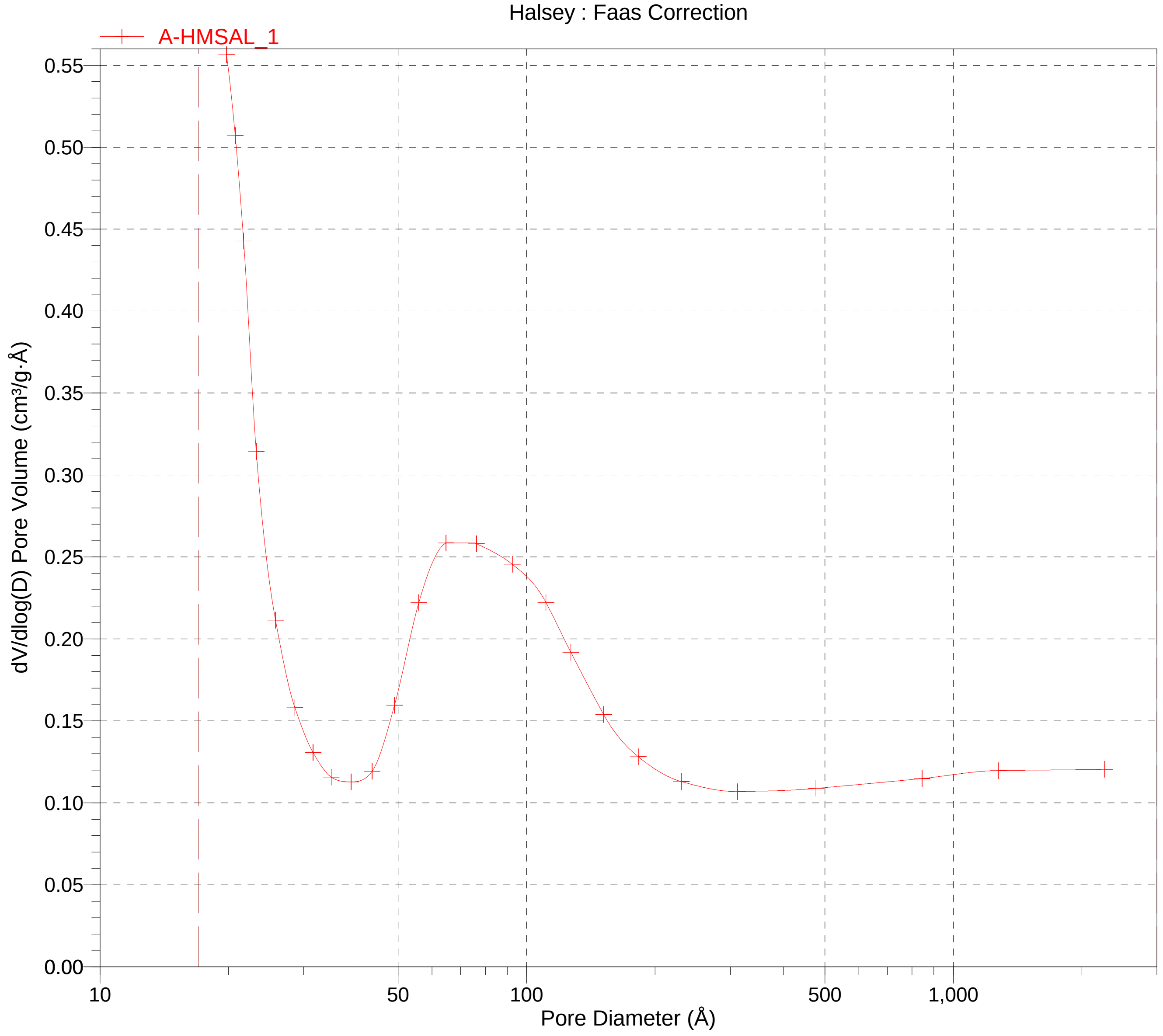

Sample: A-HMSAL_1
Operator: dianat
File: C:\2020\DATA\A-HMS.SMP

Started:	2020/08/21 10:26:34?.?	Analysis Adsorptive:	N2
Completed:	2020/08/22 5:24:58?.?	Analysis Bath Temp.:	77.225 K
Report Time:	2020/08/22 11:42:49?.?	Thermal Correction:	No
Sample Mass:	0.0970 g	Warm Free Space:	26.0524 cm³ Measured
Cold Free Space:	79.4615 cm³	Equilibration Interval:	10 s
Low Pressure Dose:	None	Automatic Degas:	Yes

BJH Adsorption Cumulative Pore Area

Halsey : Faas Correction

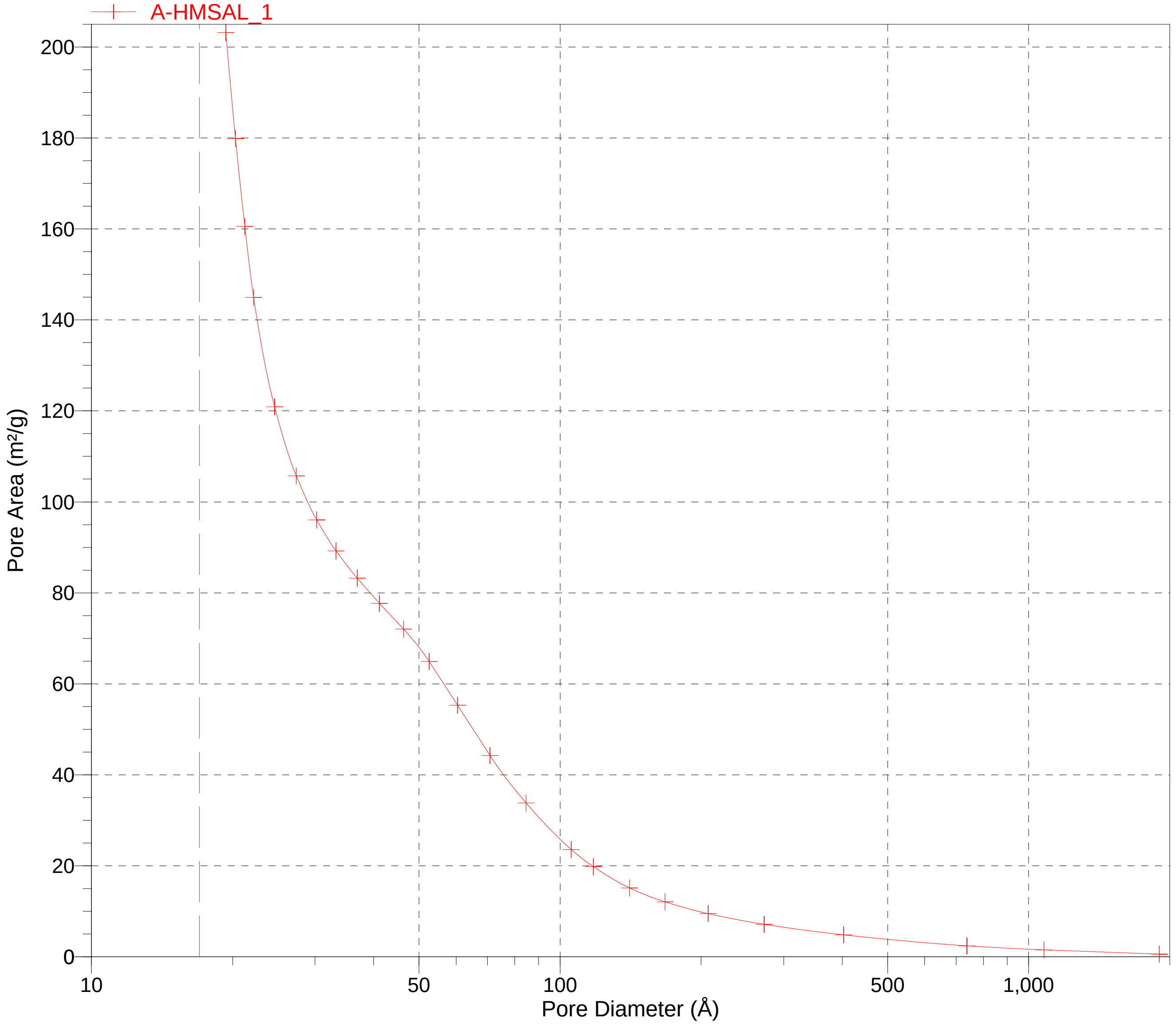

Full Report Set

Sample: A-HMSAL_1
Operator: dianat
File: C:\2020\DATA\A-HMS.SMP

Started:	2020/08/21 10:26:34?.?	Analysis Adsorptive:	N2
Completed:	2020/08/22 5:24:58?.?	Analysis Bath Temp.:	77.225 K
Report Time:	2020/08/22 11:42:49?.?	Thermal Correction:	No
Sample Mass:	0.0970 g	Warm Free Space:	26.0524 cm³ Measured
Cold Free Space:	79.4615 cm³	Equilibration Interval:	10 s
Low Pressure Dose:	None	Automatic Degas:	Yes

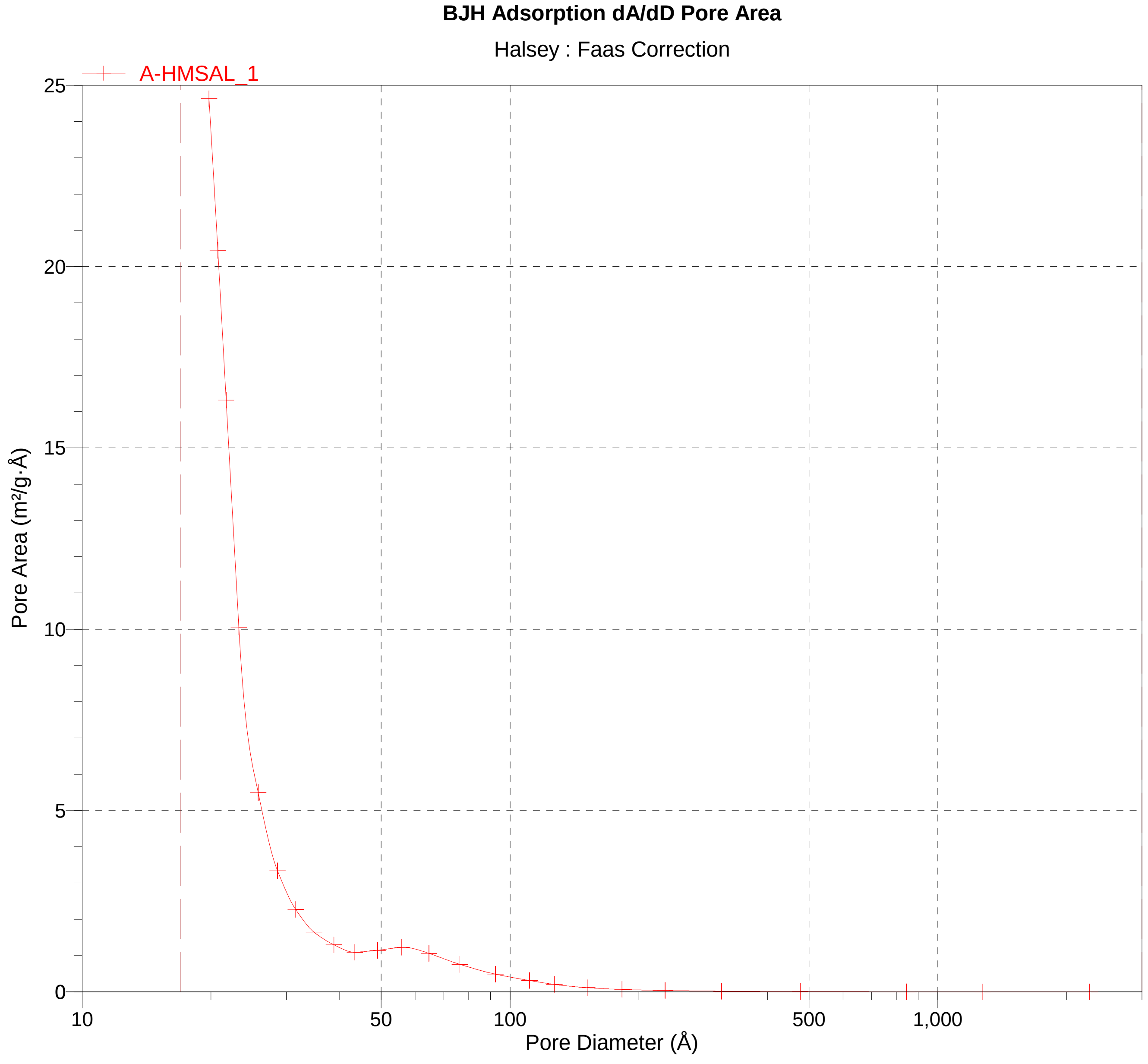

Sample: A-HMSAL_1
Operator: dianat
File: C:\2020\DATA\A-HMS.SMP

Started:	2020/08/21 10:26:34?.?	Analysis Adsorptive:	N2
Completed:	2020/08/22 5:24:58?.?	Analysis Bath Temp.:	77.225 K
Report Time:	2020/08/22 11:42:49?.?	Thermal Correction:	No
Sample Mass:	0.0970 g	Warm Free Space:	26.0524 cm³ Measured
Cold Free Space:	79.4615 cm³	Equilibration Interval:	10 s
Low Pressure Dose:	None	Automatic Degas:	Yes

BJH Adsorption dA/dlog(D) Pore Area

Halsey : Faas Correction

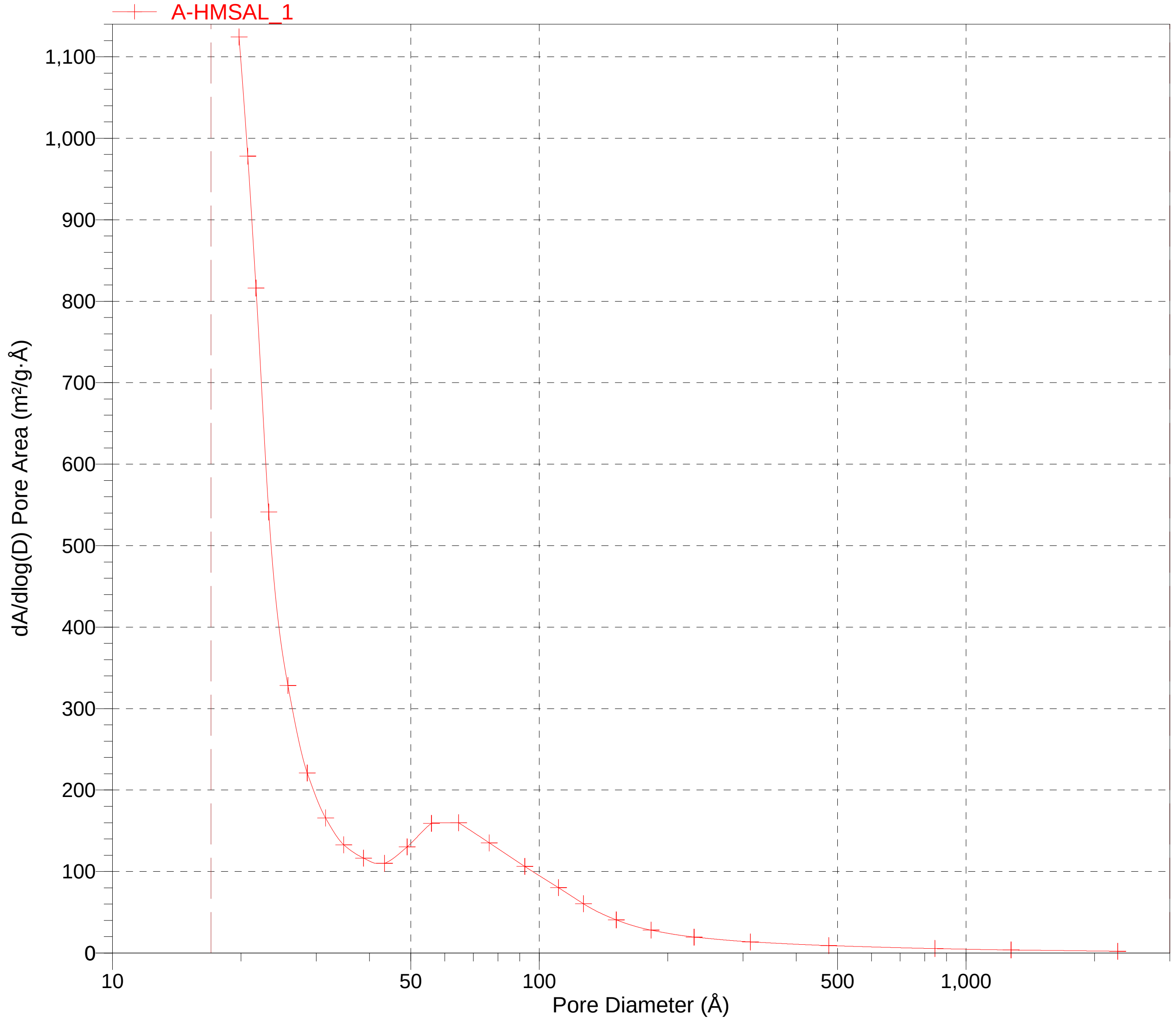

Sample: A-HMSAL_1
Operator: dianat
File: C:\2020\DATA\A-HMS.SMP

Started: 2020/08/21 10:26:34?.?
Completed: 2020/08/22 5:24:58?.?
Report Time: 2020/08/22 11:42:49?.?
Sample Mass: 0.0970 g
Cold Free Space: 79.4615 cm³
Low Pressure Dose: None

Analysis Adsorptive: N2
Analysis Bath Temp.: 77.225 K
Thermal Correction: No
Warm Free Space: 26.0524 cm³ Measured
Equilibration Interval: 10 s
Automatic Degas: Yes

BJH Desorption Pore Distribution Report

Faas Correction
Halsey
$t = 3.54 [-5 / \ln(p/p^0)] ^ 0.333$

Diameter Range: 17.000 Å to 3000.000 Å
Adsorbate Property Factor: 9.53000 Å
Density Conversion Factor: 0.0015468
Fraction of Pores Open at Both Ends: 0.00

Pore Diameter Range (Å)	Average Diameter (Å)	Incremental Pore Volume (cm³/g)	Cumulative Pore Volume (cm³/g)	Incremental Pore Area (m²/g)	Cumulative Pore Area (m²/g)
3569.3 - 1206.7	1441.0	0.024013	0.024013	0.667	0.667
1206.7 - 772.5	896.4	0.027736	0.051749	1.238	1.904
772.5 - 414.2	491.1	0.041145	0.092893	3.352	5.256
414.2 - 290.1	329.8	0.020389	0.113283	2.473	7.728
290.1 - 218.7	243.9	0.015111	0.128394	2.478	10.207
218.7 - 170.6	188.3	0.013267	0.141660	2.818	13.025
170.6 - 140.9	152.7	0.010075	0.151735	2.640	15.665
140.9 - 121.2	129.4	0.008274	0.160009	2.557	18.222
121.2 - 106.0	112.5	0.007647	0.167655	2.719	20.941
106.0 - 85.0	92.9	0.013798	0.181453	5.939	26.880
85.0 - 70.7	76.3	0.015797	0.197250	8.279	35.159
70.7 - 60.3	64.5	0.018805	0.216055	11.659	46.818
60.3 - 52.3	55.6	0.018802	0.234857	13.520	60.338
52.3 - 46.2	48.8	0.015584	0.250440	12.779	73.117
46.2 - 41.1	43.3	0.011684	0.262124	10.795	83.912
41.1 - 37.4	39.0	0.032964	0.295088	33.782	117.694
37.4 - 33.0	34.8	0.014127	0.309216	16.233	133.927
33.0 - 29.1	30.7	0.004952	0.314167	6.452	140.379
29.1 - 26.3	27.5	0.005383	0.319551	7.831	148.210
26.3 - 24.6	25.4	0.004990	0.324541	7.867	156.078
24.6 - 22.2	23.2	0.011687	0.336228	20.130	176.207
22.2 - 19.6	20.7	0.023383	0.359611	45.262	221.469

Sample: A-HMSAL_1
Operator: dianat
File: C:\2020\DATA\A-HMS.SMP

Started:	2020/08/21 10:26:34?.?	Analysis Adsorptive:	N2
Completed:	2020/08/22 5:24:58?.?	Analysis Bath Temp.:	77.225 K
Report Time:	2020/08/22 11:42:49?.?	Thermal Correction:	No
Sample Mass:	0.0970 g	Warm Free Space:	26.0524 cm³ Measured
Cold Free Space:	79.4615 cm³	Equilibration Interval:	10 s
Low Pressure Dose:	None	Automatic Degas:	Yes

BJH Desorption Cumulative Pore Volume

Halsey : Faas Correction

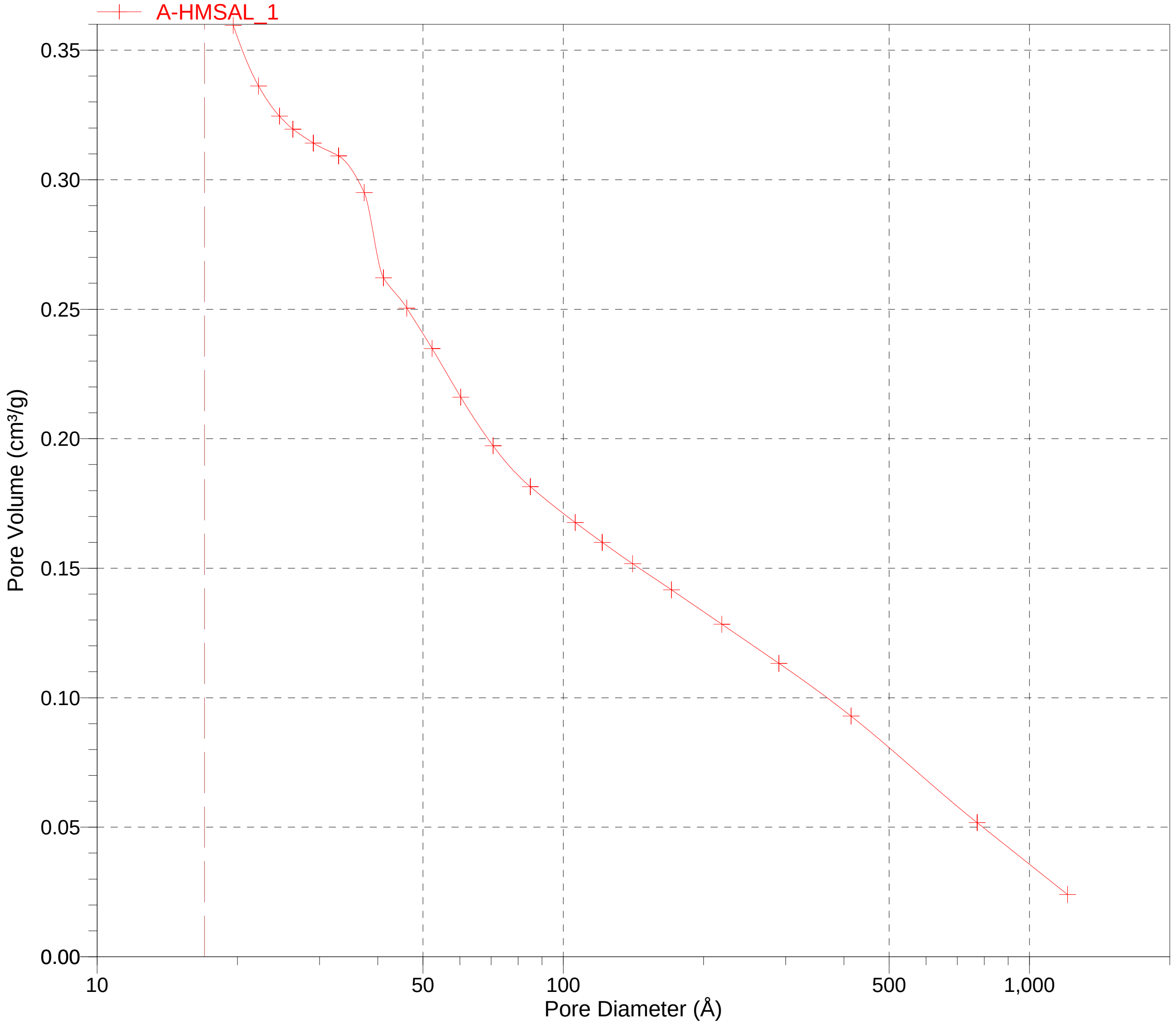

Sample: A-HMSAL_1
Operator: dianat
File: C:\2020\DATA\A-HMS.SMP

Started:	2020/08/21 10:26:34?.?	Analysis Adsorptive:	N2
Completed:	2020/08/22 5:24:58?.?	Analysis Bath Temp.:	77.225 K
Report Time:	2020/08/22 11:42:49?.?	Thermal Correction:	No
Sample Mass:	0.0970 g	Warm Free Space:	26.0524 cm³ Measured
Cold Free Space:	79.4615 cm³	Equilibration Interval:	10 s
Low Pressure Dose:	None	Automatic Degas:	Yes

BJH Desorption dV/dD Pore Volume

Halsey : Faas Correction

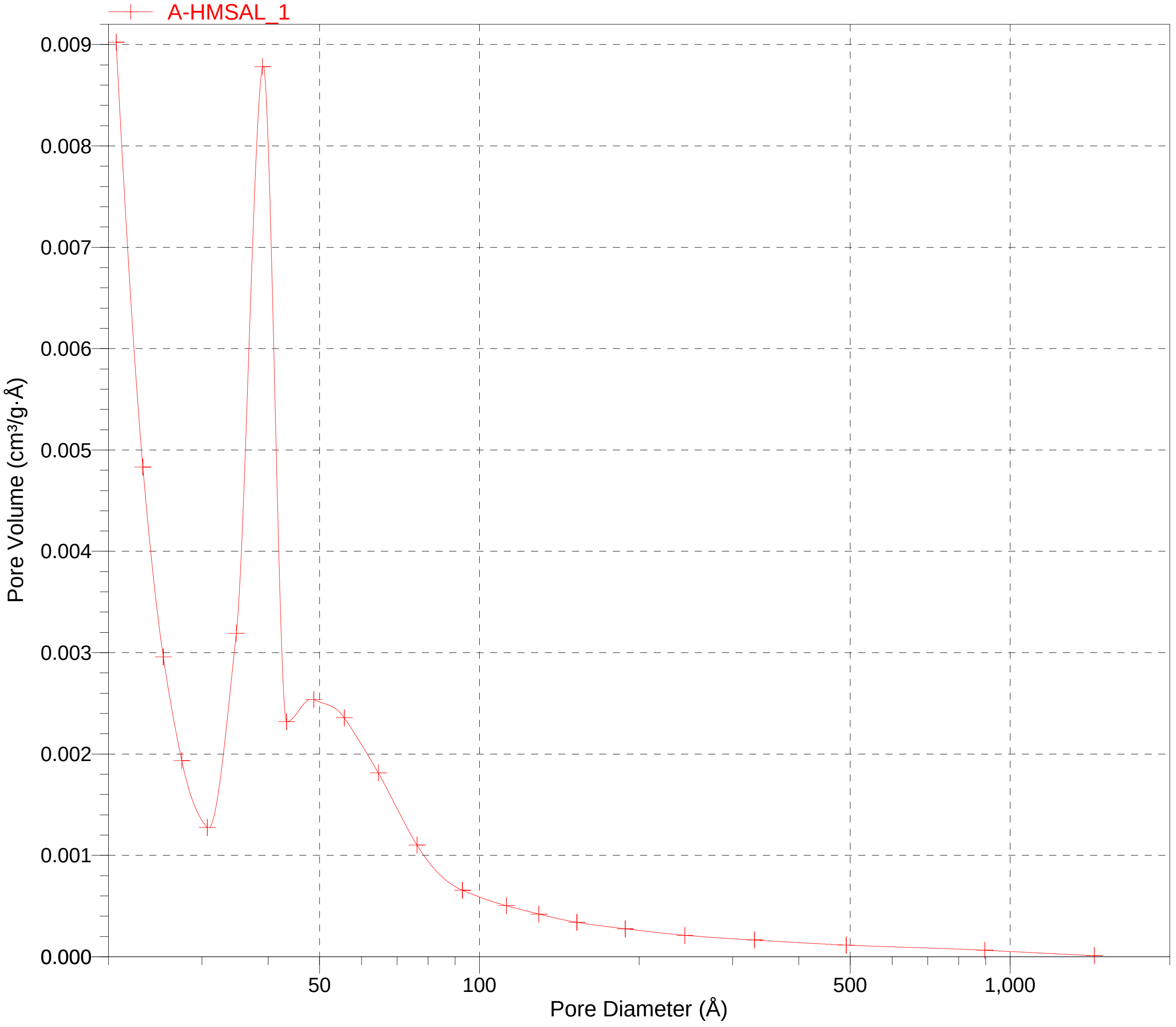

Sample: A-HMSAL_1
Operator: dianat
File: C:\2020\DATA\A-HMS.SMP

Started:	2020/08/21 10:26:34?.?	Analysis Adsorptive:	N2
Completed:	2020/08/22 5:24:58?.?	Analysis Bath Temp.:	77.225 K
Report Time:	2020/08/22 11:42:49?.?	Thermal Correction:	No
Sample Mass:	0.0970 g	Warm Free Space:	26.0524 cm³ Measured
Cold Free Space:	79.4615 cm³	Equilibration Interval:	10 s
Low Pressure Dose:	None	Automatic Degas:	Yes

BJH Desorption dV/dlog(D) Pore Volume

Halsey : Faas Correction

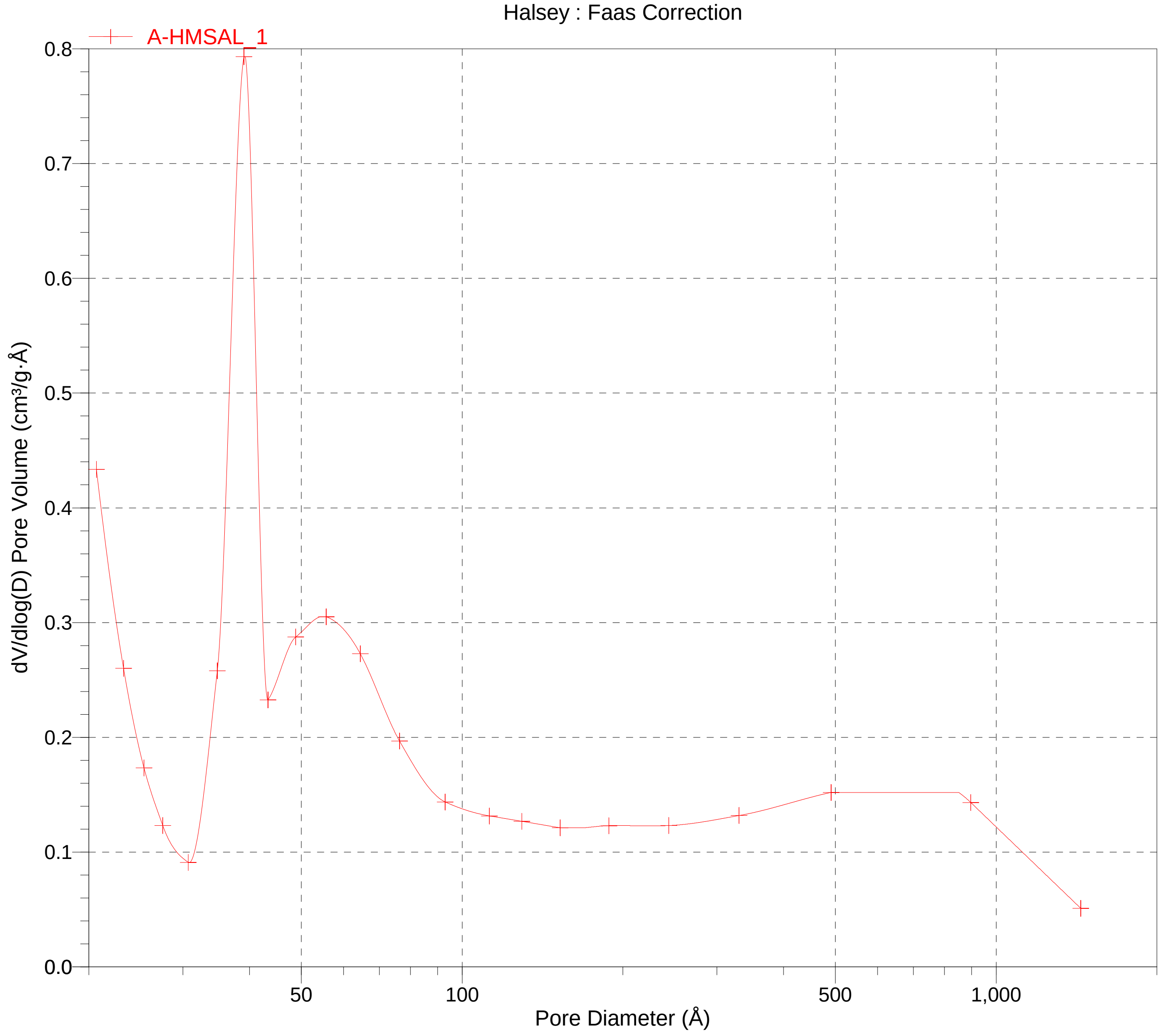

Sample: A-HMSAL_1
Operator: dianat
File: C:\2020\DATA\A-HMS.SMP

Started: 2020/08/21 10:26:34?.?
Completed: 2020/08/22 5:24:58?.?
Report Time: 2020/08/22 11:42:49?.?
Sample Mass: 0.0970 g
Cold Free Space: 79.4615 cm³
Low Pressure Dose: None

Analysis Adsorptive: N2
Analysis Bath Temp.: 77.225 K
Thermal Correction: No
Warm Free Space: 26.0524 cm³ Measured
Equilibration Interval: 10 s
Automatic Degas: Yes

BJH Desorption Cumulative Pore Area

Halsey : Faas Correction

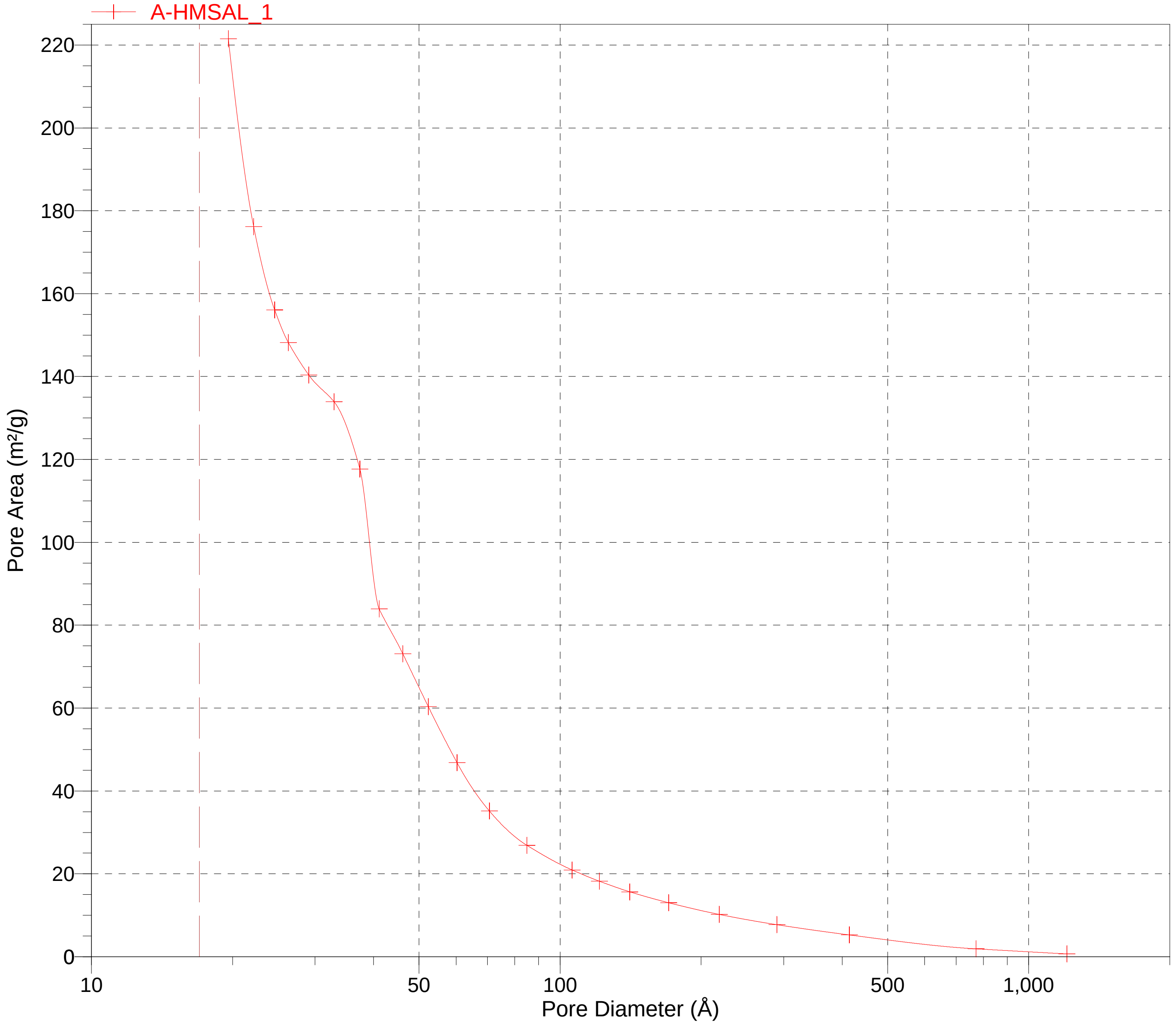

Sample: A-HMSAL_1
Operator: dianat
File: C:\2020\DATA\A-HMS.SMP

Started:	2020/08/21 10:26:34?.?	Analysis Adsorptive:	N2
Completed:	2020/08/22 5:24:58?.?	Analysis Bath Temp.:	77.225 K
Report Time:	2020/08/22 11:42:49?.?	Thermal Correction:	No
Sample Mass:	0.0970 g	Warm Free Space:	26.0524 cm³ Measured
Cold Free Space:	79.4615 cm³	Equilibration Interval:	10 s
Low Pressure Dose:	None	Automatic Degas:	Yes

BJH Desorption dA/dD Pore Area

Halsey : Faas Correction

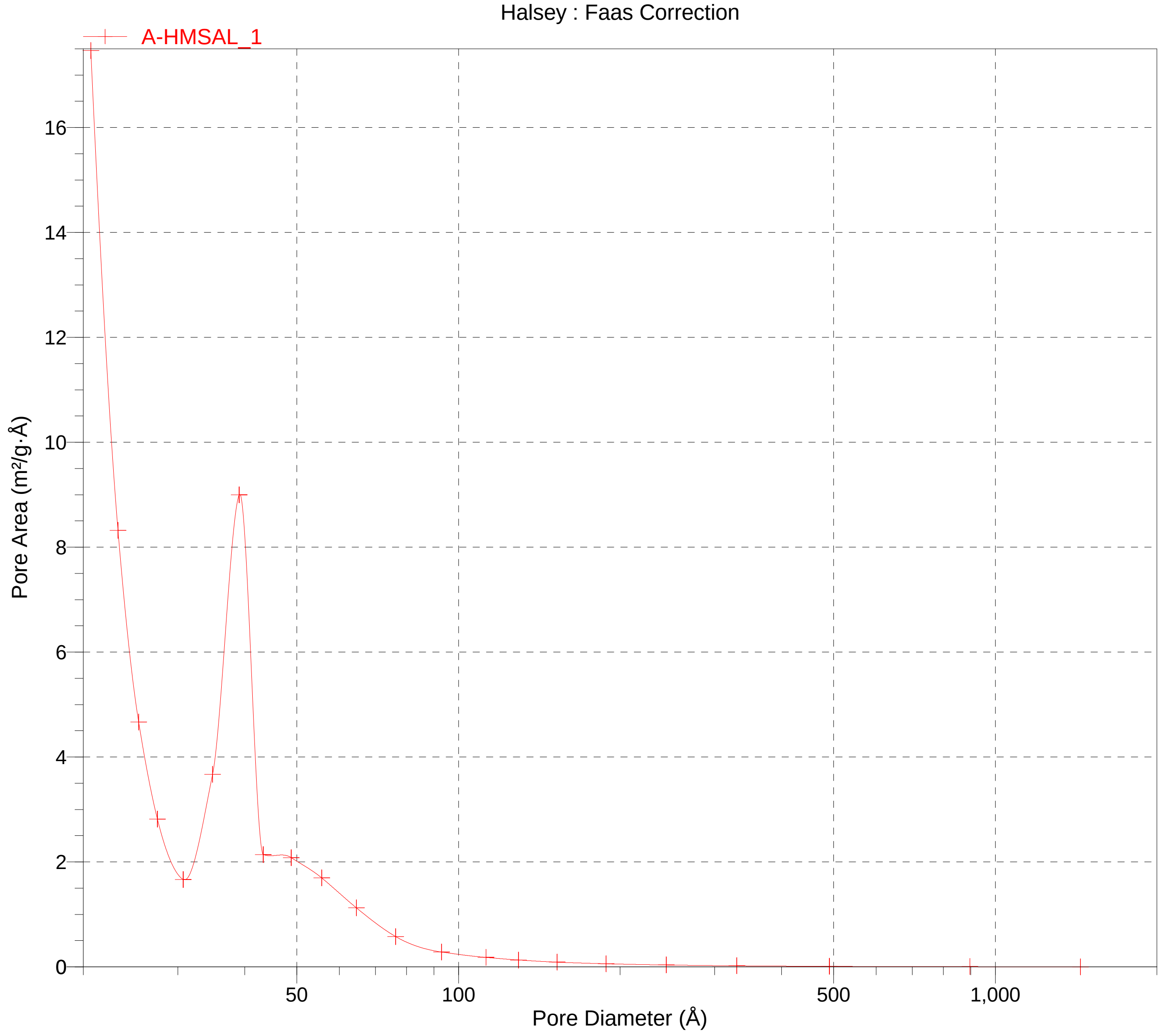

Sample: A-HMSAL_1
Operator: dianat
File: C:\2020\DATA\A-HMS.SMP

Started:	2020/08/21 10:26:34?.?	Analysis Adsorptive:	N2
Completed:	2020/08/22 5:24:58?.?	Analysis Bath Temp.:	77.225 K
Report Time:	2020/08/22 11:42:49?.?	Thermal Correction:	No
Sample Mass:	0.0970 g	Warm Free Space:	26.0524 cm³ Measured
Cold Free Space:	79.4615 cm³	Equilibration Interval:	10 s
Low Pressure Dose:	None	Automatic Degas:	Yes

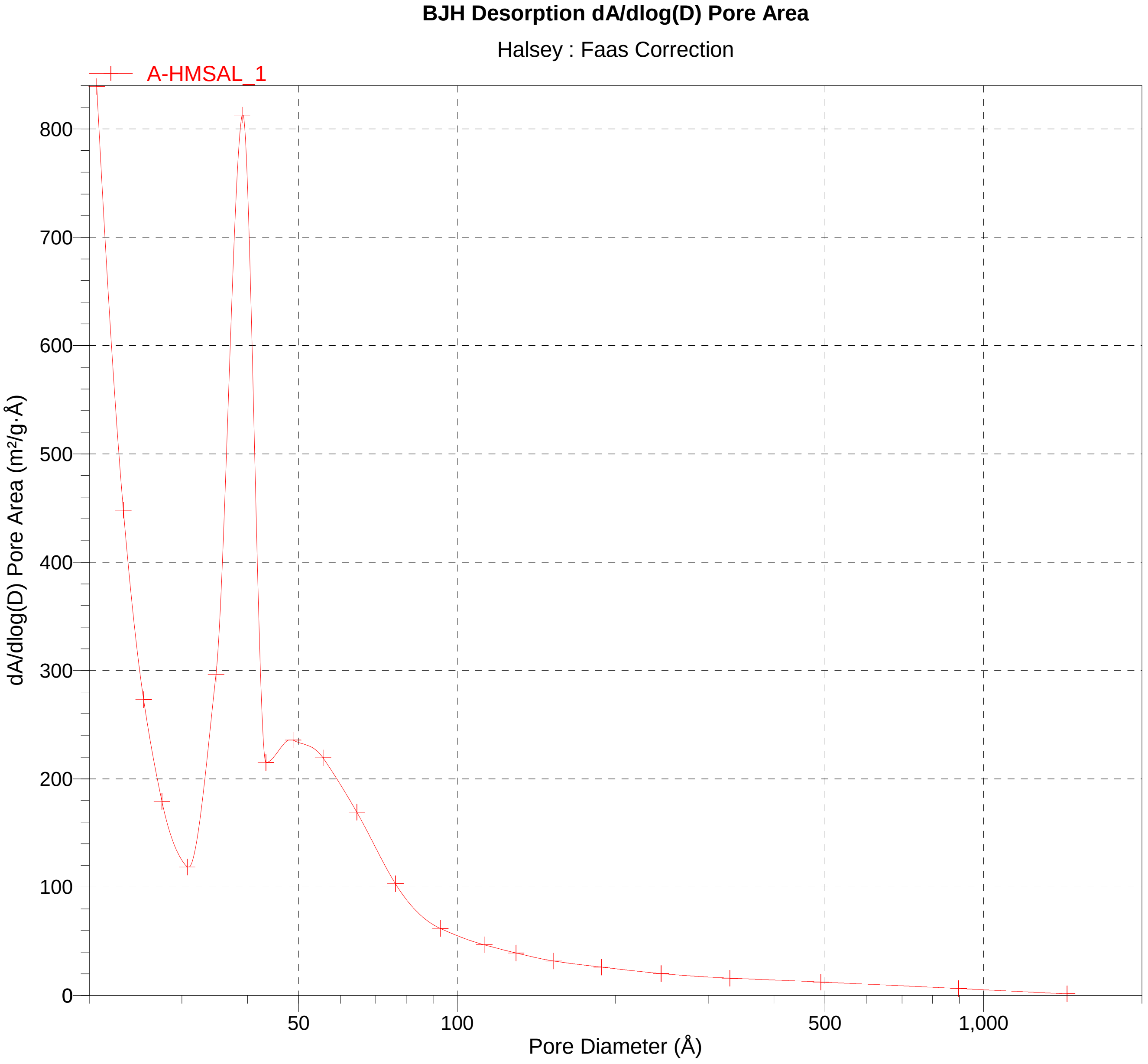

Sample: A-HMSAL_1
Operator: dianat
File: C:\2020\DATA\A-HMS.SMP

Started: 2020/08/21 10:26:34?.?
Completed: 2020/08/22 5:24:58?.?
Report Time: 2020/08/22 11:42:49?.?
Sample Mass: 0.0970 g
Cold Free Space: 79.4615 cm³
Low Pressure Dose: None

Analysis Adsorptive: N2
Analysis Bath Temp.: 77.225 K
Thermal Correction: No
Warm Free Space: 26.0524 cm³ Measured
Equilibration Interval: 10 s
Automatic Degas: Yes

Options Report

Sample Tube

Warm free space: 27.9457 cm³
Cold free space: 84.0511 cm³
Non-ideality factor: 0.0000620
Use Isothermal Jacket: No
Use Filler Rod: No
Vacuum seal type: Seal Frit

Analysis Conditions

Preparation

Fast evacuation: No
Unrestricted evacuation from: 0.67 kPa
Vacuum setpoint: 1.3 Pa
Evacuation time: 0.10 h
Leak test: Yes
Leak test duration: 120 s
Use TranSeal: No

Free Space

Free-space type: Measured
Lower dewar for evacuation: Yes
Evacuation time: 0.10 h
Outgas test: No

p° and Temperature

p° and T type: Measure p° at intervals during analys is. Calculate the Analysis Bath Temperature from these values.
Measurement interval: 120 min

Dosing

Use first pressure fixed dose: No
Use maximum volume increment: No
Target tolerance: 5.0% or 0.6666 kPa
Low pressure dosing: No

Equilibration

Equilibration time (p/p°= 1.000000000): 10 s
Minimum equilibration delay at p/p°>= 0.995: 600 s

Sample Backfill

Backfill at start of analysis: Yes
Backfill at end of analysis: Yes
Backfill gas: N2

Adsorptive Properties

Adsorptive: Nitrogen
Maximum manifold pressure: 123.323 kPa
Non-ideality factor: 0.0000620
Density conversion factor: 0.0015468
Therm. tran. hard-sphere diameter: 3.860 Å
Molecular cross-sectional area: 0.162 nm²

Inside diameter of sample tube: 9.53 mm
