# Supplementary material for: Preparation and catalytic application of two different nanocatalysts based on hexagonal mesoporous silica (HMS) in synthesis of tetrahydrobenzo[b]pyran and 1,4-dihydropyrano[2,3-c]pyrazole derivatives
Source: Sci Rep. 2022 Dec 21;12:22108. doi: 10.1038/s41598-022-26605-0 (PMC9772405; doi:10.1038/s41598-022-26605-0)
Supplement: Supplementary file 7 — Supplementary Information 7. [file 41598_2022_26605_MOESM7_ESM.docx]

**Supporting Information**

**Preparation and catalytic application of two different nanocatalysts based on hexagonal mesoporous silica (HMS) in synthesis of tetrahydrobenzo[b]pyran and 1,4-dihydropyrano[2,3-c]pyrazole derivatives**

Sahar Abdolahi ^1^, Fatemeh Gholamian ^1^, Maryam Hajjami ^1,2^*

^1^Department of Chemistry, Faculty of Science, Ilam University, P.O. Box 69315516, Ilam, Iran

^2^Department of Organic Chemistry, Faculty of Chemistry, Bu-Ali Sina University, Hamedan 6517838683, Iran, E-mail address, [m.hajjami@basu.ac.ir](mailto:m.hajjami@basu.ac.ir) or [mhajjami@yahoo.com](mailto:mhajjami@yahoo.com)

**General procedure for the synthesis of tetrahydrobenzo[b]pyran with** **HMS/Pr-Xa-Ni**

A test tube including aldehyde (1 mmol), dimedone (1 mmol), malononitrile (1 mmol) and HMS/Pr-Xa-Ni (0.04 g) were mixed in H_2_O: EtOH (3:1 mL) at 80 °C. Completion of the reaction was monitored by TLC. Then the hot EtOH was added to reaction mixture and the catalyst was separated by filtration. Purification of products was done with recrystallization in EtOH.

**General procedure for the synthesis of tetrahydrobenzo[b]pyran with HMS/Pr-PTSC-Cu**

A mixture of aldehyde (1 mmol), dimedone (1 mmol), malononitrile (1 mmol) and 0.004 g of catalyst (HMS/Pr-PTSC-Cu) in EtOH were added to a test tube and stirred with a magnetic stirrer at room temperature. Progress of reaction was monitored by TLC. After the completion of reaction the hot EtOH was added and catalyst was separated by filtration. Purification of products was done through recrystallization in EtOH.

|  |
| --- |
| 2–Amino–3–cyano–7,7–dimethyl–4-(3-nitrophenyl)–5–oxo-4H–5,6,7,8-tetrahydro benzopyran: ^1^H NMR (300 MHz, DMSO-d6): δ= 0.95 (s, 3H), 1.03 (s, 3H), 2.10 (d, J= 15 Hz, 1H), 2.26 (d, J= 15 Hz, 1H), 2.54 (s, 2H), 4.41 (s, 1H), 7.16 (s, 2H), 7.58-7.68 (m, 2H), 7.97 (s, 1H), 8.05-8.08 (m, 1H) ppm. |

|  |
| --- |
| 2–Amino–3–cyano–7,7–dimethyl–4-(2,4-dichlorophenyl)–5–oxo-4H–5,6,7,8-tetrahydro benzopyran: ^1^H NMR (300 MHz, DMSO-d6): δ= 0.97 (s, 3H), 1.03 (s, 3H), 2.07 (d, J= 15 Hz, 1H), 2.23 (d, J= 15 Hz, 1H), 3.16-3.45 (m, 2H), 4.68 (s, 1H), 7.07 (s, 2H), 7.21 (d, J=9 Hz, 1H), 7.34 (dd, J=9 Hz, 1H), 7.50 (d, J=3 Hz, 1H) ppm. |

|  |
| --- |
| 2–Amino–3–cyano–7,7–dimethyl–4-(3-hydroxyphenyl)–5–oxo-4H–5,6,7,8-tetrahydro benzopyran: ^1^H NMR (300 MHz, DMSO-d6): δ= 0.96 (s, 3H), 1.03 (s, 3H), 2.09 (d, J= 15 Hz, 1H), 2.24 (d, J= 15 Hz, 1H), 2.42-2.56 (m, 2H), 4.05 (s, 1H), 6.53 (s, 2H), 6.54-6.57 (m, 1H), 6.97-7.07 (m, 3H), 9.31 (s, 1H) ppm. |

|  |
| --- |
| 2–Amino–3–cyano–7,7–dimethyl–4-(3-nitrophenyl)–5–oxo-4H–5,6,7,8-tetrahydro benzopyran (Table 4, entry 4): IR (KBr, ν) 3433, 3334, 3202, 2958, 2878, 2189,1668, 1529, 1358, 1208, 1091,1033, 819 cm^-1^. |

|  |
| --- |
| 2–Amino–3–cyano–7,7–dimethyl–4-(4-methylphenyl)–5–oxo-4H–5,6,7,8-tetrahydro benzopyran (Table 8, entry 7): IR (KBr, ν) 3449, 3381, 3316, 2961, 2899, 2192, 1655, 1604, 1367, 1249, 1210, 1146, 1030, 765, 559 cm^-1^. |

|  |
| --- |
| 2–Amino–3–cyano–7,7–dimethyl–4-(phenyl)–5–oxo-4H–5,6,7,8-tetrahydro benzopyran (Table 8, entry 8): IR (KBr, ν) 3394, 3324, 3251, 2966, 2881, 2197, 1670, 1603, 1370, 1250, 1212, 1148, 1031, 738, 494 cm^-1^. |

|  |
| --- |
| 4,4'-(1,4-phenylene)bis(2-amino-7,7-dimethyl-5-oxo-5,6,7,8-tetrahydro-4H-chromene-3-carbonitrile) (Table 8, entry 11): IR (KBr, ν) 3639, 3460, 3326, 3191, 2958, 2879, 2193, 1680, 1597, 1467,1365,1252, 1211, 1149, 1031, 824, 564 cm^-1^. |

**General procedure for the synthesis of 1,4-dihydropyrano[2,3-c]pyrazole with** **HMS/Pr-Xa-Ni**

0.01 g of HMS/Pr-Xa-Ni was added to a mixture of aldehyde (1 mmol), hydrazine hydrate (1 mmol), ethyl acetoacetate (1 mmol) and malononitrile (1 mmol) in H_2_O: EtOH (2:1 mL) at 35 °C. Completion of the reaction was checked by TLC, then the catalyst was separated with filtration and washed with hot EtOH. Recrystallization with EtOH was applied to afford the pure products.

**General procedure for the synthesis of 1,4-dihydropyrano[2,3-c]pyrazole with HMS/Pr-PTSC-Cu**

0.006 g of HMS/Pr-PTSC-Cu was added to a mixture of aldehyde (1 mmol), hydrazine hydrate (1 mmol), ethyl acetoacetate (1 mmol) and malononitrile (1 mmol) in H_2_O: EtOH (2:1 mL) at room temperature. Completion of the reaction was continuously checked by TLC. After the consumption of the starting material and separation of catalyst, ethyl acetate and H_2_O were added and extracted. The organic layer was dried over Na_2_SO_4._ At the end, the ethyl acetate was evaporated to afford the corresponding product. Finally, purification of products undertake through recrystallization with EtOH.

|  |
| --- |
| 6-Amino-4-(2,4-dichlorophenyl)-3-methyl-2,4-dihydropyrano[2,3-c]pyrazole-5-carbonitrile: ^1^H NMR (300 MHz, DMSO-d6): δ= 1.77 (s, 3H), 5.05 (s, 1H), 6.98 (s, 2H), 7.21 (d, J= 9 Hz, 1H), 7.39 (dd, J= 3 Hz, 1H), 7.56 (d, J= 3 Hz, 1H) 12.15 (s, 1H) ppm. |

|  |
| --- |
| 6-Amino-3-methyl-4-(thiophen-2-yl)-2,4-dihydropyrano[2,3-c]pyrazole-5-carbonitrile: ^1^H NMR (300 MHz, DMSO-d6): δ=1.91 (s, 3H), 4.98 (s, 1H), 6.91–6.94 (m, 3H), 6.99-7.37 (m, 2H), 12.15 (s, 1H) ppm. |

|  |
| --- |
| 6-Amino-4-(3-hydroxyphenyl)-3-methyl-2,4-dihydropyrano[2,3-c]pyrazole-5-carbonitrile: ^1^H NMR (300 MHz, DMSO-d6): δ= 1.81 (s, 3H), 4.48 (s, 1H), 6.54–6.63 (m, 3H), 6.83 (s, 2H), 7.09 (t, J= 9 Hz, 1H), 9.29 (s,1H), 12.07 (s, 1H) ppm. |

|  |
| --- |
| 6-Amino-4-(4-bromophenyl)-3-methyl-1,4-dihydropyrano[2,3-c]pyrazole-5-carbonitrile: ^1^H NMR (300 MHz, DMSO-d6): δ= 1.78 (s, 3H), 4.61 (s, 1H), 6.92 (s, 2H), 7.12 (d, J= 6 Hz, 2H), 7.50 (d, J= 6 Hz, 2H), 12.13 (s, 1H) ppm. |

|  |
| --- |
| 6-Amino-4-(2-hydroxyphenyl)-3-methyl-2,4-dihydropyrano[2,3-c]pyrazole-5-carbonitrile (Table 10, entry 8): IR (KBr, ν) 3613, 3446, 3351, 2187, 1660, 1612, 1531, 1401, 755, 497 cm^-1^. |

|  |
| --- |
| 6-Amino-4-(4-bromophenyl)-3-methyl-1,4-dihydropyrano[2,3-c]pyrazole-5-carbonitrile (Table 10, entry 2): IR (KBr, ν) 3481, 3395, 3182, 2189, 1643, 1600, 1488, 1401, 1046, 798, 537 cm^-1^. |

**Synthesis of 3-(trimethoxysilyl)propyl (Z)-phenylcarbamohydrazonothioate**

A mixture of 4-phenylthiosemicarbazide (1 mmol), 3‐chloropropyltrimethoxysilane (1 mmol) and K_2_CO_3_ (2 mmol) in EtOH were added and stirred with a magnetic stirrer at reflux condition for 24 h. Then the obtained precipitation separated by filtration and washed with EtOH. Purification of product was done through recrystallization in EtOH

|  |
| --- |
| 3-(trimethoxysilyl)propyl (Z)-phenylcarbamohydrazonothioate  IR (KBr, ν) 3416, 2924, 2857, 1627, 1506, 1417, 1130, 1033, 760, 690, 474 cm^-1^.  ^1^H NMR (300 MHz, DMSO-d6): 0.68 (s, 2H), 1.71 (s, 2H), 3.11 (s, 2H), 3.33-3.61 (m, 9H), 7.43-7.56 (m, 5H), 8.31 (s, 1H), 8.84 (s, 1H) ppm.  ^13^C NMR (300 MHz, DMSO-d6): 20.9, 25.7, 47.3, 67.9, 126.0, 129.0, 132.5, 143.8, 147.8 ppm. |

**The ^1^H-NMR of 2–amino–3–cyano–7,7–dimethyl–4-(3-nitrophenyl)–5–oxo-4H–5,6,7,8-tetrahydro benzopyran**

|  |
| --- |
| 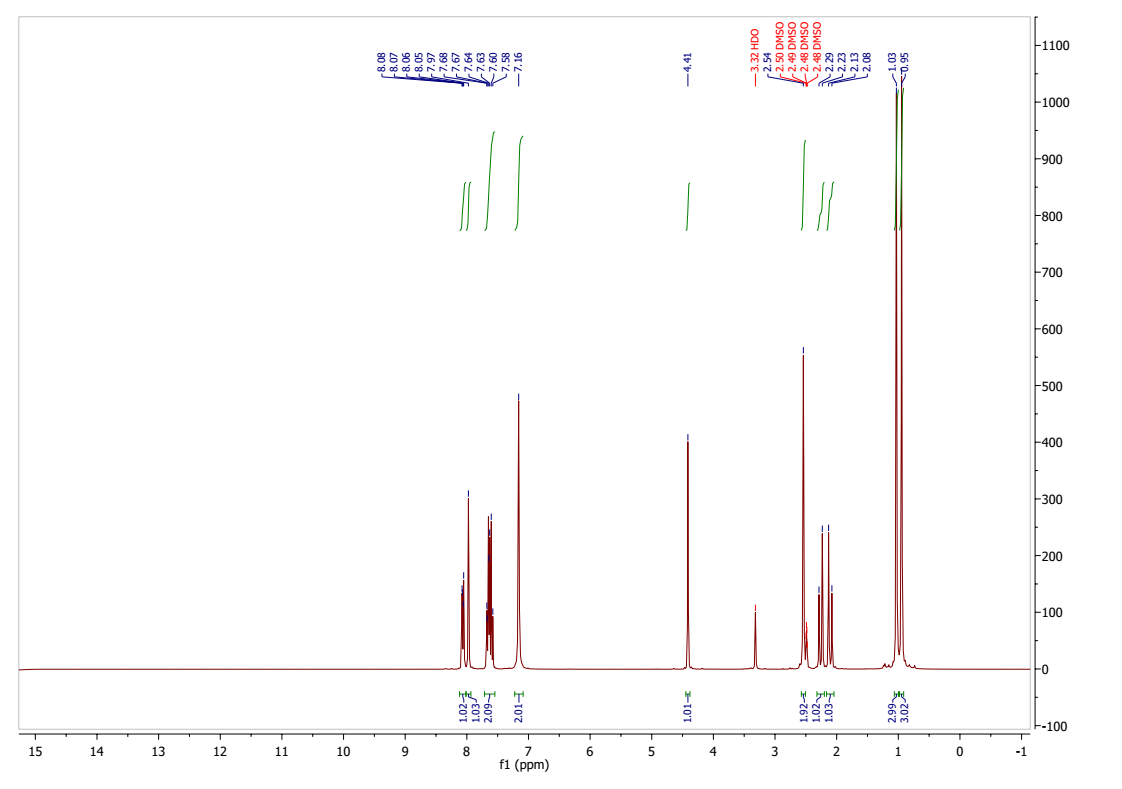 |

**The ^1^H-NMR of 2–amino–3–cyano–7,7–dimethyl–4-(2,4-dichlorophenyl)–5–oxo-4H–5,6,7,8-tetrahydro benzopyran**

|  |
| --- |
| 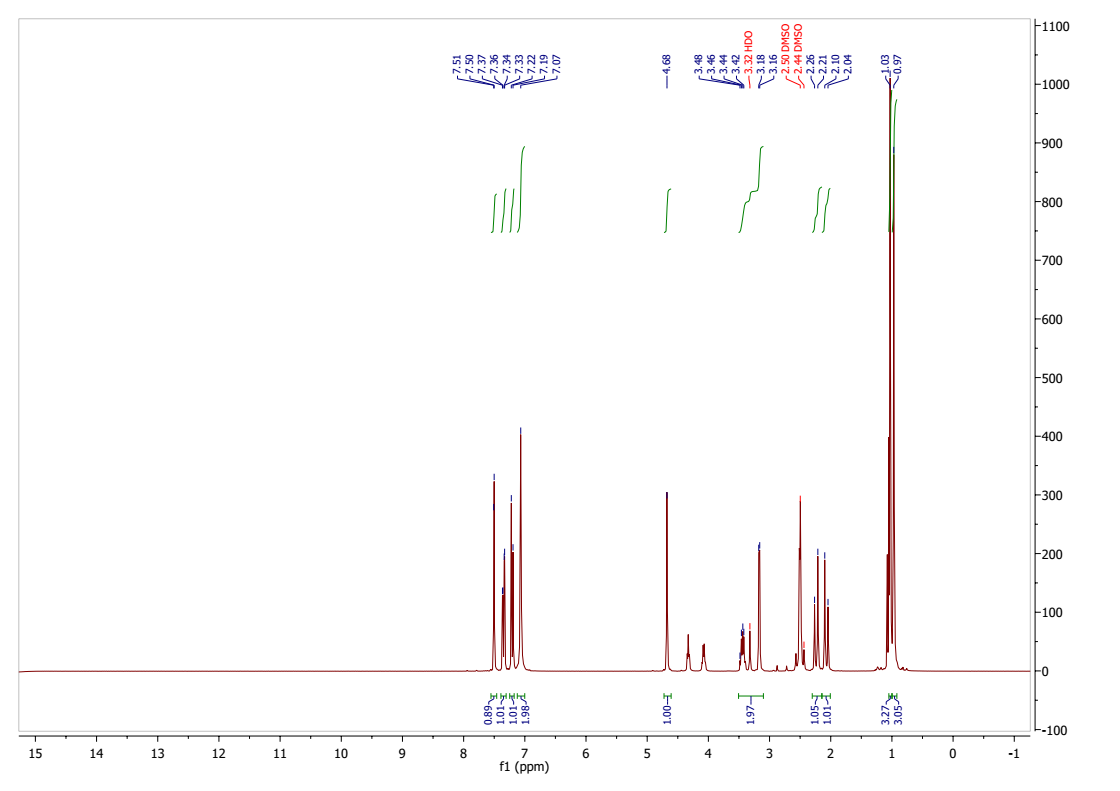 |

**The ^1^H-NMR of** **2–amino–3–cyano–7,7–dimethyl–4-(3-hydroxyphenyl)–5–oxo-4H–5,6,7,8-tetrahydro benzopyran**

|  |
| --- |
| 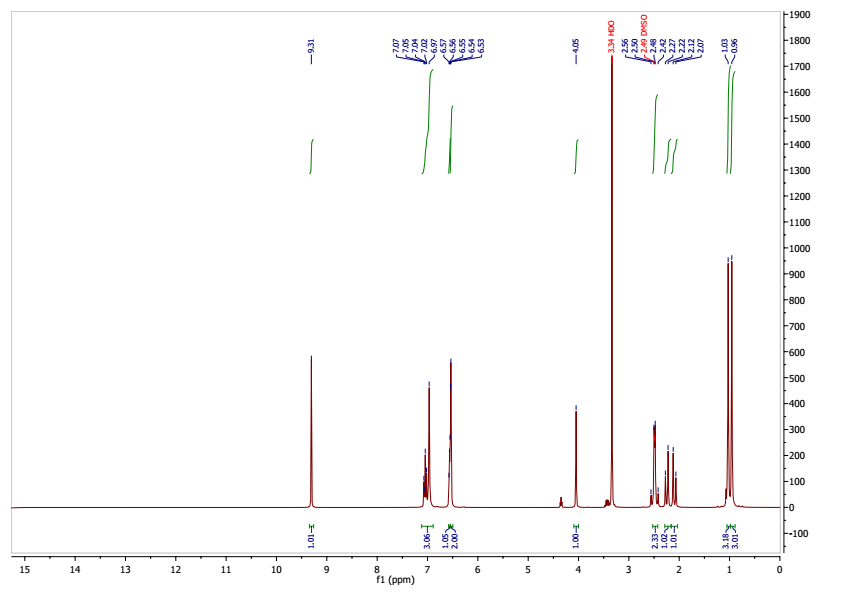 |

**The ^1^H-NMR of 6-amino-4-(2,4-dichlorophenyl)-3-methyl-2,4-dihydropyrano[2,3-c]pyrazole-5-carbonitrile**

|  |
| --- |
| 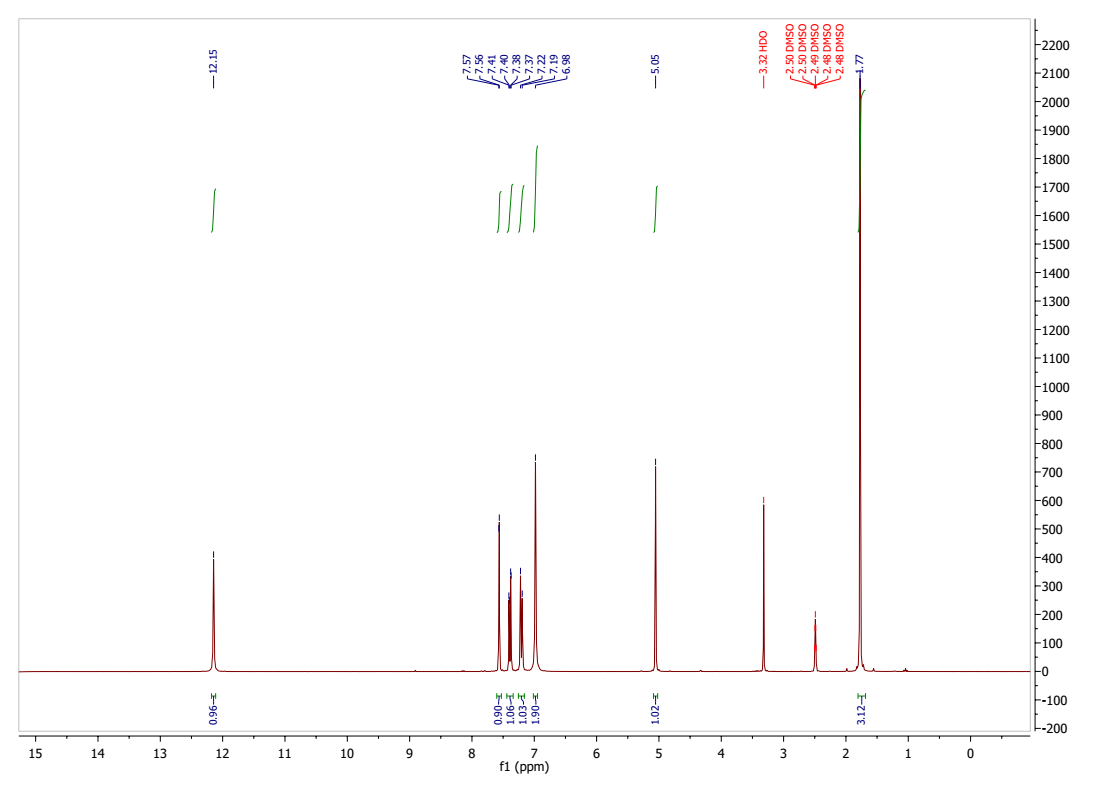 |

**The ^1^H-NMR of 6-amino-3-methyl-4-(thiophen-2-yl)-2,4-dihydropyrano[2,3-c]pyrazole-5-carbonitrile**

|  |
| --- |
| 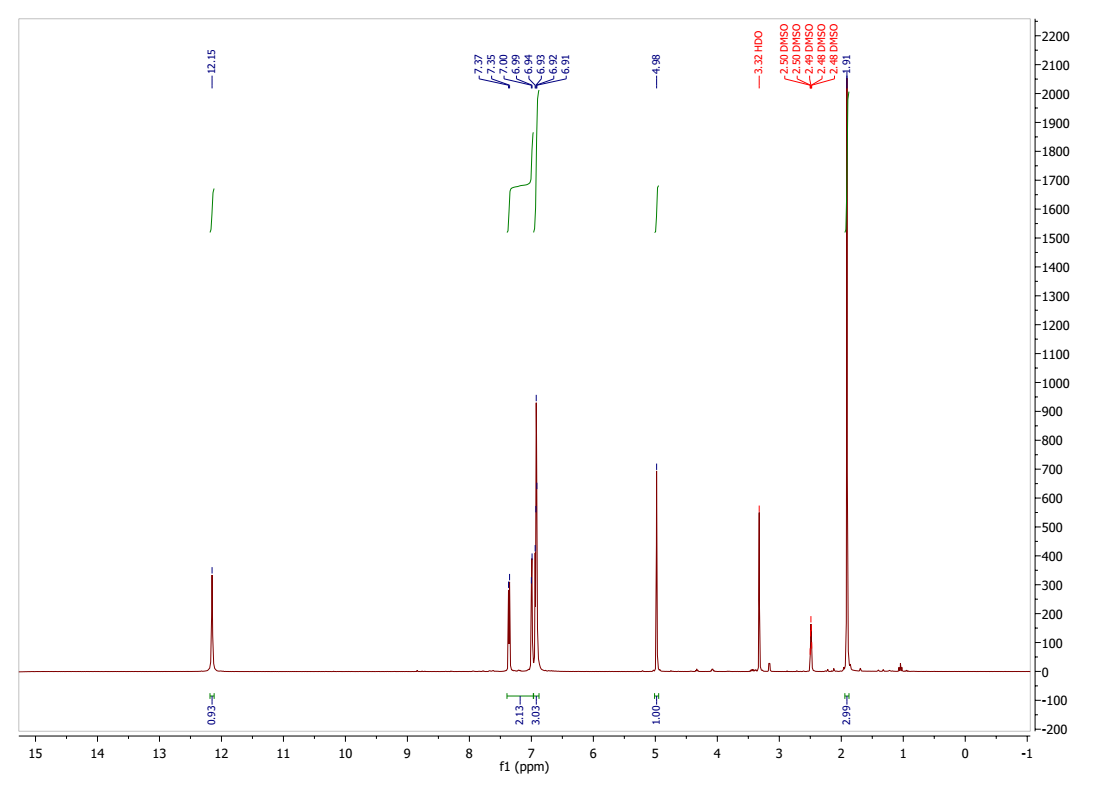 |

**The ^1^H-NMR of 6-amino-4-(3-hydroxyphenyl)-3-methyl-2,4-dihydropyrano[2,3-c]pyrazole-5-carbonitrile**

|  |
| --- |
| 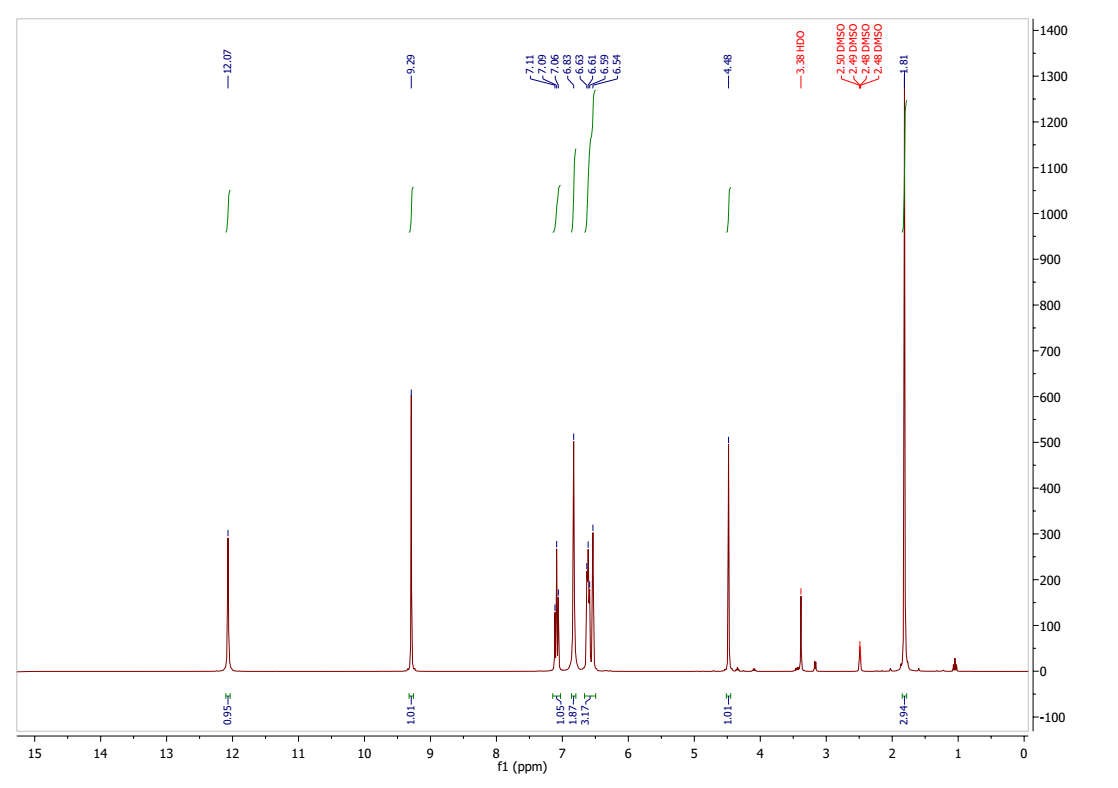 |

**The ^1^H-NMR of 6-amino-4-(4-bromophenyl)-3-methyl-1,4-dihydropyrano[2,3-c]pyrazole-5-carbonitrile**

|  |
| --- |
| 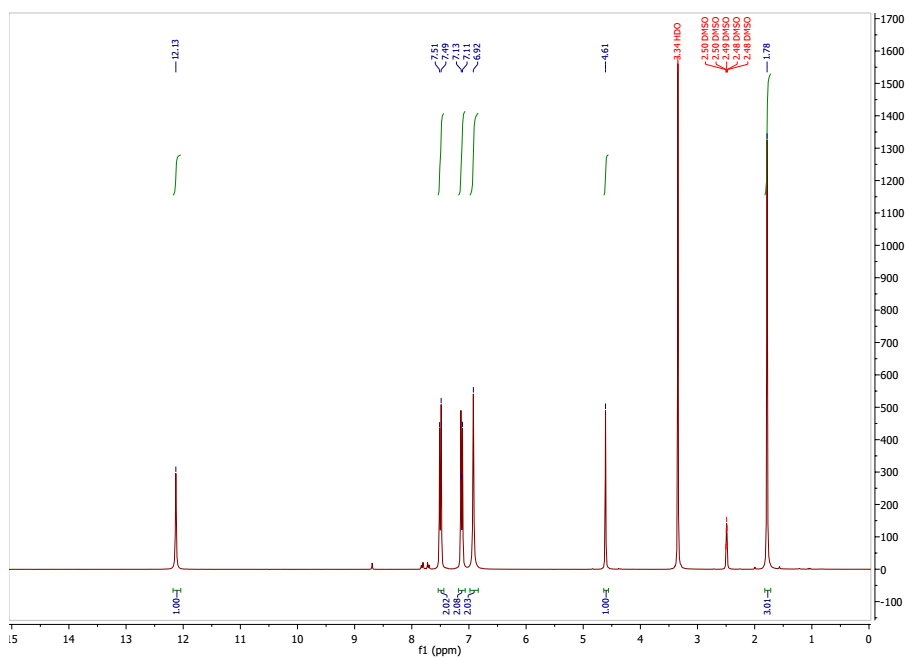 |

**The IR of 2–amino–3–cyano–7,7–dimethyl–4-(3-nitrophenyl)–5–oxo-4H–5,6,7,8-tetrahydro benzopyran**

|  |
| --- |
| 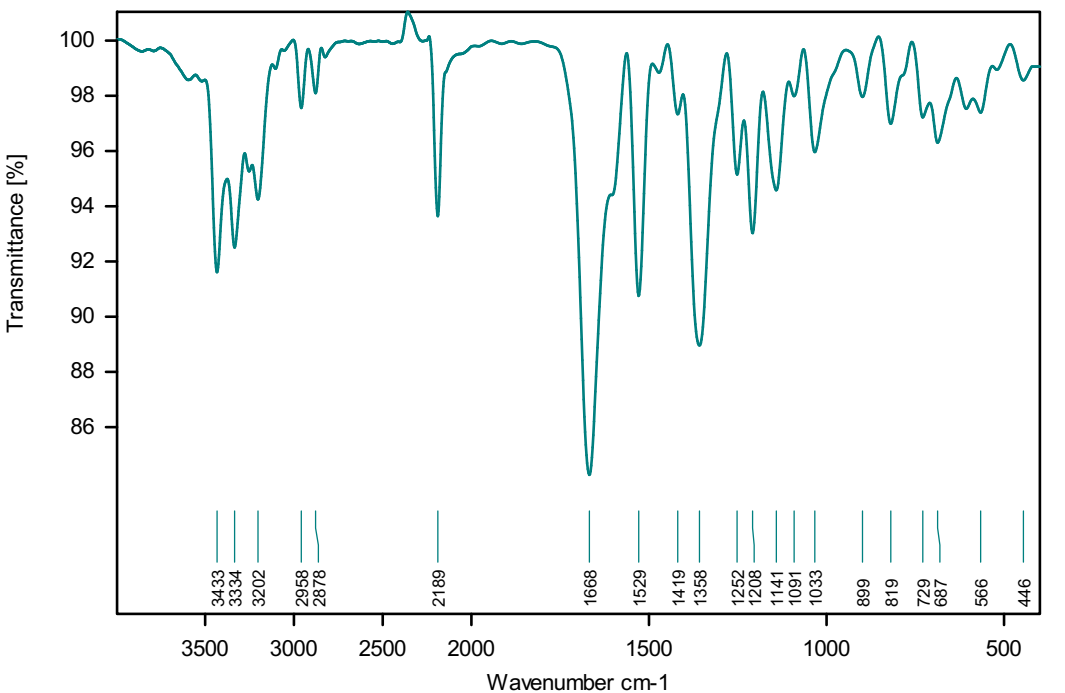 |

**The IR of 2–amino–3–cyano–7,7–dimethyl–4-(4-methylphenyl)–5–oxo-4H–5,6,7,8-tetrahydro**

|  |
| --- |
| 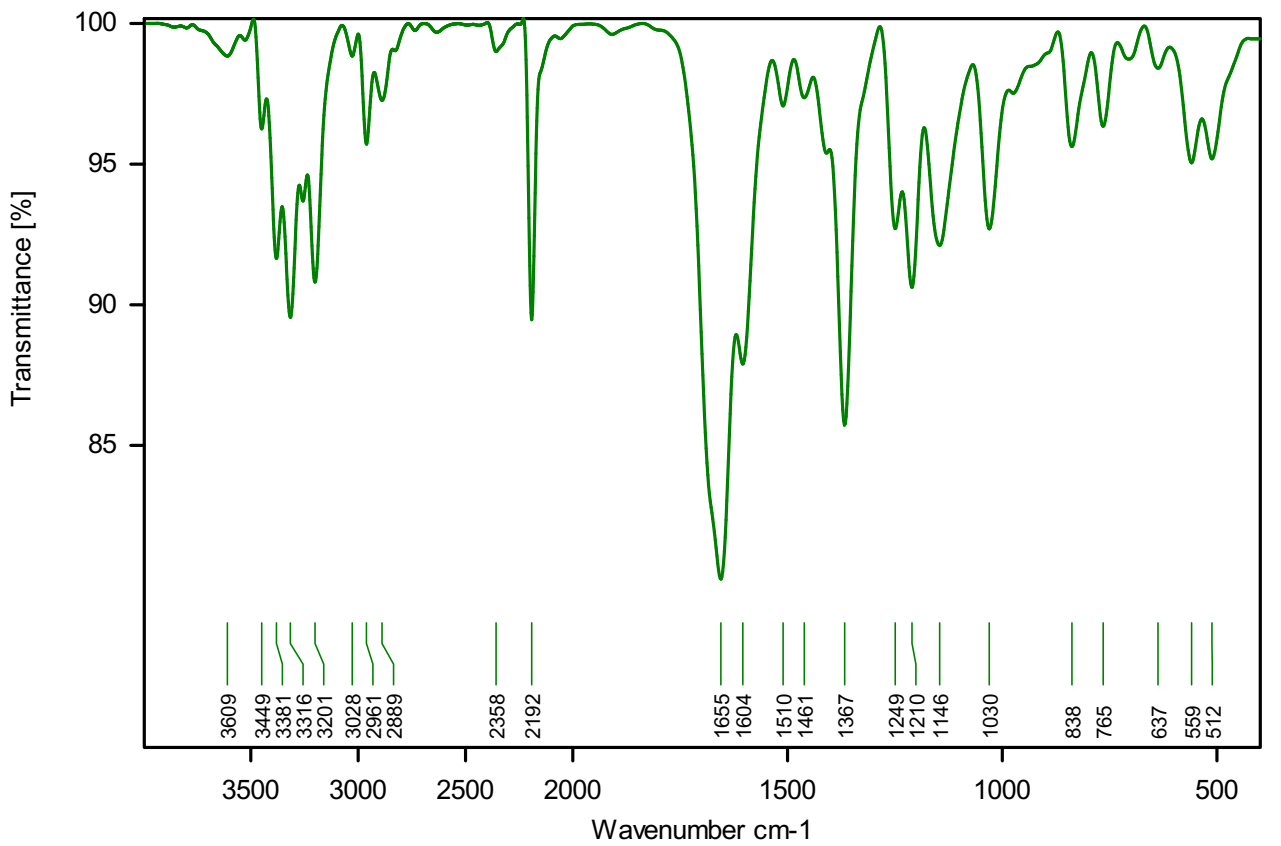 |

**The IR of 2–amino–3–cyano–7,7–dimethyl–4-(phenyl)–5–oxo-4H–5,6,7,8-tetrahydro benzopyran**

|  |
| --- |
| 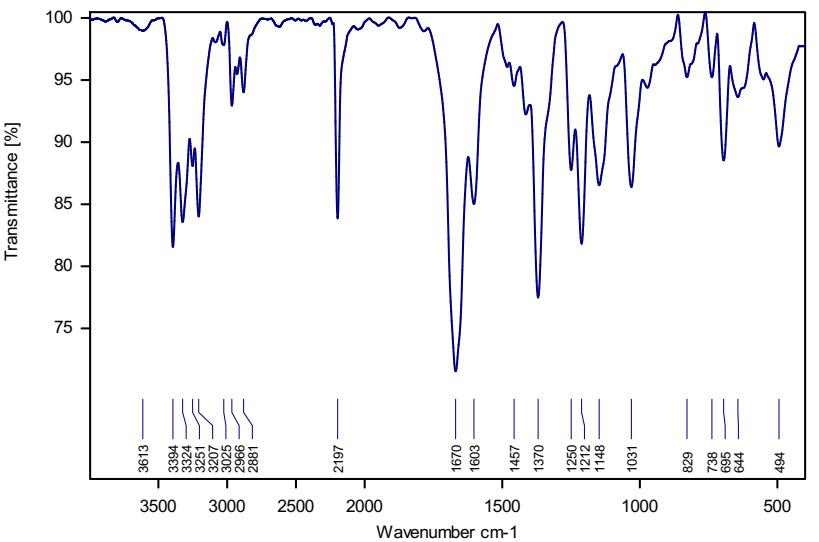 |

**The IR of 4,4'-(1,4-phenylene)bis(2-amino-7,7-dimethyl-5-oxo-5,6,7,8-tetrahydro-4H-chromene-3-carbonitrile)**

|  |
| --- |
| 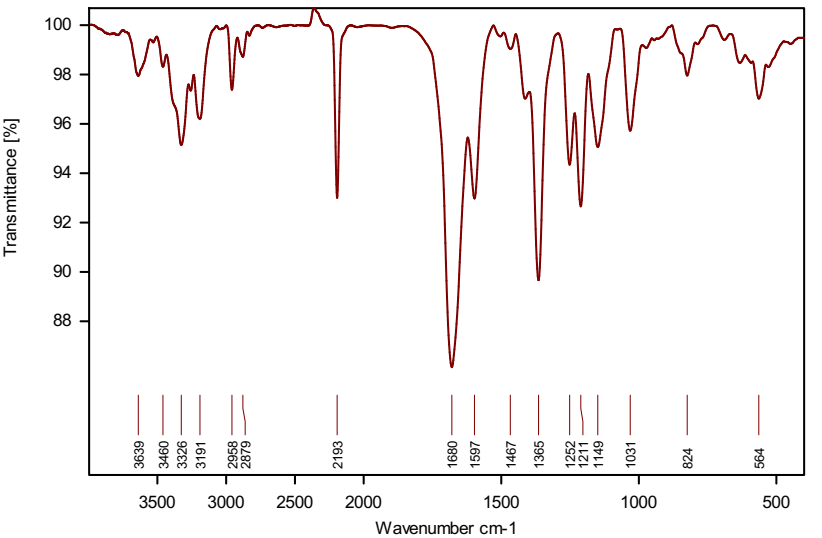 |

**The IR of 6-amino-4-(2-hydroxyphenyl)-3-methyl-2,4-dihydropyrano[2,3-c]pyrazole-5-carbonitrile**

|  |
| --- |
| 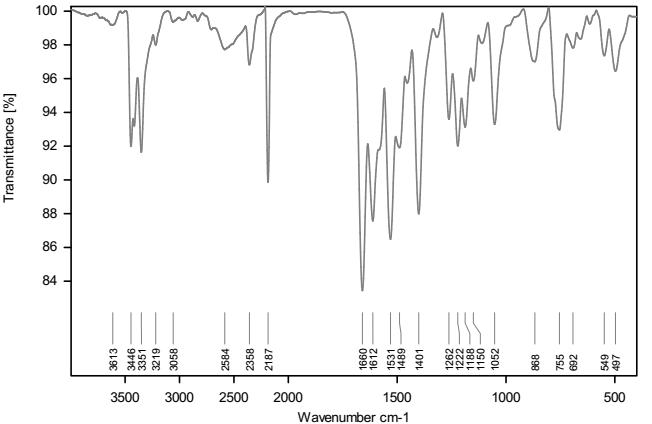 |

**The IR of 6-amino-4-(4-bromophenyl)-3-methyl-1,4-dihydropyrano[2,3-c]pyrazole-5-carbonitrile**

|  |
| --- |
| 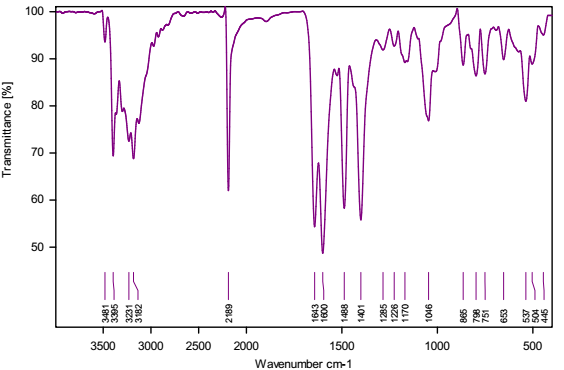 |

**The IR of 3-(trimethoxysilyl)propyl (Z)-phenylcarbamohydrazonothioate**

|  |
| --- |
| 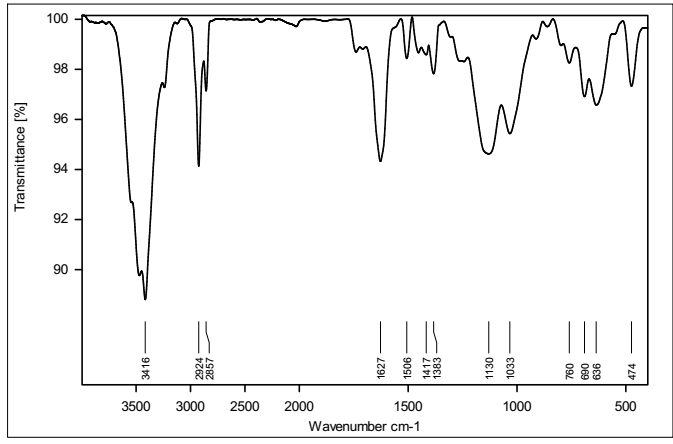 |

**The ^1^H-NMR of 3-(trimethoxysilyl)propyl (Z)-phenylcarbamohydrazonothioate**

|  |
| --- |
|  |

**The ^13^C-NMR of 3-(trimethoxysilyl)propyl (Z)-phenylcarbamohydrazonothioate**

|  |
| --- |
|  |
